# Density deficit of Earth’s core revealed by a multimegabar primary pressure scale

**DOI:** 10.1126/sciadv.adh8706

**Published:** 2023-09-08

**Authors:** Daijo Ikuta, Eiji Ohtani, Hiroshi Fukui, Tatsuya Sakamaki, Rolf Heid, Daisuke Ishikawa, Alfred Q. R. Baron

**Affiliations:** ^1^ Department of Earth Science, Tohoku University, Sendai, Miyagi 980-8578, Japan.; ^2^ Materials Dynamics Laboratory, RIKEN SPring-8 Center, Sayo, Hyogo 679-5148, Japan.; ^3^ Japan Synchrotron Radiation Research Institute, Sayo, Hyogo 679-5198, Japan.; ^4^ Institute for Quantum Materials and Technologies, Karlsruhe Institute of Technology, D-76021 Karlsruhe, Germany.

## Abstract

An accurate pressure scale is a fundamental requirement to understand planetary interiors. Here, we establish a primary pressure scale extending to the multimegabar pressures of Earth’s core, by combined measurement of the acoustic velocities and the density from a rhenium sample in a diamond anvil cell using inelastic x-ray scattering and x-ray diffraction. Our scale agrees well with previous primary scales and shock Hugoniots in each experimental pressure range and reveals that previous scales have overestimated laboratory pressures by at least 20% at 230 gigapascals. It suggests that the light element content in Earth’s inner core (the density deficit relative to iron) is likely to be double what was previously estimated, or Earth’s inner core temperature is much higher than expected, or some combination thereof.

## Teaser

A primary pressure scale to multimegabar pressure may double the light element content of Earth’s inner core.

## INTRODUCTION

Precise information about the composition of Earth’s core is critical for understanding planetary evolution (*1*–*3*) and discussing current important topics in geodynamic behavior, such as core-mantle boundary heat flow (*3*, *4*). However, samples from deep in the planetary interior are not available, so our knowledge is based on comparison of laboratory measurements (*5*–*8*) with seismological observations (*9*), information from meteorite composition (*3*), and indications of Earth’s core temperature (*10*–*12*). One of the most interesting results of such work has been the suggestion that Earth’s core must contain light elements because the density of the core, as determined from seismological observations (*9*), is lower than the density of pure iron, its main constituent, as determined by laboratory measurements (*5*–*7*) and theoretical work (*10*, *11*). However, this conclusion critically relies on having an accurate pressure scale to relate laboratory-generated pressures to geological pressures.

Establishing an accurate pressure scale has been the subject of intensive research (*13*–*20*), but present scales still rely on large extrapolation and approximations, especially at high pressures (*21*). Further, a pressure scale to multimegabar pressures is indispensable for discussing super-Earth planets (*22*, *23*). Previously, the compression curve for rhenium has been used as a secondary pressure scale determined on the basis of the pressure scales derived from shock compression measurements of several metals (*24*–*26*). The shock compression work, which occurs along a nonisothermal Hugoniot curve, is converted to an isothermal scale by the Rankine-Hugoniot equations with the Mie-Grüneisen-Debye (MGD) equation of state (EoS) (*1*). However, these derived scales show discrepancies of ~50% at density of ~33 g cm^−3^ (*26*). Other work in static conditions provides primary pressure scales based on thermodynamic relations that allow the pressure to be determined when the density and both acoustic velocities, longitudinal (or compressional, *v*_p_) and transverse (or shear, *v*_s_) waves, are measured (*13*–*20*). However, most of the static experiments have been limited to lower-mantle pressures (up to 55 GPa) (*13*–*19*) with only one recent result (*20*) extending to ~120 GPa, as is close to the core-mantle boundary pressure: The measurement techniques used in the previous work, Brillouin scattering measurements [single crystal of periclase (*13*, *14*) and polycrystalline sample of sodium chloride (*20*)], ultrasonic measurements [polycrystalline sample of wadsleyite (*15*), periclase (*16*), and tungsten (*19*)], and inelastic x-ray scattering (IXS) measurements [single crystal of platinum (*17*) and sodium chloride (*18*)] become increasingly difficult as pressure increases.

Here, we measure acoustic velocities (*v*_p_ and *v*_s_) of rhenium in a diamond anvil cell (DAC) under extreme pressure using IXS and in situ x-ray diffraction (XRD) at BL43LXU (*27*) of the RIKEN SPring-8 Center. The XRD measurements were performed in situ, with the same x-ray beam and probed sample volume used for the IXS measurements. The energy of the longitudinal acoustic (LA) and transverse acoustic (TA) modes was measured using IXS, determining *v*_p_ and *v*_s_, while in situ XRD was used to determine the density, ρ. A highly optimized setup with a 5-μm beam size and special optics to reduce backgrounds (*28*) allowed us to extend the range of our work in static conditions in a DAC to the multimegabar pressures of Earth’s core, 230 GPa in our rhenium scale, or what would be 274 to 300 GPa based on previous scales (*25*, *29*–*31*) (see also the “Starting material and high-pressure generation” to “Primary pressure scale derivation” sections in Methods).

## RESULTS

### Acoustic velocity measurement by IXS

**Fig. 1.**
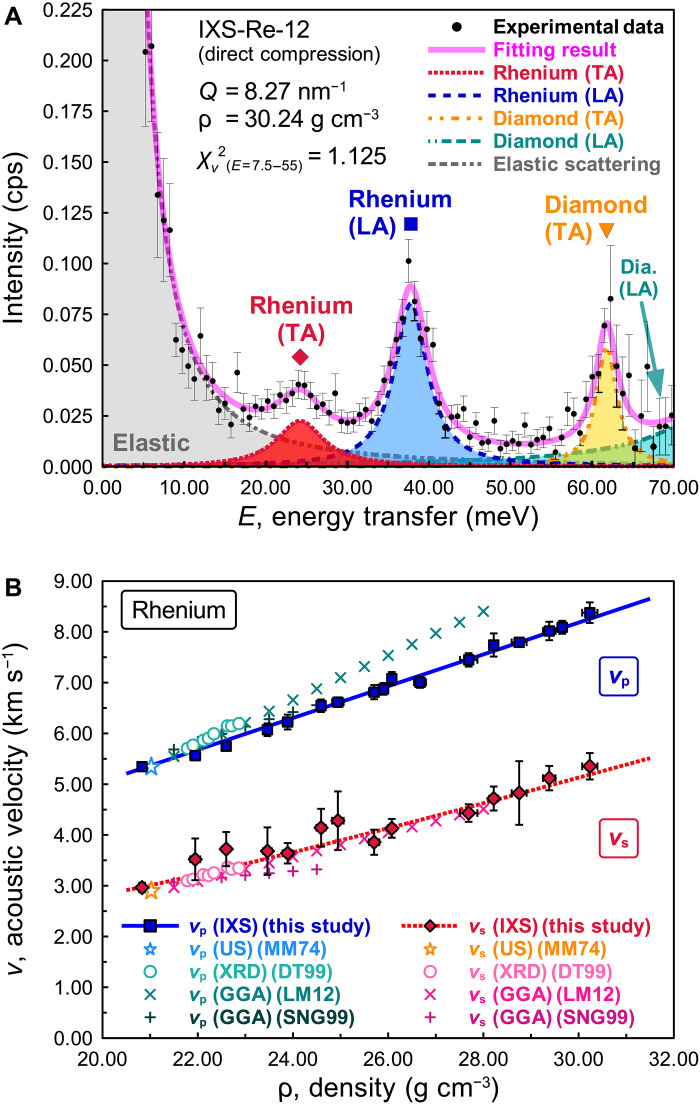
Results of acoustic velocity measurement for rhenium at high pressure. (**A**) IXS spectrum and fitting results for rhenium at density, ρ = 30.24 g cm^−3^ (230 GPa) and 300 K (IXS-Re-12). The black dots are the IXS data with 1 SD (1σ) error bars. Other colored lines and areas are individual inelastic contributions of LA and TA modes as labeled, with colored symbols showing the fitted peak positions. (**B**) Acoustic velocities (compressional, *v*_p_, and shear, *v*_s_) for rhenium as a function of density (table S1). The blue squares and red diamonds are *v*_p_ and *v*_s_ for rhenium determined from our IXS data with 1σ error bars. Other colored symbols are from previous studies [MM74 (*32*), DT99 (*35*), LM12 (*31*), and SNG99 (*36*)].

An example of an IXS spectrum measured from rhenium at 230 GPa (the highest pressure: IXS-Re-12) is shown in Fig. 1A and shows clear peaks that we identify as the being due to the TA and LA modes. Fits to the IXS spectra allow us to determine *v*_p_ and *v*_s_ of rhenium (fig. S1 and table S1). We also measured *v*_p_, *v*_s_, and ρ of rhenium at ambient conditions (in air) using a rhenium foil (fig. S2 and table S1) and confirmed that *v*_p_, *v*_s_, and ρ are consistent with the ultrasonic measurement (*32*) (see also note S1). The presence of the clear TA peak in the IXS spectrum was unexpected, as generally it should be weak in our small scattering angle geometry. This is discussed in note S2 and figs. S3 and S4, and we conclude that it is due to a large defect density that occurs when rhenium is compressed. Figure 1B shows the relations of ρ with *v*_p_ and *v*_s_ at high pressure and ambient temperature. High-pressure experiments in this study were performed both with and without a periclase (MgO) pressure medium and laser annealing. In the experiments with the MgO pressure medium and laser annealing, the rhenium sample was annealed at temperatures over 1000 K by a double-sided laser heating method (“Starting material and high-pressure generation” and “IXS measurement” sections in Methods) before the IXS-XRD measurements, to minimize the deviatoric stress. As shown in fig. S5A, the observed *c*/*a* ratios of the rhenium sample under nonhydrostatic conditions (direct compression, without pressure medium and laser annealing) were smaller than the calculated model *c*/*a* ratio of rhenium under hydrostatic pressure (*31*). However, both cases showed essentially similar acoustic velocities (fig. S5B). A detailed and careful analysis of the data, including the impact of the crystal preferred orientation, the lattice strain (LS), and other factors, may be found in notes S3 to S10 and figs. S6 to S14. We find that the observed preferred orientations, and LSs, have negligible impact on the acoustic velocity. This is consistent with previous studies for hexagonal close-packed (hcp) iron (*8*, *33*).

The ρ-*v*_p_ relation is well described by a linear function, Birch’s law (*34*) with

$$v_{p}=v_{p,0}+(\partial v_{p}/\partial \rho )(\rho -\rho_{0})$$ (1)

where we find ρ_0_ = 20.8(±0.1) × 10^3^ kg m^−3^, *v*_p,0_ = 5.30(±0.03) × 10^3^ m s^−1^, and ∂*v*_p_/∂ρ = 0.313(±0.002) m^4^ kg^−1^ s^−1^ for rhenium (see table S2). The subscript zero indicates ambient conditions. The ρ-*v*_s_ relation (red line in Fig. 1B) is derived from Eq. 1 with the MGD EoS (“Primary pressure scale derivation” section in Methods and table S2). Comparing our result to previous studies, we find that *v*_s_ in our study is consistent with the XRD-LS measurements (*35*) and the first-principles generalized gradient approximation (GGA) calculation of (*31*) but is not consistent with the GGA calculation of (*36*). Meanwhile, *v*_p_ in our study is consistent with the GGA calculations of (*36*), but we have a different trend compared with the XRD-LS measurements (*35*) and the GGA calculation of (*31*), especially at multimegabar pressures.

### Primary pressure scale of rhenium at multimegabar pressures

**Fig. 2.**
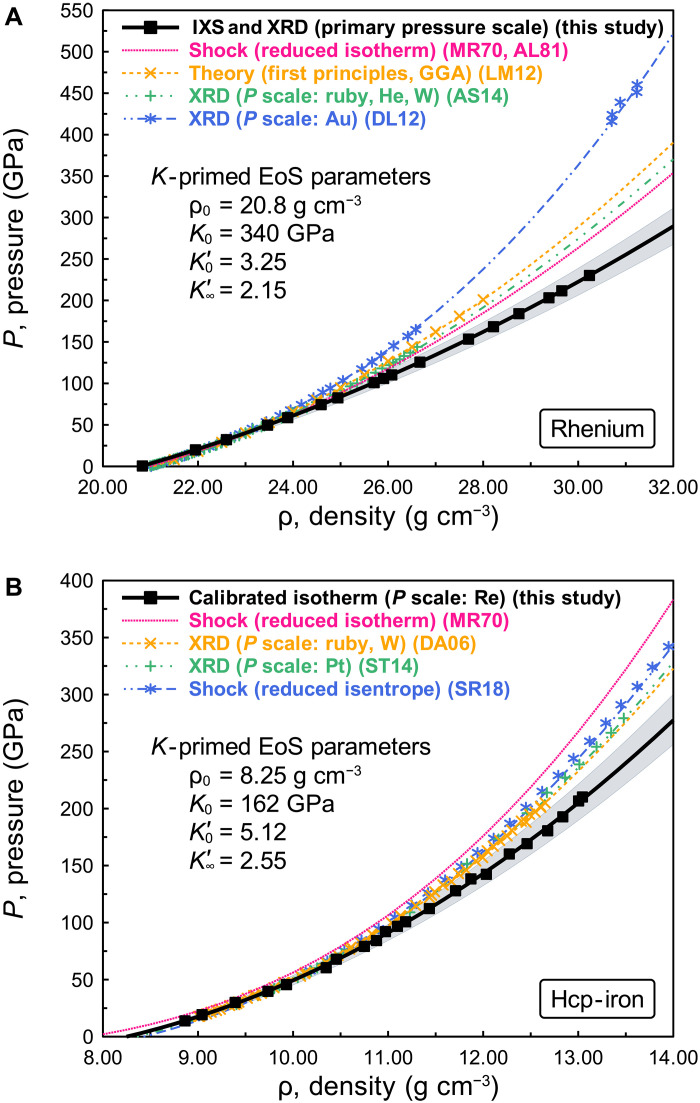
Primary pressure scale for rhenium and calibrated density-pressure relation for hcp-iron. (**A**) Primary pressure scale for rhenium. The black curve is the compression curve of our rhenium scale with the density determined experimentally and the pressure evaluated by our rhenium scale determined from density and acoustic velocities measured in runs IXS-Re-01 to IXS-Re-16 and IXS-Re-foil (Fig. 1B). (**B**) Calibrated density-pressure relation for hcp-iron. The black curve with black squares is the compression curve of hcp-iron with the present simultaneous compression experiment (table S4) calibrated by our rhenium scale (table S2). The shaded areas around the black curves represent the 1σ uncertainty of each curve. Other colored curves and symbols are the compression curves of rhenium (A) and hcp-iron (B) with experimental data based on pressure scales from previous studies [MR70 (*29*), AL81 (*30*), LM12 (*31*), AS14 (*25*), DL12 (*24*), DA06 (*5*), ST14 (*6*), and SR18 (*23*)].

The primary pressure scale can be derived from *v*_p_, *v*_s_, and ρ following the procedure of previous work (*15*, *16*, *19*, *20*). We used a *K*-primed EoS (*37*–*39*) to express the relation between density and pressure at multimegabar pressures. This EoS is based on the finite strain theory with the isothermal bulk modulus, *K*, and density, ρ, determined by the present IXS-XRD measurements. *K* and ρ are fitted with finite strain parameters of the bulk modulus at ambient pressure, *K*_0_, and its first pressure derivatives (∂*K*/∂*P*) at ambient pressure and infinite pressure, *K′*_0_ and *K′*_∞_, respectively. We used this EoS to keep consistency with the pressure dependence of thermodynamic Grüneisen parameter, γ_th_. Details are given in the “Primary pressure scale derivation” section in Methods. A good fit was found with ρ_0_ = 20.8(±0.1) g cm^−3^, *K*_0_ = 340(±9) GPa, *K′*_0_ = 3.25(±0.12), and *K′*_∞_ = 2.15(±0.11). The obtained EoS parameters and pressures for rhenium are given in tables S1 and S2. The uncertainty of the present pressure scale was evaluated by careful error propagation (notes S1 to S9), with the detailed discussion presented in note S10 and table S3. Figure 2A shows our primary pressure scale of rhenium, compared with previous pressure scales (*24*, *25*, *29*–*31*). Our rhenium scale and the previous pressure scales are reasonably consistent up to ~60 GPa (ρ ~ 24 g cm^−3^). However, differences are observed above 85 GPa (ρ ~ 25 g cm^−3^), and large differences, beyond the uncertainties, appear above 120 GPa (ρ ~ 26.5 g cm^−3^). The previous pressure scales give pressures 20% higher at ρ = 30.24 g cm^−3^, and the overestimation increases with increasing pressure. Our rhenium scale agrees with previous primary scales at lower pressures (*13*–*20*). Investigation shows the recent primary scale study is consistent with our scale, suggesting that previous secondary pressure scales overestimate pressures by 2 to 10% at 120 GPa (*21*). Comparing our scale with previous secondary scales, previous scales have overestimated the laboratory pressures by at least 20% at 230 GPa. The discrepancy of the rhenium scale and previous scales originates from the density dependence of the *v*_p_ of rhenium determined in this work (Fig. 1B). The experimental uncertainties derived from fitting the phonon dispersion, and *v*_p_ and *v*_s_ (fig. S6), preferred orientation and anisotropy (figs. S7 to S10), LS (fig. S11), density gradient (fig. S12), and diamond cupping (fig. S13) were evaluated in notes S4 to S10, fig. S14, and table S3. Even with the maximum uncertainty, there is still discrepancy of pressure values between present and previous scales (fig. S14).

### EoSs of rhenium, iron, and periclase

To understand the impact of our rhenium scale, we performed simultaneous compression experiments of rhenium, iron, and MgO by laser-annealing the samples to minimize the deviatoric stress (“Two-dimensional XRD measurement” section in Methods). Both the *c*/*a* ratios of rhenium and hcp-iron were consistent with those of calculated model *c*/*a* ratios (*31*, *36*) even at multimegabar pressures (fig. S15), which indicates that annealing of the sample well worked to minimize stress. We obtained *K*-primed EoS parameters of hcp-iron with ρ_0_ = 8.25(±0.05) g cm^−3^, *K*_0_ = 162(±5) GPa, *K′*_0_ = 5.12(±0.08), and *K′*_∞_ = 2.55(±0.09) and those of MgO with ρ_0_ = 3.58(±0.03) g cm^−3^, *K*_0_ = 159(±6) GPa, *K′*_0_ = 3.79(±0.08), and *K′*_∞_ = 2.29(±0.12) (“High-pressure and high-temperature EoSs for hcp-iron and MgO by the MGD model” and “Electronic contribution to heat capacity” sections in Methods and tables S2 and S4). Figure 2B and fig. S16 show the calibrated *K*-primed EoS of hcp-iron and MgO, respectively. The compression curve of hcp-iron based on our rhenium scale is consistent with the curves based on previous scales (*5*–*7*, *23*) up to 100 GPa within the uncertainties, but the differences become greater than 20% in the present maximum experimental pressure range. The compression curve of MgO based on our rhenium scale is consistent with the curves based on previous scales (*14*, *40*, *41*) in their respective experimental pressure ranges, within the uncertainties of our scale. The detailed comparison between the compression curves of MgO based on present and previous scales is given in note S11 and fig. S16.

**Fig. 3.**
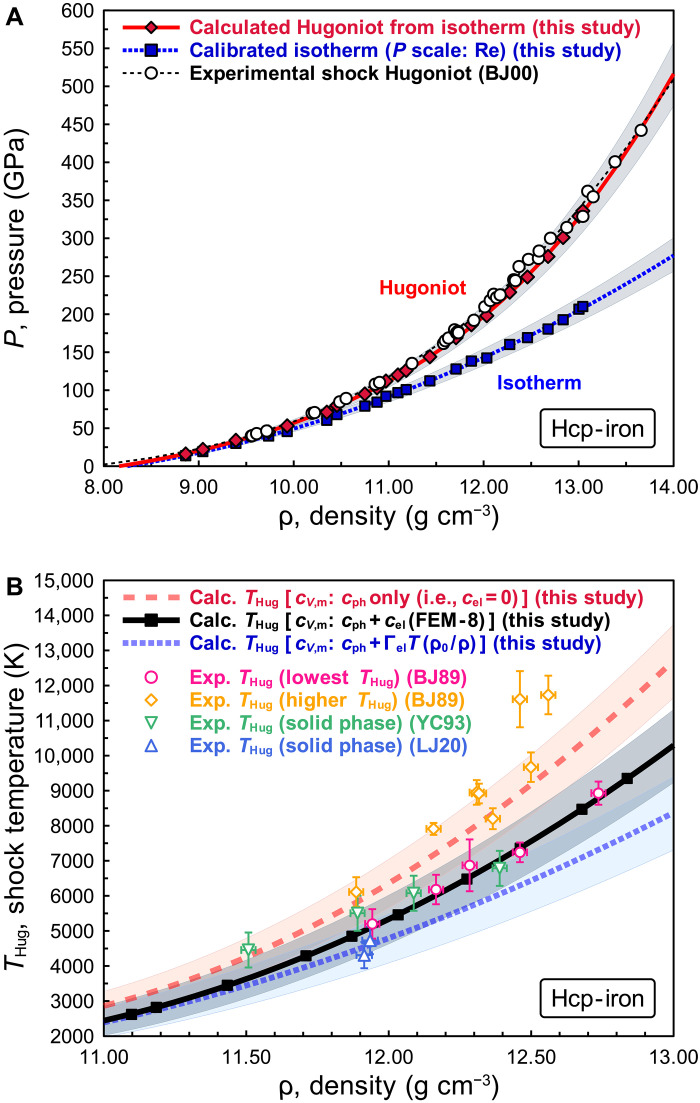
The isotherm of hcp-iron calibrated by the present rhenium scale, and calculated shock Hugoniot and its calculated shock temperature. (**A**) Isothermal compression curve and calculated shock Hugoniot of hcp-iron. The blue dotted curve with squares represents the isothermal compression curve of hcp-iron based on the present simultaneous compression experiment and our rhenium scale. The black dashed curve with open circles represents the Hugoniot curve with experimental shock compression data (BJ00) (*42*). The red solid curve with diamonds represents the calculated shock Hugoniot from the isotherm of hcp-iron based on the present simultaneous compression experiment and our rhenium scale (table S5). Our calculated shock Hugoniot can explain the experimental shock Hugoniot (*42*). (**B**) Comparison of calculated and experimental shock temperatures, *T*_Hug_, of hcp-iron based on three different molar heat capacity at constant volume, *c*_*V*,m_, models. The black squares are the calculated *T*_Hug_ with contributions of electrons to heat capacity, *c*_el_ by using the FEM-8 corresponding to the red diamonds in (A). The red dashed and blue dotted curves are the calculated *T*_Hug_ by the *c*_el_ = 0 model and the linear temperature dependence model [expressed as *c*_el_ = Γ_el_*T*(ρ_0_/ρ), where Γ_el_ is the electronic specific heat coefficient], respectively. The detailed description of the different approaches to determine *c*_*V*,m_ are presented in the “High-pressure and high-temperature EoSs for hcp-iron and MgO by the MGD model” and “Electronic contribution to heat capacity” sections in Methods and table S5. Other colored symbols are the experimentally measured *T*_Hug_ of solid iron from previous studies [BJ89 (*43*), YC93 (*44*), and LJ20 (*45*)]. The shaded areas around the curves in (A) and (B) represent the 1σ uncertainty of these curves.

Previous measurements using shock compression along the Hugoniot curve can be brought into agreement with our scale by careful consideration of the ρ dependence of the Grüneisen parameter. Because shock compression is not an isothermal process, thermal parameters are necessary to convert the Hugoniot curve to isotherms or vice versa, to compare the isothermal pressure scale and shock Hugoniot. The MGD model is widely used for high pressure and high temperature EoS, and the Grüneisen parameter, γ, and molar heat capacity at constant volume, *c*_*V*,m_, are critical as they are directly related to thermal pressure. Within the MGD model, the Grüneisen parameter represents the effect of crystal lattice volume change on its vibrational properties (*1*). Therefore, the Grüneisen parameter can be derived from the ρ dependence of *v*_p_ and *v*_s_. Especially for metals, *c*_*V*,m_ has contributions from both phonons and electrons. The detailed derivation of the *c*_*V*,m_ is given in the “Calculation of the shock Hugoniot from the isotherm” section in Methods (see also fig. S17 and table S5). We obtained MGD EoS parameters for rhenium with Θ = 369(±5) K, γ_0_ = 1.94(±0.31), γ_∞_ = (3 *K′*_∞_ − 1)/6 (fixed), *q* = 0.53(±0.30), parameters for hcp-iron with Θ = 515(±21) K, γ_0_ = 1.97(±0.16), γ_∞_ = (3 *K′*_∞_ − 1)/6 (fixed), and *q* = 0.37(±0.24), and parameters for MgO with Θ = 760(±135) K, γ_0_ = 1.53(±0.26), γ_∞_ = (3 *K′*_∞_ − 1)/6 (fixed), and *q* = 0.44(±0.68), where Θ is the Debye temperature, γ_0_ and γ_∞_ are the Grüneisen parameters at ambient and infinite pressures, respectively, and *q* gives its ρ dependence (“High-pressure and high-temperature EoSs for hcp-iron and MgO by the MGD model” and “Electronic contribution to heat capacity” sections in Methods and tables S2). Figure 3A shows simultaneous compression data of hcp-iron calibrated by our rhenium scale and calculated shock Hugoniot compared with experimental shock compression data (*42*). We reproduced the Hugoniot curve of iron based on our EoS of hcp-iron with the Grüneisen parameter determined by the isothermal bulk modulus from this work and Birch’s law of hcp-iron in (*8*) as shown in Fig. 3A and fig. S18A. Detailed derivation of the calculated shock Hugoniot is given in the “Calculation of the shock Hugoniot from the isotherm” section in Methods and table S5. These figures show that our calculated shock Hugoniot is nicely consistent with experimental shock compression data (*42*). Figure S18B shows our Grüneisen parameter, derived from Birch’s law of hcp-iron in (*8*) with our EoS compared with the Grüneisen parameter used for the previous reference EoS (*5*). This figure indicates that our EoS is consistent with both shock compression data and the experimentally determined *v*_p_ of hcp-iron (*8*), whereas the Grüneisen parameter in the previous reference EoS (*5*) is inconsistent with *v*_p_ of hcp-iron. This provides additional strong evidence in favor of the present pressure scale.

Figure 3B shows the calculated shock temperature, *T*_Hug_, from our EoS compared with the experimental *T*_Hug_ of solid iron (*43*–*45*). Because of the difficulty in measuring *T*_Hug_, and/or the solid-liquid transition effect, or superheating state of iron over the melting curve [e.g., (*46*)], experimental estimates of *T*_Hug_ show large variations (e.g., ~4000 to 6500 K around ρ ~ 12 g cm^−3^ and ~6000 to 12,000 K around ρ ~ 12.5 g cm^−3^) in previous measurements and remain under debate. However, as shown in Fig. 3B, our calculated *T*_Hug_ by a free electron model with eight valence electrons (FEM-8) is consistent with most of experimental *T*_Hug_ of solid iron (*43*–*45*) within uncertainties (note S12 and fig. S17C). The shock Hugoniot of rhenium and MgO also can be reproduced from each isotherm based on our rhenium scale within the uncertainties (figs. S19 and S20). Our calculated *T*_Hug_ of MgO is also consistent with the experimental *T*_Hug_ of MgO (*47*–*50*) within the uncertainties (fig. S20B).

## DISCUSSION

**Fig. 4.**
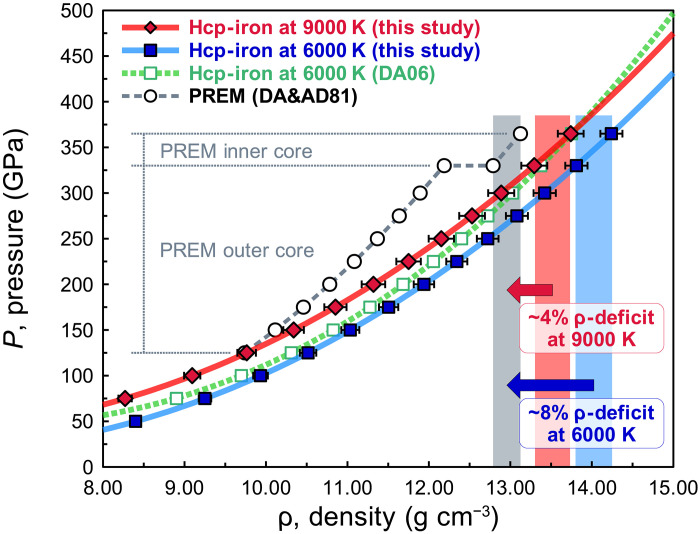
Density-pressure relations of hcp-iron at high temperature and PREM. The red and blue curves with symbols are compression curves of hcp-iron at 9000 and 6000 K with 1σ error bars of density determined from the present work. The green dotted curve with open square symbols is the compression curve of hcp-iron at 6000 K determined by a previous pressure scale (DA06) (*5*). The gray dashed curve with open circles represents the density-pressure relation of the PREM (DA&AD81) (*9*). We used the heat capacity model by using the Debye model (DM) and the FEM-8 in this calculation (“High-pressure and high-temperature EoSs for hcp-iron and MgO by the MGD model” and “Electronic contribution to heat capacity” sections in Methods). Details are given in table S6. The red and blue arrows indicate the density deficits between hcp-iron and PREM for the compression curves of 9000 and 6000 K, respectively.

Our revised pressure scale has important implications in the context of the seismically determined compositional model of Earth’s interior, the preliminary reference Earth model (PREM) (*9*). Previously, a ~3 to 5% density deficit compared to hcp-iron was estimated for Earth’s inner core (*5*, *6*, *10*). Figure 4 shows the density deficits of the PREM inner core from hcp-iron at high pressure and high temperature. We used our thermal EoS of hcp-iron to model the iron density at Earth’s inner core conditions in Fig. 4. We used the *K*-primed EoS, the MGD model, and the FEM-8 for the *c*_*V*,m_ of hcp-iron. The details of the procedure to derive the high-pressure and high-temperature EoS for hcp-iron are given in the “High-pressure and high-temperature EoSs for hcp-iron and MgO by the MGD model” and “Electronic contribution to heat capacity” sections in Methods, figs. S17 and S18, and tables S2 and S7 to S10.

In the range of 330 to 365 GPa and 6000 K, typical for estimations of Earth’s inner core conditions (*10*–*12*), the density deficit from hcp-iron via our rhenium scale is 8(±2)%, which is much larger than ~3 to 5% of the previously estimated density deficit. The detailed analyses of the density deficit of Earth’s inner core are also given in the “Primary pressure scale derivation” to “Electronic contribution to heat capacity” sections in Methods, fig. S17D, and table S6. If the density deficit is constrained to ~3 to 5% as previously estimated, a much higher temperature around 9000 K is required [~3000 K higher than the previous estimate (*10*–*12*)]. In conclusion, to account for the density of the PREM inner core, our present rhenium scale requires approximately a factor of two more light elements in Earth’s inner core than previously estimated, or much higher core temperatures, or some combination thereof as shown in Fig. 4.

## METHODS

### Starting material and high-pressure generation

We performed the compression experiments both using direct compression without pressure medium and quasi-hydrostatic compression with a magnesium oxide (periclase, MgO) pressure medium for acoustic velocity measurements. For the experiments without pressure medium, we used a DAC with double-beveled diamond anvils with a culet of 30 μm in diameter. A rhenium metal foil (250 μm in thickness, 99.97% purity, Alfa Aesar) was precompressed to a thickness of about 20 to 30 μm and used as the starting material. We increased pressure in 12 compressional steps (IXS-Re-01 to IXS-Re-12). For the experiments with the MgO pressure medium, we used a DAC with single-beveled diamond anvils with a culet of 150 μm in diameter. A rhenium powder (−325 mesh, 99.99% purity, Alfa Aesar) was used as a starting material. The rhenium powder was precompressed to a thickness of about 15 μm and was surrounded by the MgO (>98% purity, Junsei Chemical) pellets of about 5-μm thickness, which served as the pressure medium. The sample and pressure medium were loaded into a sample hole with a diameter of about 50 μm of a precompressed rhenium gasket foil (250 μm in thickness, 99.97% purity, Alfa Aesar), which was about 25 μm in thickness. We increased pressure in four compressional steps (IXS-Re-13 to IXS-Re-16), and the sample was annealed at temperature over 1000 K after each compression (“IXS measurement” section). The acoustic velocity measurement of rhenium at ambient conditions (IXS-Re-foil, in air) were also performed using a rhenium metal foil (25 μm in thickness, Nilaco), which was precompressed to a thickness of about 10 to 15 μm.

We performed the simultaneous compression experiment of rhenium, iron, and MgO to establish the EoSs of iron and MgO based on our rhenium scale. We used a DAC with double-beveled diamond anvils with a culet of 50 μm and a rhenium gasket (250 μm in thickness, 99.97% purity, Alfa Aesar). A rhenium powder (−325 mesh, 99.99% purity, Alfa Aesar) and an iron powder (99.99% purity, Wako Chemicals) were mixed as a starting material and precompressed to a thickness of about 15 μm. The mixture was surrounded by the MgO (>98% purity, Junsei Chemical) pellets of about 5-μm thickness, which served as the pressure medium. The sample and pressure medium were loaded into a sample hole (with a diameter of about 15 μm) in a precompressed rhenium gasket foil (with a thickness of 25 μm).

### IXS measurement

The acoustic velocity of rhenium was measured by IXS at BL43LXU (*27*) of the RIKEN SPring-8 Center. The Si (9 9 9) reflection at 17.79 keV provided a resolution of 2.8 meV (IXS-Re-01 to IXS-Re-16, high-pressure experiments) and the Si (11 11 11) reflection at 21.75 keV provided a resolution of 1.3 meV (IXS-Re-foil, in-air). The x-ray beam size for the high-pressure runs was focused to 5 μm by 5 μm at 17.79 keV by a multilayer Kirkpatrick-Baez mirror pair (*28*). The x-ray beam with 50 μm by 50 μm at 21.75 keV was used for the measurements under the ambient conditions (in air). To reduce the scattering background from the diamonds and improve the signal-to-noise ratio, a Soller screen (*28*) was installed downstream of the DAC at pressures over 150 GPa (IXS-Re-07 to IXS-Re-12) without pressure medium and also for all runs with the MgO pressure medium (IXS-Re-13 to IXS-Re-16). In runs IXS-Re-13 to IXS-Re-16, the rhenium sample with the MgO pressure medium was annealed at temperatures over 1000 K after each compression by a double-sided laser-heating method using a fiber laser installed at BL43LXU (COMPAT system) (*51*) and quenched to ambient temperature before the IXS measurements, to minimize the deviatoric stress. A Soller screen was not used in the experiments in air without a DAC (IXS-Re-foil). The IXS spectra at several momentum transfers were collected simultaneously by 23 (12, with the Soller screen) analyzers, arranged in a two-dimensional 4 × 6 array in runs from IXS-Re-01 to IXS-Re-12, and 28 (16, with the Soller screen) analyzers, arranged in a two-dimensional 4 × 7 array in runs from IXS-Re-13 to IXS-Re-16 and IXS-Re-foil. The IXS spectra were collected for about 8 to 24 hours in each experimental run. The TA mode did not appear at some pressures (see fig. S1) because the measurement time was limited for those cases and thus the data quality was insufficient to clearly isolate the TA mode in the spectra. The TA peaks (Fig. 1A and fig. S2A) were larger than expected from calculations of perfect single crystals (fig. S3). We discuss this and conclude that it is from a quite high (~0.1 to 1 nm^−2^) defect density that appears after rhenium is pressurized (fig. S4) in note S2.

### Phonon dispersion and fitting

The IXS spectra are characterized by elastic scattering near zero energy and inelastic contributions from the LA and TA modes of rhenium and diamond (Fig. 1A and fig. S2A). The energy positions of the inelastic contribution were extracted by fitting with Lorentzian functions. The relation between the excitation energy, the velocity for each acoustic mode, and the momentum transfer of phonons is given by

$$E=\frac{{hvQ}_{\max}}{\pi^{2}}\sin\left(\frac{\pi Q}{2Q_{\max}}\right)$$ (2)

where *E* is excitation energy, *h* is Plank constant, *v* is acoustic velocity for each LA and TA mode, *Q* is momentum transfer, and *Q*_max_ is the averaged distance to the edge of the first Brillouin zone (BZ) including the effect of the preferred orientation. To obtain the acoustic velocity from the IXS results, the dispersion was fit with the sine function, Eq. 2, which was used to determine the long-wavelength (the limit as *Q* approaches zero) acoustic velocity (*8*, *52*, *53*). A weighted least-squares method was used with *v* and *Q*_max_ as free parameters (see also note S4). Figures S1 and S2B show the fitting results of the LA and TA phonon dispersions for rhenium. LA phonons were clear in all 16 high-pressure experimental conditions and 1 ambient condition. In runs IXS-Re-05, 06, and 11, the TA phonons could not be clearly identified because of poor signal-to-noise ratio and/or insufficient exposure time (within the limitation of the experimental beamtime). However, TA peaks were observed in some of the spectra in these runs, and they were found to be consistent with the estimated TA phonon dispersion curves (gray dashed lines in fig. S1) calculated by Eq. 2 with interpolated *v* (red dotted line in fig. S5B) and *Q*_max_ (averaged distance to the edge of the first BZ in fig. S6A) values.

### Two-dimensional XRD measurement

Two-dimensional XRD patterns were taken to measure the density and to characterize the texture of the sample and were done in the same optical setup at BL43LXU (in situ) using a flat-panel detector (FP, C9732DK, Hamamatsu Photonics). The XRD was measured in situ, with the same incident x-ray beam at the same position on the sample as for the IXS work. This allowed us to investigate the impact of hydrostatic/nonhydrostatic conditions, preferred orientation, and LSs on the same sample volume used to measure the acoustic velocities (notes S3 to S9 and figs. S3 to S13). The distance between the sample and the flat-panel detector was calibrated by using a cerium dioxide standard (National Institute of Standards and Technology). The lattice parameters and densities, ρ, of rhenium in the hcp structure were obtained from XRD patterns as

$$\frac{1}{d_{\left(hkl\right)}^{2}}=\frac{4}{3}\left(\frac{h^{2}+hk+k^{2}}{a^{2}}\right)+\frac{l^{2}}{c^{2}}$$ (3)

$$\rho =\frac{ZM}{N_{A}}\frac{2}{\sqrt{3}a^{2}c}$$ (4)

where *a* and *c* are lattice parameters, *h*, *k*, and *l* are Miller indices, *d*_(*hkl*)_ is the *d*-spacing value for a reflection indexed by *hkl*, *Z* is the number of atoms in the rhenium lattice, *M* is molar mass of rhenium, and *N*_A_ is Avogadro constant. The density determination was carried out by using six *d*-spacing values of different diffraction peaks (*hkl*: 100, 002, 101, 102, 110, and 103) as shown in fig. S7. The XRD patterns were analyzed using the IPAnalyzer/PDindexer/ReciPro software package (*54*, *55*).

For the simultaneous compression experiments of rhenium, iron, and MgO, we performed annealing the samples to measure reasonable density relations among rhenium, iron, and MgO for minimizing the deviatoric stress in the samples. The samples were annealed at temperatures over 1000 K after each compression by a double-sided laser-heating method using a fiber laser installed at BL43LXU (COMPAT system) (*51*) and quenched to ambient temperature before taking XRD patterns. The lattice parameters and densities of rhenium, iron, and MgO were determined by XRD patterns (table S4).

### Primary pressure scale derivation

The primary pressure scale can be derived from compressional and shear wave velocities and density (*v*_p_, *v*_s_, and ρ) following the procedure in previous work of polycrystalline samples (*15*, *16*, *19*, *20*). We used the *K*-primed EoS to keep consistency of the pressure dependence of thermodynamic Grüneisen parameter, γ_th_, i.e., it must be greater than two-thirds because of the thermodynamic consistency, whereas other EoSs including Birch-Murnaghan EoS and Rydberg-Vinet EoS violate the consistency at extremely high pressures (*38*, *39*). The isothermal bulk modulus, *K*, and the density, ρ, were fit with the *K*-primed EoS (*37*–*39*) as follows

$$P=K_{0}\left\{\frac{K_{0}^{\prime }}{K_{\infty }^{\prime 2}}\left[{\left(\frac{\rho }{\rho_{0}}\right)}^{K_{\infty }^{\prime }}-1\right]-\left(\frac{K_{0}^{\prime }}{K_{\infty }^{\prime }}-1\right)\ln\left(\frac{\rho }{\rho_{0}}\right)\right\}$$ (5)

$$K=K_{0}\left\{\frac{K_{0}^{\prime }}{K_{\infty }^{\prime }}\left[{\left(\frac{\rho }{\rho_{0}}\right)}^{K_{\infty }^{\prime }}-1\right]+1\right\}$$ (6)

$$\gamma_{\infty }=\frac{3K_{\infty }^{\prime }-1}{6}$$ (7)

where *K*_0_ is the isothermal bulk modulus at ambient pressure and *K′*_0_ and *K′*_∞_ are its first pressure derivatives (∂*K*/∂*P*) at ambient and infinite pressures, respectively.

*v*_p_ and *v*_s_ are related to the adiabatic bulk modulus, *K_S_*, and the shear modulus, *G*, as follows [the formulas used here can be found in (*1*)]

$$K_{S}=\rho \left(v_{p}^{2}-\frac{4}{3}v_{s}^{2}\right)$$ (8)

$$G=\rho v_{s}^{2}$$ (9)

The isothermal bulk modulus, *K*, is related to the adiabatic bulk modulus, *K_S_*, the thermodynamic Grüneisen parameter, γ_th_, the molar heat capacity at constant volume, *c*_*V*,m_, density, ρ, molar mass, *M*, and temperature, *T*

$$K=K_{S}-\gamma_{\mathrm{th}}^{2}\frac{\rho c_{V,m}}{M}T$$ (10)

In a Debye model (DM), the Debye velocity *v*_D_ is defined

$$\frac{3}{v_{D}^{3}}=\frac{1}{v_{p}^{3}}+\frac{2}{v_{s}^{3}}$$ (11)

Debye temperature Θ is defined

$$\text{Θ}=\frac{hv_{D}}{2k_{B}}{\left(\frac{6N_{A}\rho }{\pi M}\right)}^{\frac{1}{3}}$$ (12)

where *h* is Plank constant, *k*_B_ is Boltzman constant, *N*_A_ is Avogadro’s constant, and *M* is the molar mass.

There are several definitions of the Grüneisen parameter. Under the quasi-harmonic approximation, thermodynamic (macroscopic) Grüneisen parameter, γ_th_, is defined

$$\gamma_{\mathrm{th}}=\frac{M}{\rho c_{V,m}}{\left(\frac{\partial P}{\partial T}\right)}_{V}$$ (13)

Debye-Grüneisen (microscopic) parameter, γ_D_, can be expressed by the Debye temperature, which is related to the vibration energy by Eq. 12

$$\gamma_{D}=\frac{\partial \ln(\text{Θ})}{\partial \ln(\rho )}$$ (14)

In the Debye approximation, the macroscopic and microscopic thermodynamic properties are assumed to be the same, thus

$$\gamma_{\mathrm{th}}=\gamma_{D}$$ (15)

On the other hand, the temperature dependence of the γ_th_ can be expressed as (*39*)

$$\frac{\partial \gamma_{\mathrm{th}}}{\partial T}=-\frac{1}{T}{\left[\frac{\partial \ln(c_{V,m})}{\partial \ln(\rho )}\right]}_{S}$$ (16)

which indicates the temperature dependence of the γ_th_ is inversely proportional to the temperature. In addition, under the Debye approximation, *c*_*V*,m_ becomes almost equal to constant of 3*nR* above the Debye temperature (the Dulong-Petit law). Thus, the temperature dependence of the γ_th_ can be negligible.

The density dependence of the Grüneisen parameter γ_th_ is expressed as a function of density with negligible temperature dependence by Al’tshuler form (*56*) as

$$\gamma =\gamma_{\infty }+(\gamma_{0}-\gamma_{\infty }){\left(\frac{\rho_{0}}{\rho }\right)}^{q}$$ (17)

where γ_∞_ is the Grüneisen parameter at infinite pressure and *q* gives the ρ dependence. In Al’tshuler form, either γ_∞_ or *q* was usually fixed. In this study, we fixed γ_∞_ as (3 *K′*_∞_ − 1)/6, which is a theoretical constraint of Grüneisen parameter in the *K*-primed EoS (*37*–*39*). The ρ dependence of the Debye temperature can be expressed from Eqs. 14 and 17 as

$$\text{Θ}={\text{Θ}}_{0}{\left(\frac{\rho_{0}}{\rho }\right)}^{-\gamma_{\infty }}\exp\left\{\frac{\gamma_{0}-\gamma_{\infty }}{q}\left[1-{\left(\frac{\rho_{0}}{\rho }\right)}^{q}\right]\right\}$$ (18)

The parameters γ_0_, γ_∞_, and *q* for thermodynamic Grüneisen parameter and Θ_0_ for Debye temperature can be derived by fitting with the experimental dataset of *v*_p_, *v*_s_, and ρ from Eqs. 11, 12, and 18.

*c*_*V*,m_ is assumed to be a sum of contributions from phonons (*c*_ph_) and electrons (*c*_el_) (*57*). However, *c*_el_ is assumed to be zero at ambient temperature, because the contribution by electrons is negligible compared to phonons at low temperature (e.g., *T* < Θ). *c*_ph_ is derived using the DM

$$c_{\mathrm{ph}}=9nR{\left(\frac{T}{\text{Θ}}\right)}^{3}\int_{0}^{\text{Θ}/T}\frac{x^{4}\exp(x)}{{\left[\exp(x)-1\right]}^{2}}dx$$ (19)

where *n* is the number of atoms per chemical formula unit, *R* is the gas constant, *T* is the temperature, and Θ is the Debye temperature expressed by Eq. 18. By using Eqs. 10, 16, and 19, the isothermal bulk modulus, *K*, can be derived from the dataset of *v*_p_, *v*_s_, and ρ determined experimentally. Last, we can determine the parameters for the *K*-prime EoS, *K′*_0_ and *K′*_∞_ with the fixed *K′*_∞_ from Eq. 7, by fitting *K* and ρ to Eq. 6 of the *K*-primed EoS. The parameters of *K*-primed EoS for rhenium are given in table S2.

### High-pressure and high-temperature EoSs for hcp-iron and MgO by the MGD model

We re-evaluated the EoSs of hcp-iron and MgO at high pressure and ambient temperature using the present rhenium pressure scale (table S2) with the *K*-primed EoS based on our simultaneous compressional experiments of rhenium, iron, and MgO.

The parameters for the *K*-primed EoS at ambient temperature, ρ_0_, *K*_0_, *K′*_0_, and *K′*_∞_, are derived from Eq. 5 using the measured densities and our rhenium scale (table S4). To do this, the Grüneisen parameter, γ_∞_ was taken from the relation of the *K*-primed EoS, γ_∞_ = (3 *K′*_∞_ − 1)/6. However, because *v*_s_ for hcp-iron and *v*_p_ for MgO at sufficiently high pressure conditions are not available, we performed following procedures to obtain the Grüneisen parameter using *v*_p_ for hcp-iron and *v*_s_ for MgO, together with our EoSs of hcp-iron and MgO determined in this study. Under ambient temperature, the differences between *K* and *K_S_* are not large (e.g., the differences for rhenium in this study are less than 1%). Thus, if we assume that isothermal and adiabatic bulk moduli are equal (*K* = *K_S_*) at ambient temperature, we could derive provisional *v*_s_ of hcp-iron and *v*_p_ of MgO from Eqs. 8 and 9 by using reference data of *v*_p_ for hcp-iron (*8*) and *v*_s_ for MgO (*58*) combined with our EoSs of hcp-iron and MgO, respectively. Here, we can derive the provisional values of Grüneisen parameter and Debye temperature from the Eqs. 17 and 18 as was derived for rhenium. By using those provisional parameters of Debye temperature and Grüneisen parameter with Eq. 10, we can derive the provisional isothermal bulk modulus *K*. Using this isothermal bulk modulus *K*, we derive updated values for *v*_s_ of hcp-iron (and *v*_p_ of MgO) and updated values for the Grüneisen parameter, γ_D_, and the Debye temperature, Θ. After several iterations, the isothermal bulk modulus, *K*, Grüneisen parameter, γ_D_, and Debye temperature, Θ, converge, giving a self-consistent set of values for hcp-iron and MgO shown in table S2. The Grüneisen parameter of hcp-iron determined by the *K* = *K_S_* assumption and the converged result after iteration are shown in fig. S18B. The difference between two values for the Grüneisen parameter is about 1%, consistent within the uncertainty of the parameter.

The thermal pressure of hcp-iron under high-pressure and high-temperature conditions are derived from the present *K*-primed EoS and the γ_th_ of hcp-iron with the MGD model. The pressure at high-temperature conditions is derived from the isothermal pressure at ambient conditions with the thermal pressure *P*_th_ as

$$P_{\left(\rho ,T\right)}=P_{\left(\rho ,300\;K\right)}+P_{\mathrm{th}(\rho ,T)}-P_{\mathrm{th}(\rho ,300\;K)}$$ (20)

where the thermal pressure *P*_th_ is derived from the quasi-harmonic Debye thermal pressure

$$P_{\mathrm{th}(\rho ,T)}=\gamma_{\mathrm{th}}\frac{\rho }{M}\int_{0}^{T}c_{V,m(\rho ,T)}dT$$ (21)

*c*_*V*,m_ is assumed to be a sum of contributions from phonons and electrons (*57*) as

$$c_{V,m(\rho ,T)}=c_{\mathrm{ph}(\rho ,T)}+c_{\mathrm{el}(\rho ,T)}$$ (22)

As described in the “Primary pressure scale derivation” section, the γ_th_ and the Debye temperature, Θ, can be derived from *v*_p_, *v*_s_, and ρ with Eqs. 11, 12, and 18, and *c*_ph_ is derived by Eq. 19. We used the FEM-8 to the *c*_*V*,m_ of hcp-iron for calculation of thermal pressure, *P*_th_. The details of the electron contribution to the *c*_*V*,m_ are given in the “Electronic contribution to heat capacity” section, and the details of the parameters of high-pressure and high-temperature EoS for hcp-iron are given in tables S7 to S10.

### Electronic contribution to heat capacity

The electronic contribution to the heat capacity is generally negligible compared to the phonon contribution at low temperatures, but it increases at higher temperature. It is important in the present context as we compare out results to shock Hugoniot done at high temperature. In particular, we consider a linear temperature dependence model (LTD) and the FEM.

The electronic contribution, *c*_el_, may be expressed as a linear temperature relation by the electronic specific heat coefficient, Γ_el_, combined with the density dependence [e.g., Γ_el_ of rhenium is 2.29 mJ K^−2^ mol^−1^, and Γ_el_ of iron is 4.90 mJ K^−2^ mol^−1^ (*59*), obtained from resistivity measurements at near absolute zero temperature]. Using the LTD, *c*_el_ becomes comparable with *c*_ph_ at density, ρ ~ 12.8 g cm^−3^, and shock temperature, *T*_Hug_ ~ 10,000 K on the Hugoniot curve, doubling the total heat capacity as shown in fig. S17B. Therefore, the MGD-EoS (i.e., also shock temperature estimation), especially at high temperature, depends sensitively on how *c*_el_ is estimated. On the other hand, recent experimental and theoretical studies of the resistivity for iron at high pressure and high temperature suggest that the resistivity of iron may be about one-half to one-third of previous estimates [e.g., (*60*–*62*)]. In addition, it has been experimentally confirmed that there is a strong correlation between the temperature derivative of resistivity and heat capacity of iron [e.g., (*63*)]. Thus, the recent low resistivity results may suggest that the actual electronic contributions to *c*_*V*,m_ is lower than that estimated by the LTD. For example, Brown and McQueen (*64*) used a simplified model, FEM, to consider the electron contributions of iron theoretically and showed that the FEM proposed lower electrical heat capacity (although the FEM has been considered that the precise electron behavior cannot be estimated for transition metals, such as iron). In this work, we compare three different models of *c*_*V*,m_, which is a sum of *c*_ph_ derived by the DM and the *c*_el_ models of *c*_el_ = 0, LTD, and FEM, as follows

$$c_{V,m,\mathrm{DM}-\mathrm{zero}}=c_{\mathrm{ph},\mathrm{DM}}$$ (23)

$$c_{V,m,\mathrm{DM}-\mathrm{LTD}}=c_{\mathrm{ph},\mathrm{DM}}+{\text{Γ}}_{\mathrm{el}}T\left(\frac{\rho_{0}}{\rho }\right)$$ (24)

$$c_{V,m,\mathrm{DM}-\mathrm{FEM}}=c_{\mathrm{ph},\mathrm{DM}}+c_{\mathrm{el},\mathrm{FEM}}$$ (25)

Deriving *c*_el,FEM_ by using the FEM, the probability, *f*_el_, that an energy level, ɛ, is occupied by electrons at a temperature, *T*, is expressed by the Fermi-Dirac distribution

$$f_{\mathrm{el}(\varepsilon ,\rho ,T)}={\left\{\exp\left[\frac{\varepsilon -\mu_{\left(\rho ,T\right)}}{k_{B}T}\right]+1\right\}}^{-1}$$ (26)

where *k*_B_ is the Boltzmann constant and μ_(ρ,*T*)_ is the chemical potential at density, ρ, and *T*. Under free electron approximation, which assumes that the valence electrons move freely among the atoms, the molar density of electron states, *D*_el_, can be expressed as

$$D_{\mathrm{el}(\varepsilon ,\rho )}=\frac{8\pi M}{h^{3}\rho }\sqrt{2m_{e}^{3}\varepsilon }$$ (27)

where *h* is Plank constant, *M* is the molar mass, and *m*_e_ is the electron mass. The number of valence electrons per mole can be obtained by integrating the product of density of state, *D*_el_, and effect of temperature, *f*_el_, with respect to quasi continuum of energies, ɛ, as:

$$N_{A}n_{\mathrm{el}}=\int_{-\infty }^{+\infty }D_{\mathrm{el}(\varepsilon ,\rho )}f_{\mathrm{el}(\varepsilon ,\rho ,T)}d\varepsilon$$ (28)

where *N*_A_ is Avogadro constant and *n*_el_ is the valence electrons in an atom [e.g., for iron, *n*_el_ = 8 (4s^2^ 3d^6^)]. Because *n*_el_ is independent of ρ and *T*, the chemical potential μ_(ρ,*T*)_ can be obtained by numerically analyzing Eqs. 26 to 28 [e.g., (*57*)]. Here, the total electronic contribution to the internal energy and the heat capacity by the FEM can be obtained as

$$E_{\mathrm{el},\mathrm{FEM}(\rho ,T)}=\int_{-\infty }^{+\infty }\varepsilon D_{\mathrm{el}(\varepsilon ,\rho )}f_{\mathrm{el}(\varepsilon ,\rho ,T)}d\varepsilon$$ (29)

$$c_{\mathrm{el},\mathrm{FEM}(\rho ,T)}={\left[\frac{\partial E_{\mathrm{el},\mathrm{FEM}(\rho ,T)}}{\partial T}\right]}_{\rho }$$ (30)

The differences among *c*_el_ models have only small impact on the shock pressure, *P*_Hug_, but have large impact on *c*_*V*,m_ and the shock temperature, *T*_Hug_ (see also note S12 and figs. S17 and S20). Thus, *T*_Hug_ can be used to evaluate the validity of the Grüneisen parameter, its ρ dependence, and *c*_el_ model. We compare the calculated *T*_Hug_ of iron (Fig. 3B) and MgO (fig. S20B) to the experimentally available values for each material. The experimentally measured *T*_Hug_ (*43*–*45*) of hcp-iron is better explained by using the FEM-8 model for *c*_el_ (Fig. 3B). Because MgO is an insulator, the calculated *T*_Hug_ of MgO (fig. S20B) derived assuming *c*_el_ = 0 by Eq. 23 is also reasonable agreement with the experimentally measured *T*_Hug_ (*47*–*50*). The details of the derivation of calculated *P*_Hug_ and *T*_Hug_ are given in the “Calculation of the shock Hugoniot from the isotherm” section.

### Calculation of the shock Hugoniot from the isotherm

Under shock compression, the conditions of the system can be derived by Rankine-Hugoniot equations as follows (*1*)

$$\rho_{\mathrm{Hug}}=\rho_{\mathrm{init}}\frac{U_{s}}{U_{s}-U_{p}}$$ (31)

$$P_{\mathrm{Hug}}=\rho_{\mathrm{init}}U_{s}U_{p}$$ (32)

$$\text{Δ}E_{\mathrm{Hug}}=-\frac{1}{2}P_{\mathrm{Hug}}\left(\frac{M}{\rho_{\mathrm{Hug}}}-\frac{M}{\rho_{\mathrm{init}}}\right)=\frac{1}{2}{MU}_{p}^{2}$$ (33)

where ρ_init_ is the density before shock compression; ρ_Hug_, *P*_Hug_, Δ*E*_Hug_ are the density, pressure, and the increase of internal energy after shock compression; *U*_s_ and *U*_p_ are the shock and particle velocities; and *M* is the molar mass. A reversible path is necessary to estimate the shock energy deposited and therefore the shock temperature, *T*_Hug_. The total increase of internal energy by shock compression is equal to the increase in the following adiabatic and isochoric processes

$$\text{Δ}E_{\mathrm{Hug}}=\text{Δ}E_{S}+\text{Δ}E_{V}$$ (34)

where Δ*E_S_* is the increase of internal energy under the adiabatic compression from the molar volume at initial conditions, *V*_m,init_, to the molar volume after shock compression, *V*_m,Hug_, and Δ*E_V_* is the increase of internal energy by the isochoric temperature increase from the temperature after the adiabatic compression, *T_S_*, to the shock temperature, *T*_Hug_. In the adiabatic process, the Δ*E_S_* can be derived as follows (*65*)

$$\text{Δ}E_{S}=-{\left(\int_{V_{m,\mathrm{init}}}^{V_{m,\mathrm{Hug}}}P_{S}dV_{m}\right)}_{S}$$ (35)

In an adiabatic process, the temperature changes while the entropy is constant giving

$$TdS=c_{V,m}dT+T{\left(\frac{\partial P}{\partial T}\right)}_{V}dV_{m}=0$$ (36)

*T_S_* can be derived by integrating Eq. 36 using Eq. 16 as follows

$$T_{S}=T_{\mathrm{init}}\exp\left(-\int_{V_{m,\mathrm{init}}}^{V_{m,\mathrm{Hug}}}\frac{\gamma_{\mathrm{th}}}{V_{m}}dV_{m}\right)$$ (37)

In the isochoric process, the Δ*E_V_* can be derived as follows

$$\text{Δ}E_{V}=\int_{T_{S}}^{T_{\mathrm{Hug}}}c_{V,m}dT$$ (38)

The shock temperature, *T*_Hug_, can be estimated using Eqs. 31 to 38. Thus, the calculated shock Hugoniot from the isotherm, or vice versa, the reduced isotherm from the shock Hugoniot, can be derived by the thermal pressure with the shock temperature and the MGD model.

## Supplementary Materials

### This PDF file includes:

Supplementary Note Figs. S1 to S21 Tables S1 to S10 References
