# Supplementary material for: Density deficit of Earth’s core revealed by a multimegabar primary pressure scale
Source: Sci Adv. 2023 Sep 8;9(36):eadh8706. doi: 10.1126/sciadv.adh8706 (PMC10491286; doi:10.1126/sciadv.adh8706)
Supplement: Supplementary file 1 — Supplementary Note Figs. S1 to S21 Tables S1 to S10 References [file sciadv.adh8706_sm.pdf]

Science Advances
AAAS

Supplementary Materials for

Density deficit of Earth's core revealed by a multimegabar primary pressure scale

Daijo Ikuta *et al.*

Corresponding author: Daijo Ikuta, dikuta@okayama-u.ac.jp; Eiji Ohtani, eohtani@tohoku.ac.jp; Alfred Q. R. Baron, baron@spring8.or.jp

Sci. Adv. **9**, eadh8706 (2023)
DOI: 10.1126/sciadv.adh8706

This PDF file includes:

Supplementary Note
Figs. S1 to S21
Tables S1 to S10
References

Supplementary Note

S1. Comparison of velocities between inelastic x-ray scattering and ultrasonic methods

To evaluate the validity of high-pressure experimental acoustic velocities (v_p and v_s) obtained from phonon dispersion by inelastic x-ray scattering (IXS) method (Fig. S1), we measured v_p and v_s of a rhenium foil (25 μm in thickness, Nilaco) at ambient conditions (IXS-Re-foil, in air) which was pre-compressed to a thickness of about 10–15 μm. Figure S2A shows the typical IXS spectra at ambient conditions. The velocities, v_p and v_s, of rhenium at ambient conditions measured using IXS (Fig. S2B) are consistent with ultrasonic (US) work (*32*) (Fig. 1B). Thus, we confirmed that the experimental v_p and v_s of rhenium with IXS method are reasonable.

S2. Strong intensity of transverse acoustic mode

A crucial point in the present work is that the IXS spectra (Figs. 1A and S2A) showed clear peaks that we could consistently associate with both the longitudinal acoustic (LA) and transverse acoustic (TA) modes of rhenium, thereby allowing us to derive both acoustic velocities, v_p and v_s. This is different than IXS measurements of iron-rich materials under pressure which generally only show the peak from the LA mode (e.g., *8, 52, 53*). As discussed below, experimental and calculational investigations suggest the appearance of the lower energy TA peak is the result of a quite high defect density in hexagonal close-packed (hcp) rhenium after it has been pressurized once. It is possible that these defects might shift the energies of the LA and TA peaks so that the peaks are not simply related to the relevant acoustic velocity. The extremely simplified models discussed below suggest such shifts are possible. However, ambient pressure measurements show that, in the real samples, it is reasonable to associate the position of these peaks with the TA and LA mode energies. In particular, we measured IXS spectra from a once-compressed rhenium foil and found that, after treating that data in the same way as we do the high-pressure data, we obtained the known ambient v_p and v_s (see Note S1). Thus, it is reasonable to associate the two peaks with the TA and LA modes at ambient pressure, and we continue to do so at high pressure. However, as this is important, we also discuss our work in more detail.

From the viewpoint of both single crystal calculations (Fig. S3) and previous IXS measurements of iron-rich materials by powder samples, the TA mode intensity at the momentum transfers used in the present work is expected to be quite weak, so the large intensity observed here requires explanation. While we initially speculated that complex (elliptical) eigen-polarizations for acoustic modes in rhenium might account for the relatively large TA intensity, in fact calculations using a first principles model that was well validated by single crystal measurements showed this was not the case: the calculated intensity of the TA mode, in a Born approximation, was much too small to account for the observed spectra (see the black lines in Fig. S3). As the appearance of the intensity in this region and the association of that intensity with the TA mode is critical to our work, the calculation of negligible TA intensity spawned an investigation to determine the origin of the observed intensity. We should add that similar modelling was used to make estimates of the spectra in the presence of strong texture, or with a large (larger than observed) *c*-axis strain. In all cases the calculated spectra had much less intensity near the "TA" peak position than our measurements. This

suggests then that either (see Note S2-A) we are not in the Born approximation limit, or (see Note S2-B) that the structure appropriate for describing the single crystal measurements was not appropriate for our powder samples. We discuss each of these in turn.

S2-A. The possibility that the low energy, "TA", intensity originated from multiple scattering, or violation of the Born approximation, was ruled out by a mixture of experiment and calculation. We calculated the energy dependence of the largest multiple scattering correction, namely the double scattering process of powder diffraction (elastic Bragg scattering) followed by inelastic phonon scattering, or vice versa. Given, the x-ray diffraction (XRD) powder patterns from the sample indicate the presence of many grains within the beam spot, and the relatively small phonon cross section, we assumed no correlation between the orientation of the grain where the powder diffraction occurred and the grain where the phonon scattering takes place. That calculation, for an isotropic powder, using the well-validated phonon model mentioned in the previous paragraph, leads to a density-of-states-like contribution to the scattering that peaks strongly at about 14–15 meV and is nearly independent of momentum transfer. Both the shape and the momentum dependence do not agree with the observed intensity, suggesting any such multiple scattering contribution is small. Further, measurements at two different indentations on one foil, where the transmitted intensity varied by a factor of two, showed that the ratio of the intensity of the TA and LA peaks did not change so much when the sample thickness was changed, while one would expect that if the "TA" peak were due to multiple scattering, it would scale differently than the LA peak. These results lead us to rule out multiple scattering.

S2-B. The real structure of once-compressed rhenium was determined to be different than that of a single crystal using electron microscopy. The microstructure of a once-compressed rhenium foil was observed by transmission electron microscopy (TEM) with electron diffraction patterns operated at 200 kV (JEM-2100F, JEOL) at Tohoku University. A TEM image is shown in Fig. S4 with defect locations as indicated. There is a high defect density, ~0.1–1 nm^{-2}. The full impact of such defects on phonon spectra is difficult to calculate, both because the exact defect structure is not easily determined and because the system it is necessary to calculate becomes very large. However, we made estimates using some simpler models with a twin boundary included and either 16 or 32 atoms per primitive cell. While these are dramatic simplifications, the calculations show (see Fig. S3) that the intensity in the TA region becomes larger, becoming similar in scale to the experimental IXS spectra. Further, cluster calculations using the force constants from the perfect crystal model were also tried and those also showed similar intensity in the TA region when the size of the clusters was reduced to a few nm scales. Thus, several calculations, albeit of simpler systems, suggest defects on a nm length scale can adequately account for the "TA" intensity. The measurements mentioned above at ambient pressure then suggest it is reasonable to use the observed peak position to derive the relevant acoustic velocities.

S3. Comparison of compression and acoustic velocities with and without pressure medium

Figure S5A shows the experimental c/a ratios of rhenium for direct compression without pressure medium and those for quasi-hydrostatic compression with periclase (MgO) pressure

medium and laser annealing. The c/a ratios with pressure medium are about 1.61, which are consistent with the calculated model c/a ratio of rhenium (*31*). This is one of the proofs that the pressure medium and laser annealing were working well to keep quasi-hydrostatic compression. On the other hand, those without pressure medium show strong uniaxial compression in the c-axis direction. Figure S5B shows the relations between density, ρ, and v_p, and ρ and v_s of rhenium at high pressure and ambient temperature. As shown in Fig. S5B, both v_p and v_s measured with or without pressure medium and laser annealing are consistent within experimental errors, and the v_p and v_s measured at ambient conditions are also consistent with those of US measurement (*32*). The detailed explanations of small effects of uniaxial compression and uncertainty analysis are discussed in Notes S4 to S10.

S4. Uncertainty of fitting for the phonon dispersion and acoustic velocity

Figure S6A shows the Q_{max} value for the fitting of phonon dispersion of rhenium and the distance to the first Brillouin zone (BZ) of rhenium. To obtain v_p or v_s from the IXS results in this work, the dispersion was fitted by v_p or v_s and Q_{max} as free parameters with Eq. 2. However, Q_{max} is also related to the first BZ, as mentioned in the "Phonon dispersion and fitting" section in Methods. To evaluate the fitting and Q_{max} estimation, we compared the v_p and v_s derived by using Q_{max} as a free parameter with those by using Q_{max} fixed to the averaged value over the boundary of the first BZ in Fig. S6B. Both results are quite consistent within the errors for either v_p or v_s obtained by Q_{max}-free or Q_{max}-fixed fittings.

S5. Preferred orientation analysis

In high-pressure experiments, hcp structure metals were known to have strong preferred orientations and lattice strains (*8, 53, 66*). Therefore, to estimate the accurate acoustic velocity, we should consider those effects. Figure S7 shows the experimental and calculated XRD patterns with the experimental preferred orientation or the calculated random orientation at 230 GPa (IXS-Re-12). As shown in Fig. S7A, observed intensities change depending on the azimuth angle in the experimental XRD pattern. Such intensity gradations indicate a strong preferred orientation (*8, 66*). To investigate the preferred orientation condition, we performed the XRD calculation based on the method of whole two-dimensional diffraction pattern fitting (*8, 55*). Considering the sample as an assembly of small crystal grains having individual orientations, the XRD pattern is expressed as the sum of the diffraction from all crystal grains. Therefore, the calculated XRD pattern is estimated as:

$$\mathrm{XRD_{calc}} = \frac{I_0}{N}\left[\sum_{\varphi_1}\sum_{\varphi_2}\sum_{\varphi_3} w_{(\varphi_1,\varphi_2,\varphi_3)}\ \mathrm{XRD}_{(\varphi_1,\varphi_2,\varphi_3)}\right] + \mathrm{BKG}\ , \qquad \text{(S1)}$$

$$N = \sum_{\varphi_1}\sum_{\varphi_2}\sum_{\varphi_3} w_{(\varphi_1,\varphi_2,\varphi_3)}\ , \qquad \text{(S2)}$$

where I_0 is a constant which depends on the experimental conditions such as the sample thickness, exposure time, and sensitivity of the detector. $w_{(\varphi_1,\varphi_2,\varphi_3)}$ is the weight of a crystal grain having orientation given by the Euler angles φ_1, φ_2, and φ_3. $\mathrm{XRD}_{(\varphi_1,\varphi_2,\varphi_3)}$ is the diffraction pattern by a crystal grain having orientation given by φ_1, φ_2, and φ_3. BKG is the background of the XRD pattern. The

XRD pattern is calculated by optimizing the weights, $w_{(\varphi_1,\varphi_2,\varphi_3)}$, to minimize the square of residuals between the experimental and calculated XRD patterns. Figure S7B shows the calculated XRD pattern in the preferred orientation conditions at 230 GPa (IXS-Re-12) with 5-degree increments for each of φ_1, φ_2, and φ_3 (i.e., 72×18×12 independent orientations by the hexagonal symmetry). Compared with the experimental and calculated XRD patterns, the goodness of fitting values, reduced chi-square (χ_v^2) is 2.262 and weighted reliable factor (R_w) is 10.0%. Figure S7C shows the calculated XRD pattern by using lattice parameters at 230 GPa (IXS-Re-12) in a random orientation condition. Compared with the experimental and calculated XRD patterns, the goodness of fitting values, χ_v^2 is 63.27 and R_w is 69.0%. Figure S7, D and E shows the integrated XRD patterns of Fig. S7, A to C. The calculated XRD pattern with the preferred orientation is consistent with the experimental XRD pattern while the calculated XRD pattern with a random orientation is not.

Figure S8 shows the typical preferred orientation conditions at 32 GPa (IXS-Re-01) and 230 GPa (IXS-Re-12). The *c*-axis was generally close to the compression axis as shown in Fig. S8, A and B. However, this tendency was reduced with increasing pressure. The *a*-axis was also preferred in the plane that is perpendicular to the compression axis in lower pressure conditions, but again this tendency was reduced with increasing pressure as shown in Fig. S8, C and D. Figure S9 shows the concentration of *c*-axis in specific directions that is the direction inclined about 10 degrees in the vertical direction to compressional axis in all crystal grains as a function of density. For direct compression experiments, at 32 GPa (ρ = 22.60 g cm^{-3}), *c*-axis in all crystals grains has been ten times concentrated in the specific direction around ±20 degrees regions compared with a random orientation, and even at 230 GPa, *c*-axis in all crystal grains still has been seven times concentrated in the specific direction. In contrast, for pressure medium experiments, there are still preferred orientation, but the concentrations are reduced to one-half or one-third of the value in the direct compression experiments.

S6. Acoustic velocity anisotropy

The hcp structure has five independent elastic moduli C_{11}, C_{12}, C_{13}, C_{33}, and C_{44}:

$$C_{ij} = \begin{pmatrix} C_{11} & C_{12} & C_{13} & 0 & 0 & 0 \\ C_{12} & C_{11} & C_{13} & 0 & 0 & 0 \\ C_{13} & C_{13} & C_{33} & 0 & 0 & 0 \\ 0 & 0 & 0 & C_{44} & 0 & 0 \\ 0 & 0 & 0 & 0 & C_{44} & 0 \\ 0 & 0 & 0 & 0 & 0 & C_{66} \end{pmatrix}, \tag{S3}$$

where

$$C_{66} = \frac{C_{11} - C_{12}}{2} . \tag{S4}$$

The anisotropy of v_p and v_s depends on the direction of the crystal lattice orientation and is derived from the C_{ij} as follows (*67*):

$$v_{p(\psi)} = \sqrt{\frac{C_{11}\sin^2\psi + C_{33}\cos^2\psi + C_{44} + C_{(\psi)}}{2\rho}}\ , \qquad \text{(S5)}$$

$$v_{sv(\psi)} = \sqrt{\frac{C_{11}\sin^2\psi + C_{33}\cos^2\psi + C_{44} - C_{(\psi)}}{2\rho}}\ , \qquad \text{(S6)}$$

$$v_{sh(\psi)} = \sqrt{\frac{C_{66}\sin^2\psi + C_{44}\cos^2\psi}{\rho}}\ , \qquad \text{(S7)}$$

where

$$C_{(\psi)} = \sqrt{\left[\left(C_{11} - C_{44}\right)\sin^2\psi - \left(C_{33} - C_{44}\right)\cos^2\psi\right]^2 + \left(C_{13} - C_{44}\right)\sin^2 2\psi}\ , \qquad \text{(S8)}$$

ρ is density, $v_{p(\psi)}$, $v_{sv(\psi)}$, and $v_{sh(\psi)}$ are compressional, vertically polarized shear, and horizontally polarized shear wave velocities in the direction of ψ, respectively, and the ψ is the angle between the *c*-axis (in this study, approximately the compression axis as shown in Figs. S8 and S9) and the momentum transfer. As shown in Eqs. S3 to S8, the estimation for acoustic velocity anisotropy depends on the elastic moduli, C_{ij}. It is difficult to precisely estimate the C_{ij} under non-hydrostatic conditions, however, as shown in Fig. 2, v_p in this study is consistent with the first principles generalized gradient approximation (GGA) calculation of ref. (*36*), and v_s in this study is consistent with the GGA calculation of ref. (*31*), respectively. Therefore, we used two calculated C_{ij} (*31, 36*) to evaluate the impact of the anisotropy. Figure S10 shows the anisotropy of the v_p and v_s for rhenium and the differences between the experimental v_p and v_s in the preferred orientation conditions and calculated v_p and v_s in a random orientation condition. Regardless of which of the two calculated C_{ij} was used for the evaluation, both anisotropies of v_p and v_s for rhenium can be estimated as within ±10%, depend on the ψ angle (Fig. S10, A and B). However, we should also consider the preferred orientation to estimate the experimental anisotropy of acoustic velocities, because as shown in Fig. S10, A and B, both acoustic velocities in hcp structure depending on the direction ψ, calculated from two calculated C_{ij} (*31, 36*), are similar to the velocities in a random orientation at ψ ~20–30 and ~70–80 degrees, and our experimental probability densities of *c*-axis were concentrated around ψ = 80 degrees (Figs. S8 and S9). Therefore, the actual impacts of anisotropies were expected to be smaller than the maximum deviations of ±10%. The acoustic velocities in the preferred orientation, v_{PO}, were estimated from the harmonic mean of calculated $v_{(\psi)}$ bases on the C_{ij} (*31, 36*) weighted by the experimental probability densities (Figs. S8, S9, and S10, A and B) of *c*-axis as follows:

$$\frac{1}{v_{PO}} = \frac{1}{\pi}\int_0^{\pi} \frac{x_{(\psi)}}{v_{(\psi)}}\,d\psi\ , \qquad \text{(S9)}$$

where $x_{(\psi)}$ is the normalized weight derived from the experimental preferred orientation as:

$$\frac{1}{\pi}\int_0^{\pi} x_{(\psi)}\,d\psi = 1\ . \qquad \text{(S10)}$$

Figure S10, C and D shows the estimated velocity differences between the experimentally observed acoustic velocities in the preferred orientation, v_{PO}, and the velocities in a random orientation. As

shown in Fig. S10, C and D, the effects of anisotropy on both v_p and v_s with pressure medium and laser annealing are less than ±0.5%. On the other hand, the effects of anisotropy on both v_p and v_s without pressure medium and laser annealing are slightly large, but still less than ±1.3%. Therefore, the effect of the preferred orientation, and also pressure medium and laser annealing are small, regardless of the differences for C_{ij}, and our experimental v_p and v_s in the preferred orientation can be estimated to be almost consistent with the v_p and v_s in a random orientation. As mentioned in the main text and the "Primary pressure scale derivation" to "Electronic contribution to heat capacity" sections in Methods, our primary scale is based a Debye approximation under hydrostatic conditions. Though the part of our experimental v_p and v_s were under non-hydrostatic conditions and affected by the preferred orientation, we conclude that the hcp structure and the elastic properties of rhenium allow us to apply the procedure of the primary scale and the Debye approximation in the data set of v_p and v_s derived in the present IXS measurements.

S7. Lattice strain analysis

Under hydrostatic pressure, bulk modulus, K, and shear modulus, G, of a strain constant average model (Voigt model) (*68*) and a stress constant average model (Reuss model) (*69*), K_V, K_R, G_V, and G_R are:

$$K_V = \frac{1}{9}\left[2\left(C_{11}+C_{12}\right) + 4C_{13} + C_{33}\right], \tag{S11}$$

$$K_R = \frac{C_A}{C_B}, \tag{S12}$$

$$G_V = \frac{1}{30}\left[12\left(C_{44}+C_{66}\right) + C_B\right], \tag{S13}$$

$$G_R = \frac{5}{2}\left[\frac{C_A C_{44} C_{66}}{C_A\left(C_{44}+C_{66}\right) + 3K_V C_{44} C_{66}}\right], \tag{S14}$$

where

$$C_A = C_{33}\left(C_{11}+C_{12}\right) - 2C_{13}^2, \tag{S15}$$

$$C_B = C_{11} + C_{12} - 4C_{13} + 2C_{33}. \tag{S16}$$

Because the Voigt and Reuss models are strain constant and stress constant average models, the actual bulk modulus and shear modulus have been assumed to lie between the two models as follows:

$$K_{VRH} = \left(1-\alpha\right)K_V + \alpha K_R, \tag{S17}$$

$$G_{VRH} = \left(1-\alpha\right)G_V + \alpha G_R, \tag{S18}$$

where $\alpha = 0.5$, i.e., the Voigt–Reuss–Hill (VRH) average (*70*) ($\alpha = 0$ and $\alpha = 1$ correspond to the Voigt and Reuss models, respectively).

However, non-hydrostaticity should be considered in high-pressure experiments with a diamond anvil cell (DAC), especially without a pressure medium and laser annealing. Figure S11A

shows the azimuth integrated XRD patterns at 230 GPa (IXS-Re-12) with azimuth angle, η. The position of each XRD peak, having *hkl* Miller index, is affected, depending on the azimuth angle, by lattice strains. The total deviatoric strain, $\varepsilon_{\mathrm{exp}}$, experimentally measured under non-hydrostatic pressure, is describes as follows (*71, 72*):

$$\varepsilon_{\mathrm{exp}} = \frac{d_{\mathrm{exp}} - d_{\mathrm{hsp}}}{d_{\mathrm{hsp}}} , \tag{S19}$$

where d_{exp} and d_{hsp} are experimentally measured *d*-spacing values and *d*-spacing values under hydrostatic pressure, respectively. In lattice strain theory, d_{hsp} can be estimated from the gradient of *d*-spacing values with azimuth angle. Here we account for following the treatment by an analytical method for lattice strains (*71, 72*). The angle between the compression axis and the normal to the diffracting crystallographic plane, ψ, is related to the azimuth angle, η, the angle between the compression axis and incident x-ray, ζ, and the diffracting angle, 2θ, by the following relation (*66, 71, 72*):

$$\cos\psi = \cos\eta \sin\zeta \cos\theta - \cos\zeta \sin\theta \ . \tag{S20}$$

The deviatoric strain $\varepsilon_{\psi(hkl)}$ in the angle ψ is describes as follows (*71, 72*):

$$\varepsilon_{\psi(hkl)} = \frac{d_{\psi(hkl)} - d_{\mathrm{hsp}(hkl)}}{d_{\mathrm{hsp}(hkl)}} = Q_{(hkl)}\left(1 - 3\cos^2\psi\right) , \tag{S21}$$

where $d_{\psi(hkl)}$ and $d_{\mathrm{hsp}(hkl)}$ are experimentally observed *d*-spacing value in the angle ψ and *d*-spacing value under hydrostatic pressure, having *hkl* Miller index, respectively. Here, $d_{\mathrm{hsp}(hkl)}$ and $Q_{(hkl)}$ can be derived from a fit of $d_{\psi(hkl)}$ with Eq. S21, and the *c*- and *a*-axis lengths of hcp structure and the density under hydrostatic pressure can be estimated from azimuth integrated XRD patterns. Figure S11B shows the *c*- and *a*-axis lengths obtained from $d_{\psi(hkl)}$ as a function of $(1-3\cos^2\psi)$. Though the observed direction ranges are limited [$(1-3\cos^2\psi)$ ~0.4–1.0] and the direction of hydrostatic pressure [$(1-3\cos^2\psi) = 0$] could not be observed, due to the experimental optical setup, Fig. S11B clearly shows that the *c*-axis was strongly affected by the uniaxial stress and depends on the direction ψ, meanwhile, the *a*-axis was not affected compared with the *c*-axis. This is also consistent with the observed preferred orientation, which shows the *c*-axis being concentrated in the uniaxial compressional direction (Note S5 and Figs. S7 to S9). Figure S11C shows the density differences between experimentally observed densities and estimated densities under hydrostatic pressure calculated from $d_{\mathrm{hsp}(hkl)}$. A shown in Fig. S11C, the differences of the density between under hydrostatic pressure and present experimental conditions are within ±2%, and also, the density under hydrostatic pressure may be larger than experimentally observed densities in this study. This suggests that our rhenium scale may shift towards the high-density side and further away from the previous scales (see Fig. 3). Therefore, in the terms of density, we conclude that the impact of the lattice strains is small on our rhenium scale and does not affect the discussions for the Earth's inner core in the main text.

The effect of lattice strains on the shear modulus (directly related to v_s by Eq. 9, and also related to v_p by Eq. 8) should also be considered. In anisotropic linear elasticity theory, the averaged uniaxial stress component t_{avg} ($= \sigma_{33} - \sigma_{11}$) in the hcp structure, where σ_{11} and σ_{33} are radial and axial stress components, is given by the average of $t_{(hkl)}$ for all *hkl* reflections. However, because all *hkl*

reflections cannot be observed, t_{avg} is estimated from the arithmetic mean of $t_{(hkl)}$ for all experimentally observed *hkl* reflections.

$$t_{\text{avg}} = \overline{t_{(hkl)}} \,, \tag{S22}$$

where $t_{(hkl)}$ is given by $\alpha = 0.5$ (VRH average), Eqs. S11 to S21, and following equations (*71, 72*):

$$t_{(hkl)} = \frac{6}{5}\left\{ \frac{G_{\text{VRH}(hkl)} Q_{(hkl)} \left(3S_0^2 + 19S_0 + 3\right)}{3\alpha\left(S_0 - 1\right)\left[S_0 - \left(3S_0 + 2\right)H_{(hkl)}\right] + 5S_0} \right\} , \tag{S23}$$

$$G_{\text{VRH}(hkl)} = (1-\alpha)G_{\text{V}} + \alpha G_{\text{R}(hkl)} \,, \tag{S24}$$

$$H_{(hkl)} = \frac{h^2k^2 + k^2l^2 + l^2h^2}{\left(h^2 + k^2 + l^2\right)^2} \,, \tag{S25}$$

$$D_{(hkl)} = \left[d_{\text{hsp}(hkl)} \frac{l}{c} \right]^2 , \tag{S26}$$

$$\begin{aligned} G_{\text{R}(hkl)} = \Big\{ & S_{11}\left[1 - D_{(hkl)}\right]\left[2 - 3D_{(hkl)}\right] - \left[S_{12} - 3S_{44}D_{(hkl)}\right]\left[1 - D_{(hkl)}\right] \\ & - \left[S_{13} - 2S_{13}D_{(hkl)} + S_{33}D_{(hkl)}\right]\left[1 - 3D_{(hkl)}\right]\Big\}^{-1} , \end{aligned} \tag{S27}$$

$$S_{11} = \frac{1}{2}\left(\frac{C_{33}}{C_{\text{A}}} + \frac{1}{C_{11} - C_{12}} \right) , \tag{S28}$$

$$S_{12} = \frac{1}{2}\left(\frac{C_{33}}{C_{\text{A}}} - \frac{1}{C_{11} - C_{12}} \right) , \tag{S29}$$

$$S_{13} = -\frac{C_{13}}{C_{\text{A}}} \,, \tag{S30}$$

$$S_{33} = \frac{C_{11} + C_{12}}{C_{\text{A}}} \,, \tag{S31}$$

$$S_{44} = \frac{1}{C_{44}} \,, \tag{S32}$$

$$S_0 = \frac{2\left(S_{11} - S_{12}\right)}{S_{44}} \,. \tag{S33}$$

Figure S11D shows the ratio of uniaxial stress component to shear modulus estimated from the azimuth XRD patterns (Fig. S11A) with the C_{ij} from the GGA calculation of ref. (*32*). The ratio of uniaxial stress component to shear modulus in the direct compression experiments is about 2.7% at 32 GPa and 1.4% at 230 GPa, and those strains affect 1.8% and 1.0% uncertainties to v_{p} at 32 GPa and 230 GPa, respectively, and 1.4% and 0.8% uncertainties to v_{s} at 32 GPa and 230 GPa, respectively, from Eqs. 8 and 9, and decreasing with increasing pressure. On the other hand, the ratio of uniaxial stress component to shear modulus in the pressure medium and laser annealing experiments is about 0.5–1.1%, those strains affect only less than 1% uncertainties to v_{p} and v_{s}.

Although there are some differences in stress components in the experiments with and without pressure medium and laser annealing, the effects of lattice strains on density and acoustic velocity were expected to be canceled out each other. Therefore, we conclude that the effect of the uniaxial stress is small on our rhenium scale in this study.

S8. Density gradient analysis

Due to non-hydrostatic high pressure conditions, the density gradient of the sample across the sample chamber should be considered. Figure S12 shows the density gradient at 230 GPa (IXS-Re-12) from the XRD patterns obtained by 2 μm steps in two direction scans perpendicular to the compression axis, i.e., vertical and horizontal scans of the DAC. Within the 5 μm region around the sample center position, which was irradiated by the full width half maximum (FWHM) size of x-ray beam, density gradients were only 0.05% in both vertical and horizontal direction of the sample chamber. Even in the 15 μm region around the sample center position which was irradiated by the x-ray beam with whole tails (*28*), density gradients were only 0.5%. Thus, density gradients in this study are negligibly small.

S9. Relaxation of preferred orientation and lattice strain

The preferred orientation in the direct compression experiments was observed to be relaxed with increasing pressure (Figs. S8 and S9) and also the lattice strains decreased with increasing pressure (Fig. S11). One possibility to explain this relaxation of the preferred orientation and decreasing the lattice strains is that a uniaxial compression is relaxed with increasing pressure due to increased "cupping" of the diamonds. Under extreme pressures, the culet of diamonds could not keep the flatness and deformed to "cupping" shape (*73*). Figure S13A shows the transmitted x-ray intensity profiles at 32 GPa (IXS-Re-01) and 230 GPa (IXS-Re-12), respectively. The x-ray transmission increased by a factor of four to five from 32 GPa to 230 GPa. This indicates that the sample was compressed to a thickness of 10–20% with increasing pressure. The transmitted x-ray intensity was almost constant over the full sample area at 32 GPa. On the other hand, at 230 GPa, the transmitted x-ray intensity was reduced by ~25% at the center compared to the edge of the sample. Such large differences of x-ray transmission in high pressure conditions indicate large differences in the sample thickness between the sample center position and the sample edge position, i.e., the deformation of the diamond culet or "cupping". Figure S13B shows the estimation of difference in sample thickness between the sample center position and the sample edge position obtained from the transmitted x-ray intensity profiles. The differences were ~0.2 μm, and ~1% to whole sample thickness at 32 GPa (ρ = 22.60 g cm^{-3}). However, the differences increased with increasing pressure and became more than 1 μm after 150 GPa (ρ ~27.5 g cm^{-3}). At 230 GPa (ρ = 30.24 g cm^{-3}), such large difference indicates the sample thickness in sample center position and sample edge position differs ~50% as shown in Fig. S13C. The difference of the sample thickness with compression has two discontinuities correlated with the experimental period of SPring-8 beamtime (2017A, 2017B, and 2018A) as shown in Fig. S13B. In this study, each pressurization was performed within 8 to 24 hours during each experimental period. On the other hand, experimental periods were several months apart. The sample shape might have been deformed from a cylinder shape to a prolate spheroid, and the state of the stress might change from uniaxial

conditions toward semi-hydrostatic conditions with increasing pressure (Fig. S13C). Diamonds deformed slowly and reached equilibrium during a few months, though the pressure increase occurred instantly with compression. This may explain the relaxation of preferred orientation and lattice strain with increasing pressure.

S10. Uncertainty analysis of the pressure scale

The impact of preferred orientation and lattice strain on our rhenium scale has been evaluated in Notes S5 to S9. Figure S14, A to C and Table S3 show contributions of uncertainty of v_p, v_s, and ρ of rhenium by experimental error (Fig. S6), preferred orientation and anisotropy (Figs. S7 to S10), lattice strain (Fig. S11), density gradient (Fig. S12), and thermodynamic properties (e.g., Grüneisen parameter, see the "Primary pressure scale derivation" and "High pressure and high temperature EoSs for hcp-iron and MgO by the MGD model" sections in Methods) to calibrated pressure. If we assumed that each contribution is independent and takes its maximum value, the uncertainties of v_p, v_s, and ρ become ~2–5%, ~5–16%, and ~1%, respectively. Figure S14D shows the uncertainty of calibrated pressure with these maximum deviations. Though it is a difficult task to compare our rhenium scale with previous scales (*24, 25*) because there are large discrepancies even among previous scales (*26*), our compression curve of rhenium is consistent with previous curves within those of respective experimental pressure ranges (P <150 GPa). However, our compression curve differs from the curves using previous scales for rhenium (*24, 25, 29–31*) at higher pressure even considering the maximum uncertainty of our rhenium scale.

S11. Simultaneous compression experiment

In simultaneous compression experiment of rhenium, iron and MgO, we annealed samples at temperatures over 1000 K by a double-sided laser-heating method using a fiber laser (COMPAT system) (*51*) to minimize the deviatoric stress in the samples. Figure S15 shows the c/a ratios of rhenium and hcp-iron under the present simultaneous density measurement of rhenium and iron with the MgO pressure medium and laser annealing. Both experimental c/a ratios of rhenium and hcp-iron are consistent with the calculated model c/a ratios (*31, 36*) even over 200 GPa, which indicate that annealing of the sample worked well to release the deviatoric stress caused by uniaxial compression. Figure S16 show the present calibrated K-primed EoS of MgO. Our present compression curve of MgO is consistent with the curves based on previous scales (*14, 40, 41*) up to those respective experimental pressure range within the uncertainties of our scale (Fig. S16A). On the other hand, our compression curve of MgO cannot be accounted for by a single theoretical approximation. Figure S16B shows the comparison between our curve and several theoretical compression curves of MgO (*74–77*). Our compression curve is consistent with molecular dynamics (*74*) up to ~75 GPa, but it shows better agreement with local density approximation (LDA) with linearized augmented plane wave method (LAPW) (*77*) from ~75 GPa to ~200 GPa. At above 200 GPa, the difference from LDA+LAPW gradually increases, but on the other hand, it is closer to the curve by quantum Monte Carlo method (*76*) within uncertainty. The curve by LDA with pseudopotential (PP) (*75*) is inconsistent with our curve in most experimental range except low pressures (P <20 GPa).

S12. Consistency of our rhenium scale with shock Hugoniot

The reduced isotherms from Hugoniot curve were traditionally used as a primary pressure scale (*29, 30, 65, 78–80*). To consider the validity of our rhenium scale, we evaluated the consistency between our scale and shock Hugoniot. As shown in Figs. 3A and S17A, the calculated shock Hugoniot from the isotherm for hcp-iron calibrated our rhenium scale is consistent with shock Hugoniot of iron (*42*). The shock temperature, T_{Hug}, also can be used to evaluate the validity of our present pressure scale. Figure S17B shows the comparison of calculated $c_{V,\mathrm{m}}$ of hcp-iron on the Hugoniot curve by the three different c_{el} models shown in Eqs. 23 to 25. The $c_{V,\mathrm{m,DM\text{-}zero}}$ model is almost constant as $3R$ (where R is the gas constant). The $c_{V,\mathrm{m,DM\text{-}LTD}}$ model shows about twice of the $c_{V,\mathrm{m,DM\text{-}zero}}$ at higher pressures. In the $c_{V,\mathrm{m,DM\text{-}FEM}}$ model, the contributions of electron is about one-half to one-third of contributions of phonon, that may be consistent with the low-resistivity of hcp-iron at high pressure and high temperature. As shown in Fig. S17A, the calculated Hugoniot curves of hcp-iron by three different c_{el} models show only small differences within the uncertainties. However, the shock temperature, T_{Hug}, strongly depends on the c_{el} model as shown in Fig. S17C (calculation of T_{Hug} is described in the "Calculation of the shock Hugoniot from the isotherm" section in Methods). The experimental T_{Hug} of hcp-iron has large variations (*43–45*), but most of the T_{Hug} can be accounted for by either $c_{V,\mathrm{m,DM\text{-}zero}}$ or $c_{V,\mathrm{m,DM\text{-}FEM}}$ models (Figs. 3B and S17C). Though the differences of T_{Hug} between three $c_{V,\mathrm{m}}$ models increases with pressures, the ρ-deficits at inner core boundary (ICB) conditions by $c_{V,\mathrm{m,DM\text{-}zero}}$ and $c_{V,\mathrm{m,DM\text{-}LTD}}$ models are consistent with 8(±2)% in the $c_{V,\mathrm{m,DM\text{-}FEM}}$ model within the uncertainties (Fig. S17D). Thus, to discuss the Earth's inner core, the differences among $c_{V,\mathrm{m}}$ models, especially between $c_{V,\mathrm{m,DM\text{-}zero}}$ and $c_{V,\mathrm{m,DM\text{-}FEM}}$ models, which can account for experimental T_{Hug}, have only small impact on the present conclusions.

The MGD model is widely used for high pressure and high temperature EoS, and the Grüneisen parameter, γ, together with molar heat capacity at constant volume, $c_{V,\mathrm{m}}$, is critical as it is directly related to thermal pressure. Within the MGD model, the Grüneisen parameter represents the effect of crystal lattice volume change on its vibrational properties (*1, 39*). Therefore, the Grüneisen parameter can be derived from the ρ-dependence of v_{p} and v_{s} as shown in Eqs. 11, 12 and 18. The γ_{th} of hcp-iron was derived from the isothermal bulk modulus, K, by our EoS of hcp-iron with the experimental v_{p} of hcp-iron (*8*) by using $K = K_S$ assumption as the initial value, and iterating for optimization of the parameters of the Debye temperature and the Grüneisen parameter to be consistent with both isothermal and adiabatic bulk moduli, and v_{p} and v_{s}. The details are given in the "High pressure and high temperature EoSs for hcp-iron and MgO by the MGD model" section in Methods. Figure S18B shows the comparison between the initial values of Grüneisen parameter with $K = K_S$ assumption and the final values of Grüneisen parameter after the iterations of five times for optimizations. As shown in Fig. S18B, the difference between the Grüneisen parameters is quite small within the uncertainties and there is almost no effect on conversion from isotherm to the shock Hugoniot. On the other hand, large discrepancies in the Grüneisen parameter exist between the previously proposed value (*5*) and that derived from the experimental v_{p} (*8*) and the EoS of hcp-iron (*5*) as shown in Fig. S18B. This means that our EoS is consistent with both the shock Hugoniot and experimental v_{p} of hcp-iron.

Figure S19A shows our present isotherm, and calculated and experimental Hugoniot of rhenium. Because there is no experimental T_{Hug} data for rhenium, only the differences between the

$c_{V,\mathrm{m,DM\text{-}zero}}$ and $c_{V,\mathrm{m,DM\text{-}LTD}}$ [the electronic specific heat coefficient, Γ_{el}, of rhenium is fixed to 2.29 mJ K^{-2} mol^{-1} (*59*)] models are discussed here. Similar to hcp-iron, the two $c_{V,\mathrm{m}}$ models for rhenium reproduce almost the same Hugoniot curves, which are consistent with the experimental shock Hugoniot (*29, 30*) within the uncertainties, though there are large differences in calculated $c_{V,\mathrm{m}}$ and T_{Hug} on the Hugoniot curve (Fig. S19B). However, a larger difference between the calculated and experimental Hugoniots of rhenium is observed compared with that of hcp-iron, though it is still within the uncertainty. While hcp-iron shows a good agreement, one possible reason for deviation in rhenium is that the present estimate for c_{el} of rhenium, which is a high atomic number element, is not sufficient. The effect of electrons on the $c_{V,\mathrm{m}}$ and the effect of anharmonicity neglected in the approximation of quasi-harmonic motion in rhenium may be larger than hcp-iron. Because rhenium has many electrons and heavier atomic weight than iron, the temperature dependence of the Grüneisen parameter in rhenium may be too large to be ignored. Further experiments are necessary to discuss the high temperature state of rhenium.

Figure S20A shows the calculated shock Hugoniot of MgO (B1, rock salt type cubic structure), which is consistent with the experimental shock Hugoniot (*47, 79, 81*) within uncertainties. The γ_{th} of MgO was derived from the isothermal bulk modulus, K, by our EoS of MgO with the experimental v_{s} of MgO (*57*) by using $K = K_S$ assumption as the initial value, and iterating for optimization of the parameters of the Debye temperature and the Grüneisen parameter to be consistent with both isothermal and adiabatic bulk moduli, and v_{p} and v_{s}. The details are given in the "High pressure and high temperature EoSs for hcp-iron and MgO by the MGD model" section in Methods. Figure S20B shows the calculated T_{Hug} of MgO, which is consistent with the experimental T_{Hug} (*47–50*) within uncertainties.

S13. Summary of our rhenium scale and previous scales

The differences in experimental conditions between our experiments and other experiments for rhenium (*24, 25, 29, 30, 35*) are summarized in Fig. S21. As shown in Fig. S21, previous experiments were calibrated by shock compression data (*29, 30, 78–80*), gas pressure gauge (*82*), and theoretical work (*83, 84*). In most high-pressure experiments, the pressure standard such as rhenium (*24, 25*), gold (*78, 82, 85*), helium (*86*), tungsten (*24, 85*), and ruby (*21, 85, 87*) have been used to calibrate pressure. Those pressure standards are mostly secondary pressure scales and are calibrated by other pressure scales derived by shock compression data (*29, 30, 78–80*) or theoretical work (*83, 84*). Pressures in several previous studies are calibrated by the pressure scales based on the density–velocity relations (*14, 20*) as this study (see also the "Primary pressure scale derivation" section in Methods). However, their applicable pressure ranges, up to 55 GPa (*14*) and 120 GPa (*20*), are below the core–mantle boundary pressures.

Shock compression data has been widely used as a primary pressure standard, but as mentioned in the "Calculation of the shock Hugoniot from the isotherm" section in Methods and Note S12, the shock compression data should be converted to isothermal conditions by Rankine–Hugoniot equations and density dependence of Grüneisen parameters. However, previous isotherms were derived from the estimated Grüneisen parameter, which is not based on acoustic velocity (see the "High pressure and high temperature EoSs for hcp-iron and MgO by the MGD model" and "Electronic contribution to heat capacity" sections in Methods, and Fig. S18B). The density, ρ,

dependence of the Grüneisen parameter, γ, was assumed simply as $\gamma\rho$ = constant due to the lack of the acoustic velocity data at high pressure in previous studies (*29, 30, 78–80*) and the consistency with acoustic velocity was not considered to derive the pressure scale. This might explain the differences between our scale and previous scales.

Supplementary Figures

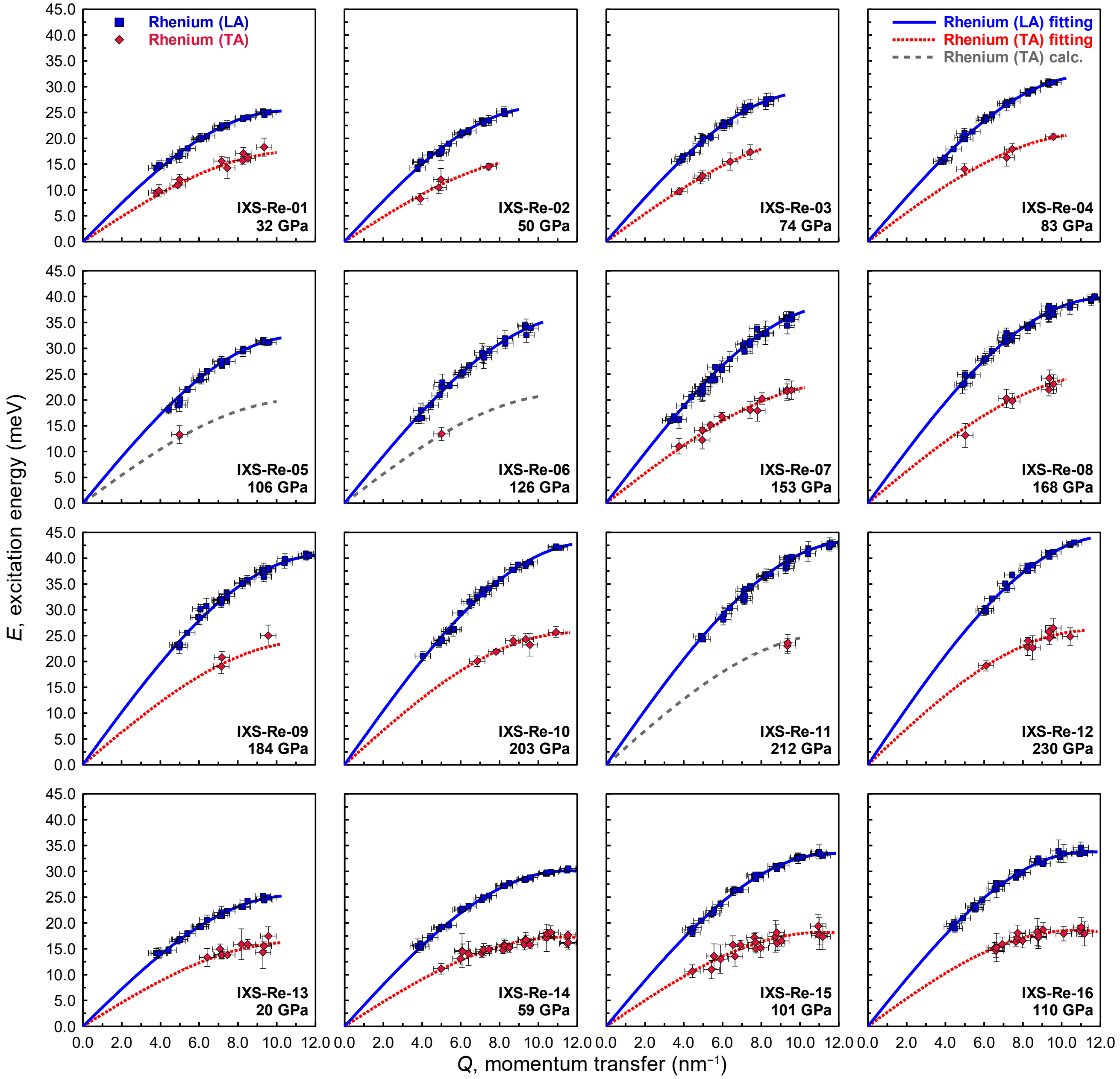

Fig. S1. The phonon dispersion and fits for rhenium in all high-pressure runs. Blue squares are LA modes and red diamonds are TA modes. The horizontal bars give the Q resolution while the vertical bars indicate the one standard deviation (1σ) uncertainties from the fits. Colored lines are the fitting result of phonon dispersion by the sine function; blue solid: LA phonons, red dotted: TA phonons. Gray dashed lines show that partial data sets for TA dispersion are consistent with the interpolation in this work (see main text, Fig. 1B, the "Phonon dispersion and fitting" section in Methods, and Note S4).

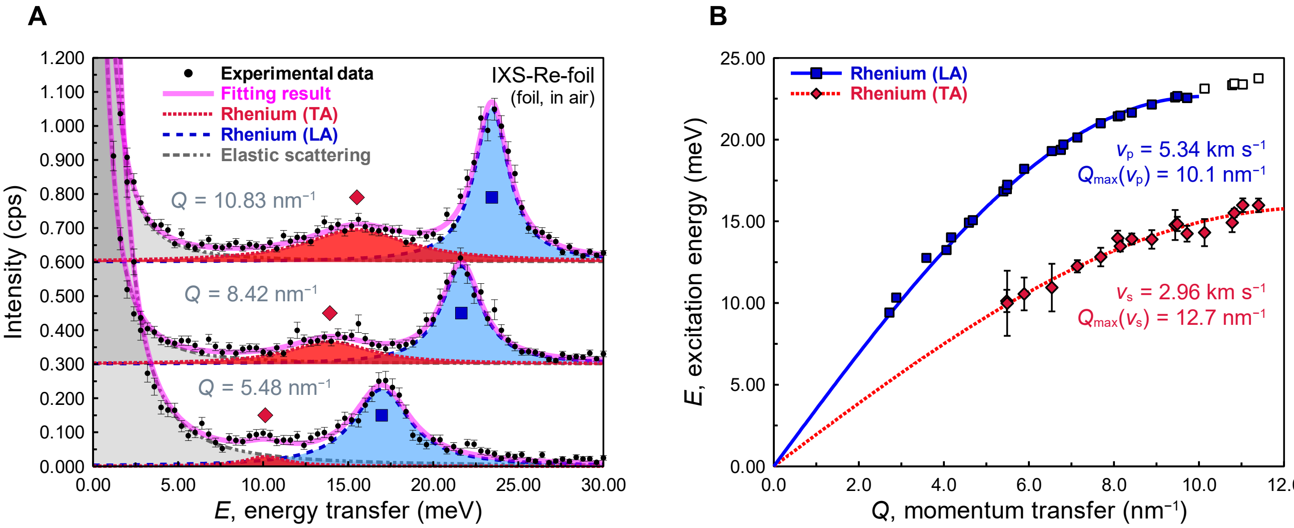

Fig. S2. (**A**) IXS spectra and fitting results for a rhenium foil in ambient conditions after indentation at three different momentum transfers (Q = 5.48, 8.42, and 10.83 nm^{-1}). The black dots are the IXS data with 1σ error bars. Other colored lines and areas are individual inelastic contributions of LA and TA modes as labeled. (**B**) The phonon dispersion and fits for rhenium at ambient conditions. The colored symbols are individual LA and TA modes of rhenium at ambient conditions. The error bars represent the 1σ uncertainties. The colored lines are the fitting result of phonon dispersion by the sine function (Eq. 2); blue: LA phonons, red: TA phonons.

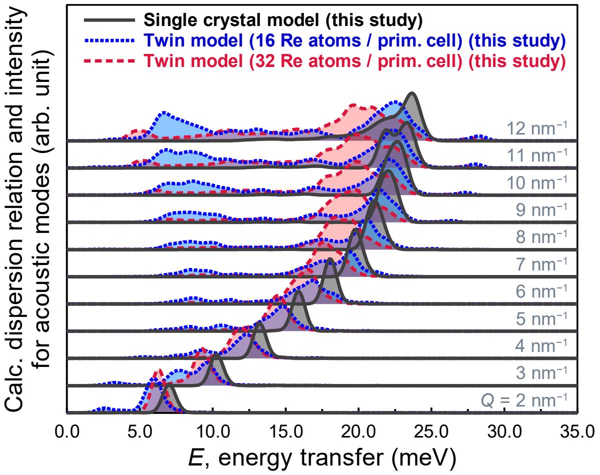

Fig. S3. Calculated IXS spectra for rhenium using three different models including a perfect single crystal model (black solid lines/areas) and two models with a twin boundary with 16 atoms per primitive cell (blue dotted lines/areas) and 32 atoms per primitive cell (red dashed lines/areas). The momentum transfer Q of each line is indicated on the right side. In each case, the powder spectrum is calculated by keeping the scattering angle fixed and integrating over all possible crystal orientations.

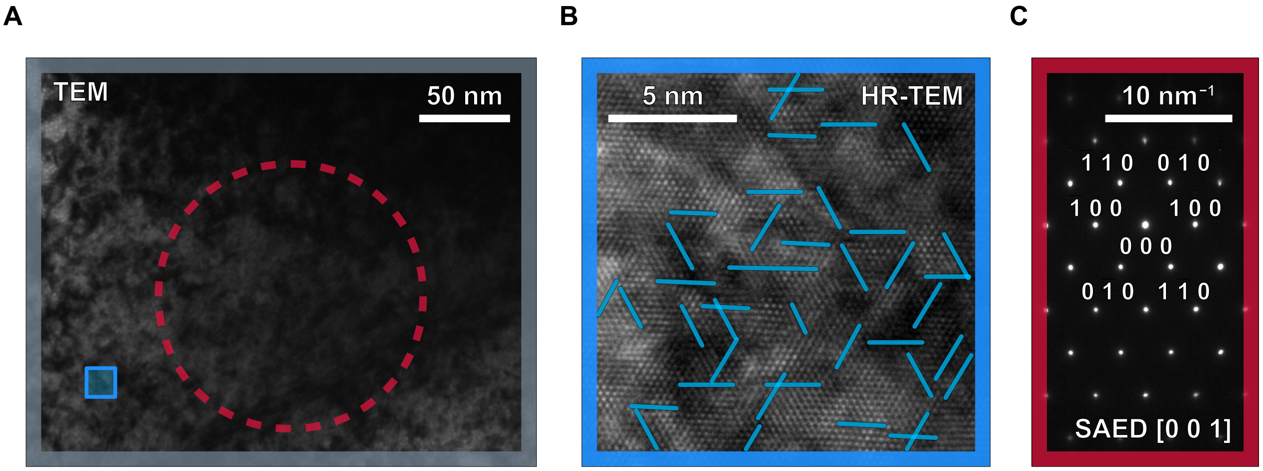

Fig. S4. (**A**) A transmission electron microscopic (TEM) image of ~15 μm indented rhenium foil. The blue square represents the areas of high-resolution (HR) TEM image in (B). The red dashed circle represents the area of the selected area electron diffraction (SAED) pattern measurement in (C). The white line is the scale bar of 50 nm. (**B**) HR-TEM images of the indented rhenium foil. The blue lines in the HR-TEM image show the dislocations observed in the crystallographic direction. The white line is the scale bar of 5 nm. (**C**) The SAED pattern along [0 0 1] zone axis of hcp rhenium. The white line is the scale bar of 10 nm^{-1}.

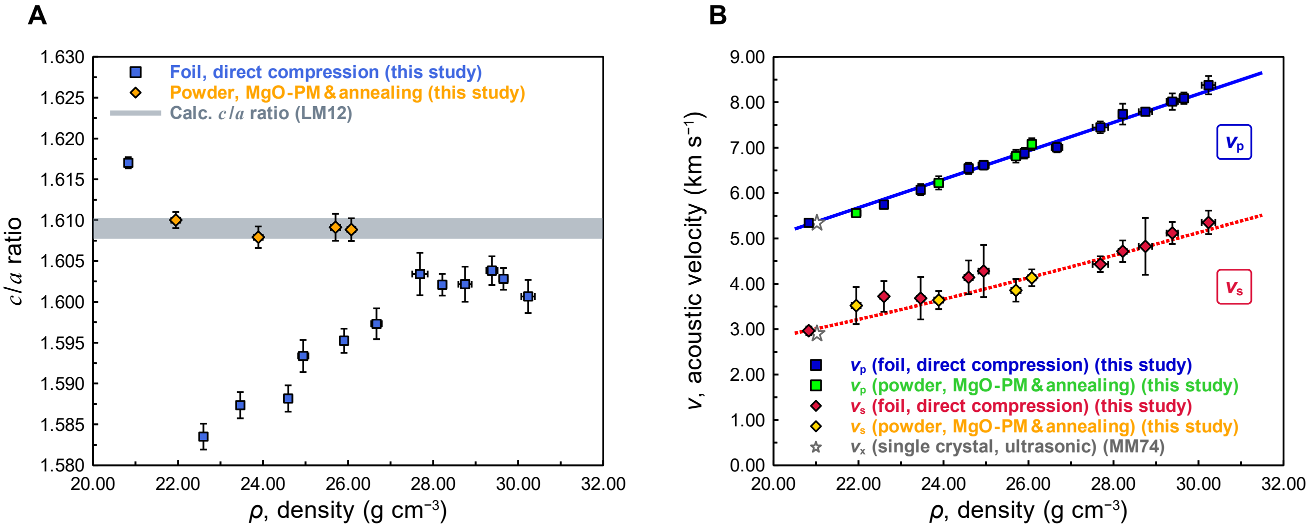

Fig. S5. (**A**) The *c*/*a* ratio of rhenium as a function of density. The color symbols represent the experimental *c*/*a* ratio, blue squares: direct compression experiments (IXS-Re-01 to IXS-Re-12, and IXS-Re-foil) and orange diamonds: experiments with MgO pressure medium and laser annealing (IXS-Re-13 to IXS-Re-16) with 1σ error bars. The gray broad line represents the calculated model *c*/*a* ratio of rhenium (*31*). (**B**) Compressional (v_p) and shear (v_s) wave velocities for rhenium as a function of density. The blue squares and red diamonds are v_p and v_s for rhenium, respectively, by direct compression experiments (IXS-Re-01 to IXS-Re-12, and IXS-Re-foil) with 1σ error bars. The green squares and yellow diamonds are v_p and v_s for rhenium, respectively, by experiments with MgO pressure medium and laser annealing (IXS-Re-13 to IXS-Re-16) with 1σ error bars. The results of two-type experiments are consistent within the errors. Gray star symbols are from the ultrasonic data at ambient conditions (MM74) (*32*).

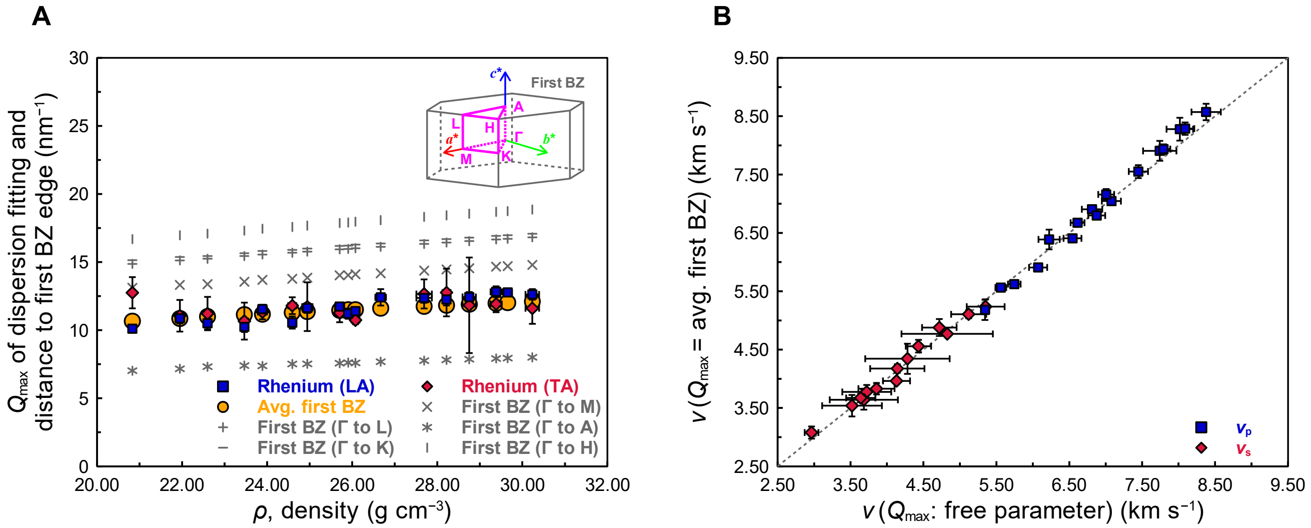

Fig. S6. (**A**) The Q_{max} value used in the fitting. The Q_{max} value from the fitting as shown in Figs. S1 and S2A, and the first BZ calculated from the lattice parameters. The blue square and red diamond symbols represent the Q_{max} value from the fitting for longitudinal acoustic (LA) and transverse acoustic (TA) phonons of rhenium, respectively. The yellow circles represent the averaged first BZ. Each gray symbol represents the first BZ at the critical points (A, M, K, L, and H) of the first BZ that has high symmetry in the hcp structure. The inset represents the first BZ of the hcp structure. Colored arrows represent the reciprocal axis direction (a^*, b^*, and c^*) of the hcp structure and magenta line represents the asymmetric unit for the first BZ of the hcp structure. Typical high symmetry points of the first BZ are Γ(0, 0, 0), A(0, 0, 1/2), M(1/2, 0, 0), K(1/3, 1/3, 0), L(1/2, 0, 1/2), and H(1/3, 1/3, 1/2) in the reciprocal lattice (*88*). (**B**) Comparison of v_p and v_s determined by fitting with Q_{max} as a free parameter or Q_{max} fixed to the averaged value over the boundary of the first BZ. The error bars represent 1σ uncertainties.

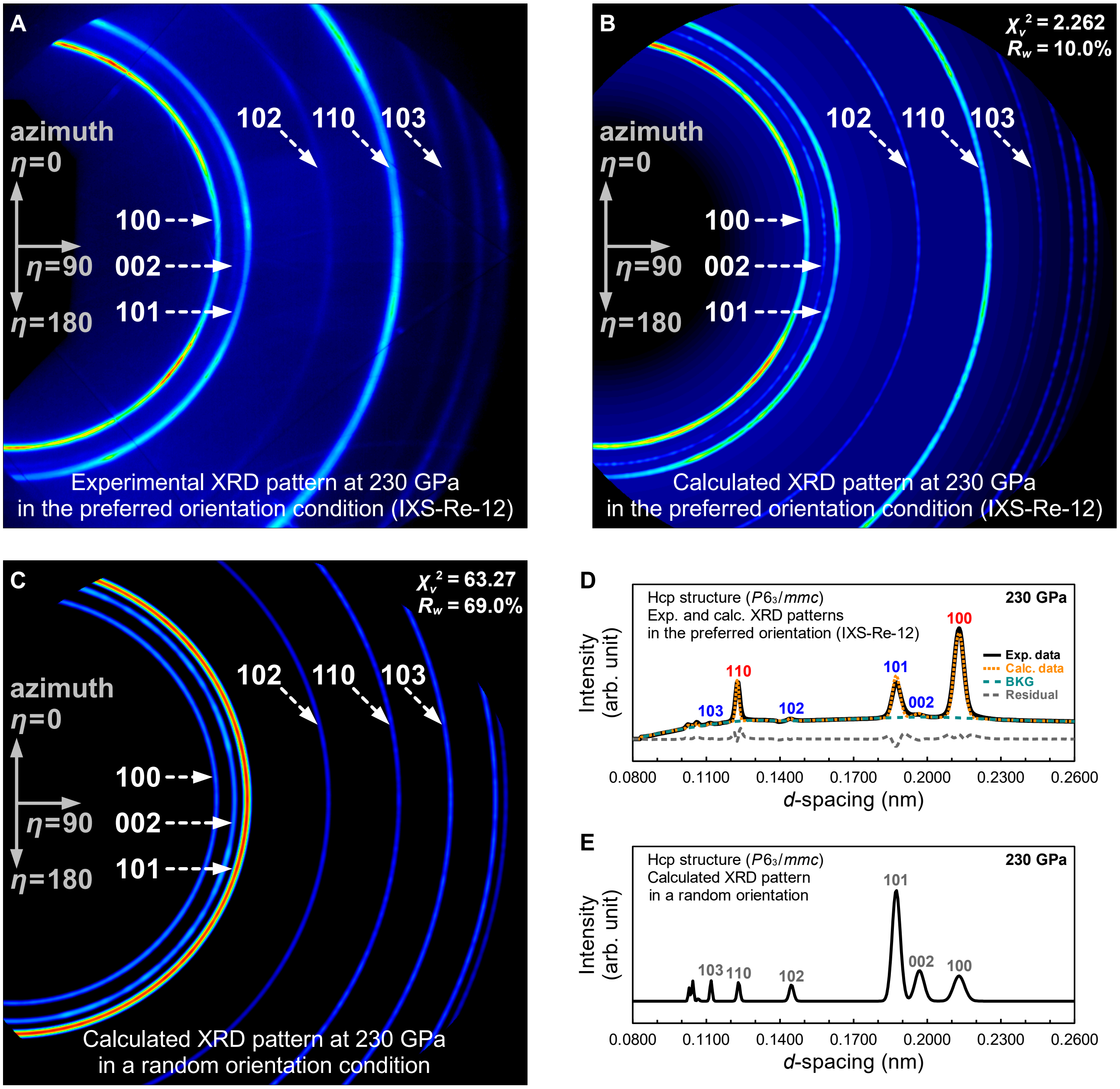

Fig. S7. XRD patterns of rhenium at 230 GPa (IXS-Re-12). (**A**) The experimental XRD pattern in the preferred orientation condition at 230 GPa (IXS-Re-12). The gray arrows represent the azimuth angle of the diffraction. The white numbers give the *hkl* Miller indices of ring. (**B**) The calculated XRD pattern in the preferred orientation condition at 230 GPa (IXS-Re-12). The goodness of fitting parameters compared with the experimental XRD pattern are shown in figures. (**C**) The calculated XRD pattern by using the lattice parameters obtained from experimental XRD pattern and assumed as a random orientation condition at 230 GPa (IXS-Re-12). (**D**) The integrated experimental and calculated XRD profiles in the preferred orientation condition. Black line represents the integrated experimental XRD profile. Each colored dashed and/or dotted line represents the calculated XRD profiles as mentioned in the figure. The colored numbers represent the *hkl* Miller indices of each diffraction. The red and blue numbers in the experimental XRD pattern indicate that the relative intensities are increasing and decreasing compared with the calculated XRD pattern of a random orientation, respectively. (**E**) The integrated calculated XRD profile in a random orientation condition. The gray numbers represent the *hkl* Miller indices of each diffraction.

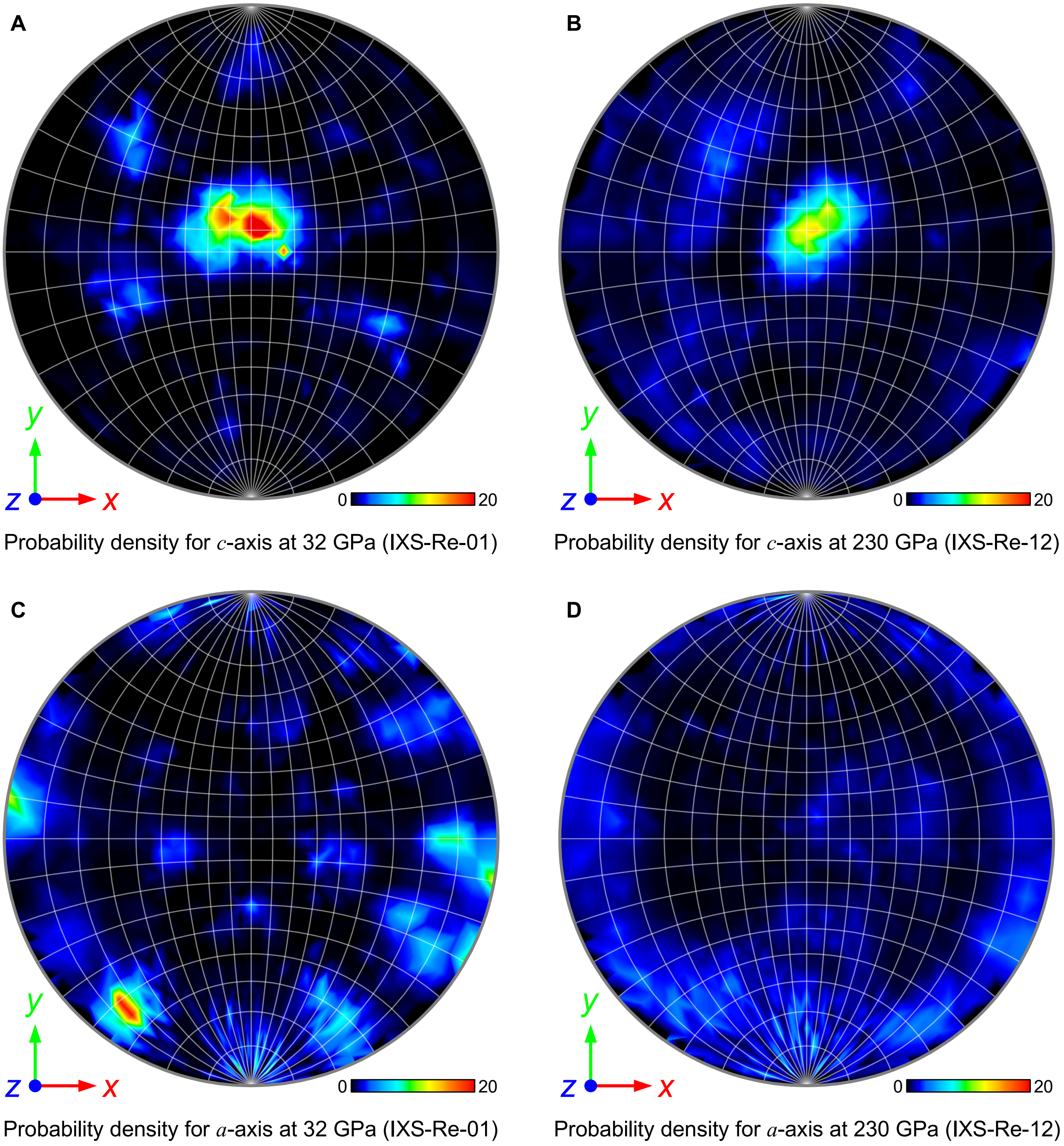

Fig. S8. Experimentally determined stereographic projection of the probability density for *c*-axis and *a*-axis of rhenium showing the preferred orientation. The probability density for *c*-axis at (**A**) 32 GPa (IXS-Re-01) and (**B**) 230 GPa (IXS-Re-12), and *a*-axis at (**C**) 32 GPa and (**D**) 230 GPa. The *x*-*y*-*z* arrows represent each direction of the experimental apparatus; *x*: horizontal, *y*: vertical, and *z*: compressional directions of a DAC, respectively. The color contour bar represents the probability density value for each axis. Under a random orientation condition, the probability density in all directions is 1. The probability density less/greater than 1 means lower/higher probability for crystal grains having the specific directions than at random condition, and 0 means that there is no crystal grain of the direction.

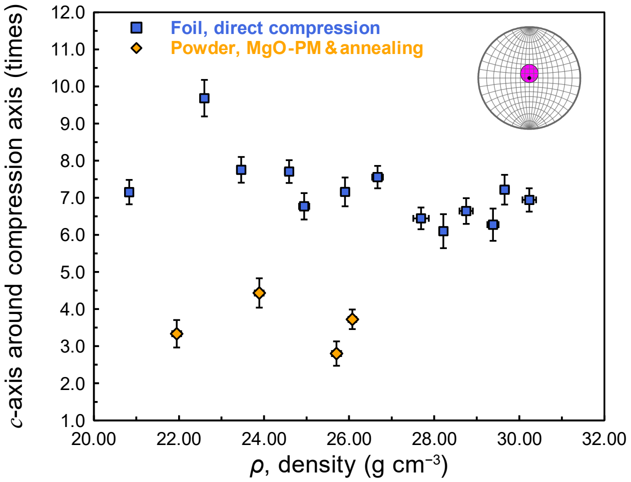

Fig. S9. The concentration of *c*-axis in specific directions for all crystal grains as a function of density. The *c*-axis is concentrated in the direction inclined about 10 degrees in the vertical direction (see Fig. S8). The color symbols represent the concentration of *c*-axis around the specific directions that are shown in the inset which represents the ±20 degrees integration area of the concentration of *c*-axis. The error bars represent the 1σ uncertainties.

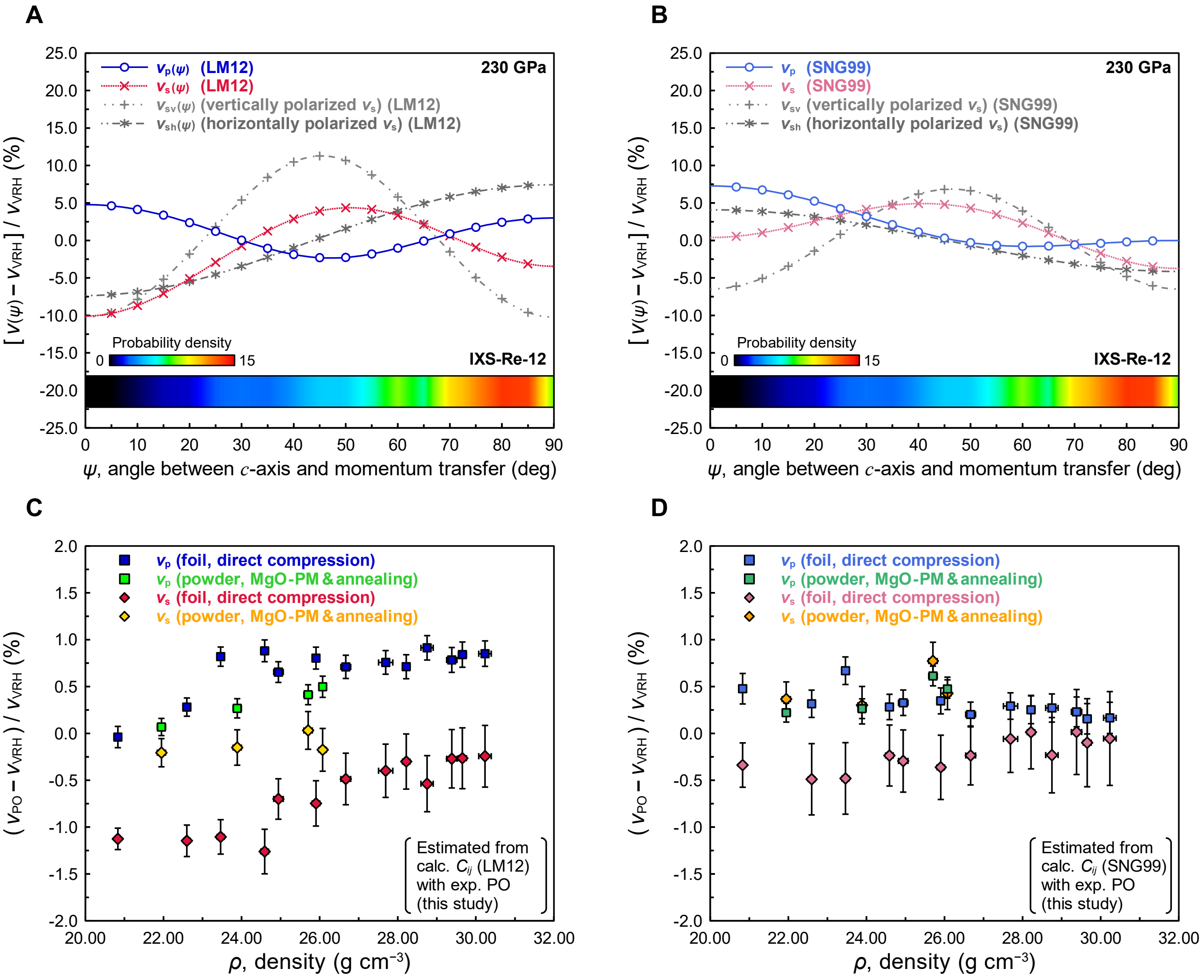

Fig. S10. Estimation of the anisotropy of acoustic velocity in the experimental preferred orientation (PO) conditions. Calculated anisotropies of v_p and v_s based on the C_{ij} by using GGA calculations (LM12) of ref. (*31*) (**A**) and GGA calculations (SNG99) of ref. (*36*) (**B**), respectively, compared with the average v_p and v_s of Voigt–Reuss–Hill average (VRH) as a function of angle, ψ, that is the angle between the *c*-axis (approximately to the compression axis as shown in Figs. S8 and S9) and the lattice vibration direction due to inelastic x-ray scattering. The color bars indicate our experimentally determined distribution of PO. The acoustic velocity difference, $(v_{PO}-v_{VRH})/v_{VRH}$ for v_p and v_s along *c*-axis as a function of density. The $v_{(\psi)}$ and v_{VRH} was calculated from the C_{ij} by using GGA calculations (LM12) of ref. (*31*) (**C**) and GGA calculations (SNG99) of ref. (*36*) (**D**), respectively, with VRH for random orientation conditions. The v_{PO} was calculated with the integral of $v_{(\psi)}$ weighted by the probability density in the direction of ψ (see Eqs. S9 and S10) based on the PO derived from the present XRD patterns (Figs. S7 to S9). Each colored symbol and line are mentioned in the figure. The error bars represent the 1σ uncertainties.

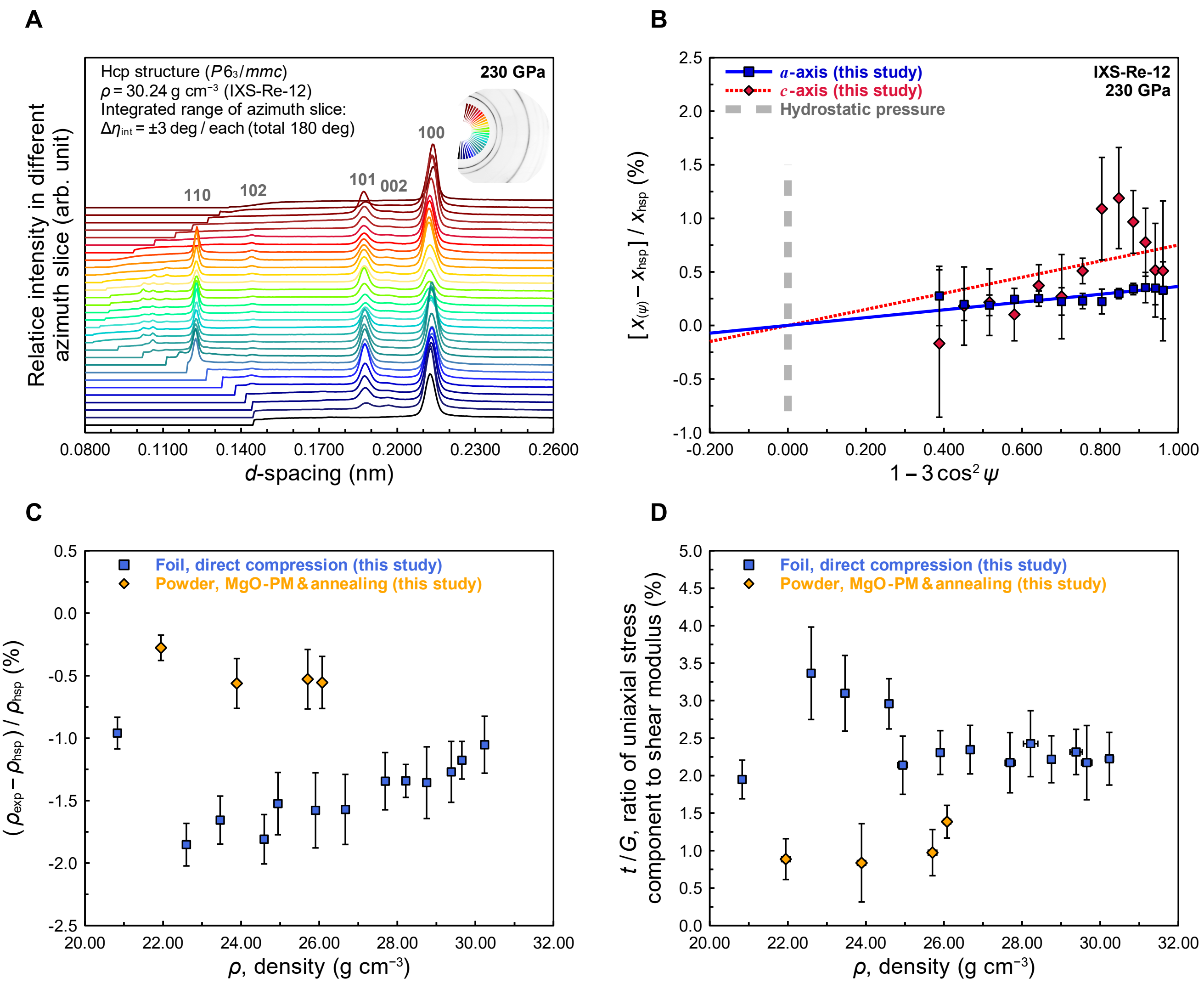

Fig. S11. (**A**) Series of thirty integrated XRD diffraction profiles with 6-degree intervals for different integrated azimuth angle range ($\Delta\eta_{\rm int}$ = ±3 degrees) from η = +8 to +189 degrees at 230 GPa (IXS-Re-12). The analyzed XRD pattern is given in Fig. S7A. The colors in the upper right inset represents the integrated directions in different azimuth slice, corresponding the colored integrated XRD diffraction profiles. The numbers in the figure represent the *hkl* Miller indices. The abrupt intensity changes at small *d*-spacing are due to the opening angle of the DAC and the geometry of the flat panel detector (see Fig. S7A). (**B**) *a*- and *c*- axis length differences, $[x_{(\psi)}-x_{\rm hsp}]/x_{\rm hsp}$, between the experimentally observed *a*- and *c*-axis lengths, $a_{\psi(hkl)}$, and $c_{\psi(hkl)}$ in the direction ψ, and the *a*- and *c*-axis lengths under hydrostatic pressure, $a_{\rm hsp}$ and $c_{\rm hsp}$, estimated from analyzed XRD pattern of rhenium (Figs. S7A and S11A), as a function of $(1-3\cos^2\psi)$, where ψ is the angle between the compression axis and the normal to the diffracting crystallographic plane, blue squares and red diamonds: the *a*- and *c*-axis of rhenium, respectively. The direction of $(1-3\cos^2\psi) = 0$ means the hydrostatic pressure in anisotropic linear elasticity theory (*71, 72*). (**C**) The density difference, $(\rho_{\rm exp}-\rho_{\rm hsp})/\rho_{\rm hsp}$, between the experimentally observed density, $\rho_{\rm exp}$, and the density under hydrostatic pressure, $\rho_{\rm hsp}$, estimated from analyzed XRD pattern (Figs. S7A and S11A) as a function of density, blue squares: direct compression without pressure medium experiments (IXS-Re-01 to IXS-Re-12, and IXS-Re-foil) and orange diamonds: experiments with MgO pressure medium and laser annealing (IXS-Re-13 to IXS-Re-16). The error bars represent the 1σ uncertainties. As shown in figure, $(\rho_{\rm exp}-\rho_{\rm hsp})/\rho_{\rm hsp}$ in all experiments are negative, which means that the experimentally observed density may be smaller than the density under hydrostatic pressure due to the lattice strains. (**D**) Ratio of uniaxial stress component, *t*, to shear modulus, *G*, estimated from the azimuth slices of XRD pattern (Fig. S10A) as a function of density, blue squares: direct compression without pressure medium experiments (IXS-Re-01 to IXS-Re-12, and IXS-Re-foil) and orange diamonds: experiments with MgO pressure medium and laser annealing (IXS-Re-13 to IXS-Re-16). The error bars represent the 1σ uncertainties.

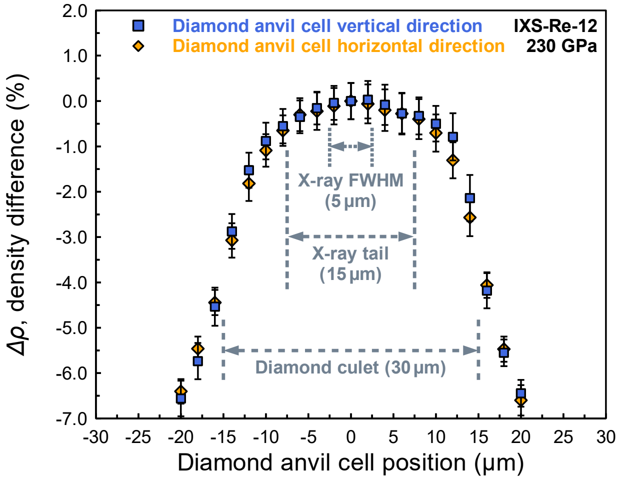

Fig. S12. Density gradient across the sample center at 230 GPa (IXS-Re-12), blue squares and orange diamonds: two-directional (i.e., vertical and horizontal) scans perpendicular to the compression axis of the DAC, respectively. The error bars represent the 1σ uncertainties.

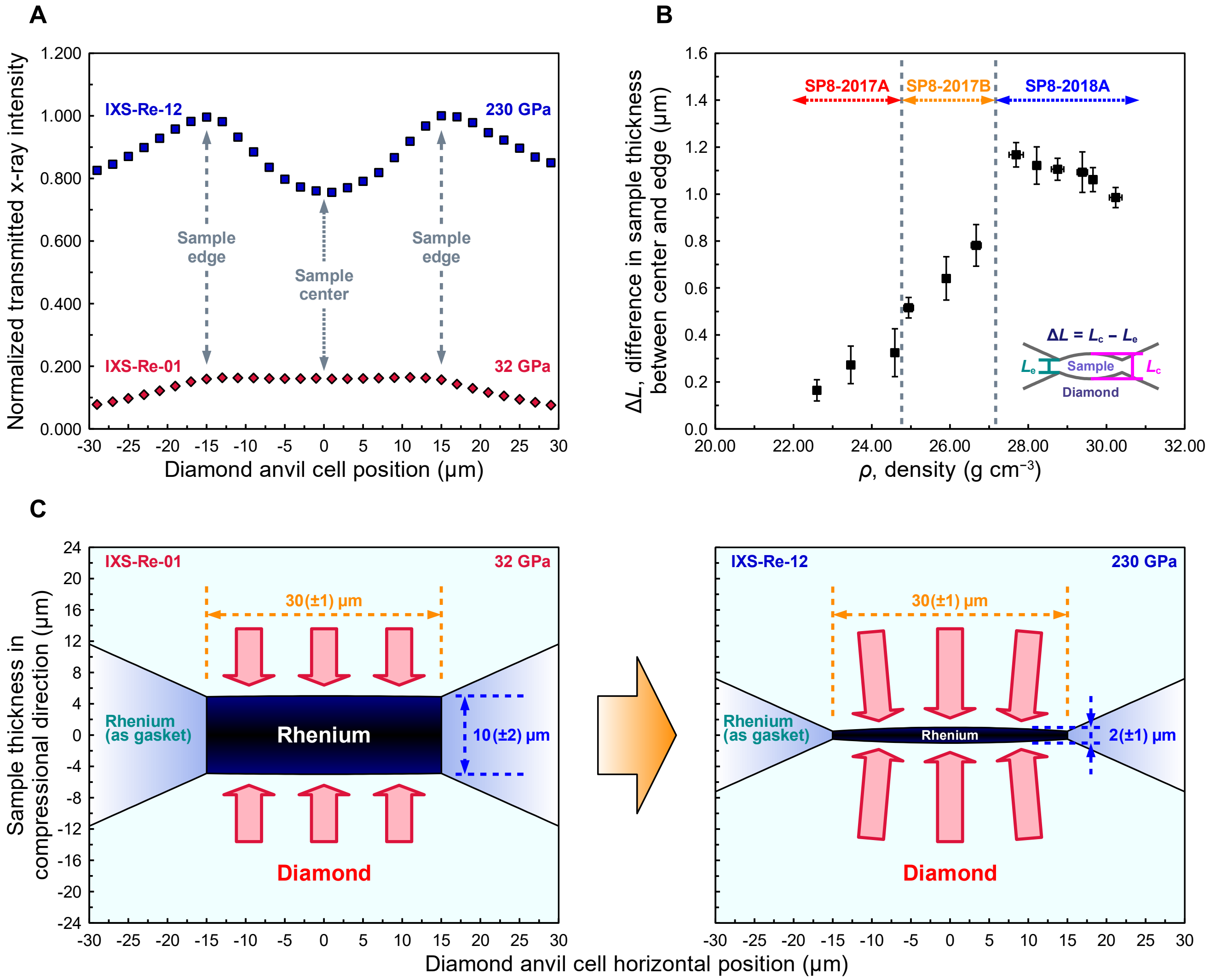

Fig. S13. Deformation of diamond culet. (**A**) X-ray transmission profiles, red diamond: at 32 GPa (IXS-Re-01) and blue square: at 230 GPa (IXS-Re-12). (**B**) Differences in sample thickness between sample center position and the sample edge position as a function of density. The error bars represent the 1σ uncertainties. The colored arrows indicate the SPring-8 beamtime periods as mentioned in the figure. The inset represents the schematic of the difference in sample thickness between sample center position and the sample edge position. (**C**) Schematic images of the diamond deformation at 32 GPa (IXS-Re-01) and 230 GPa (IXS-Re-12), respectively.

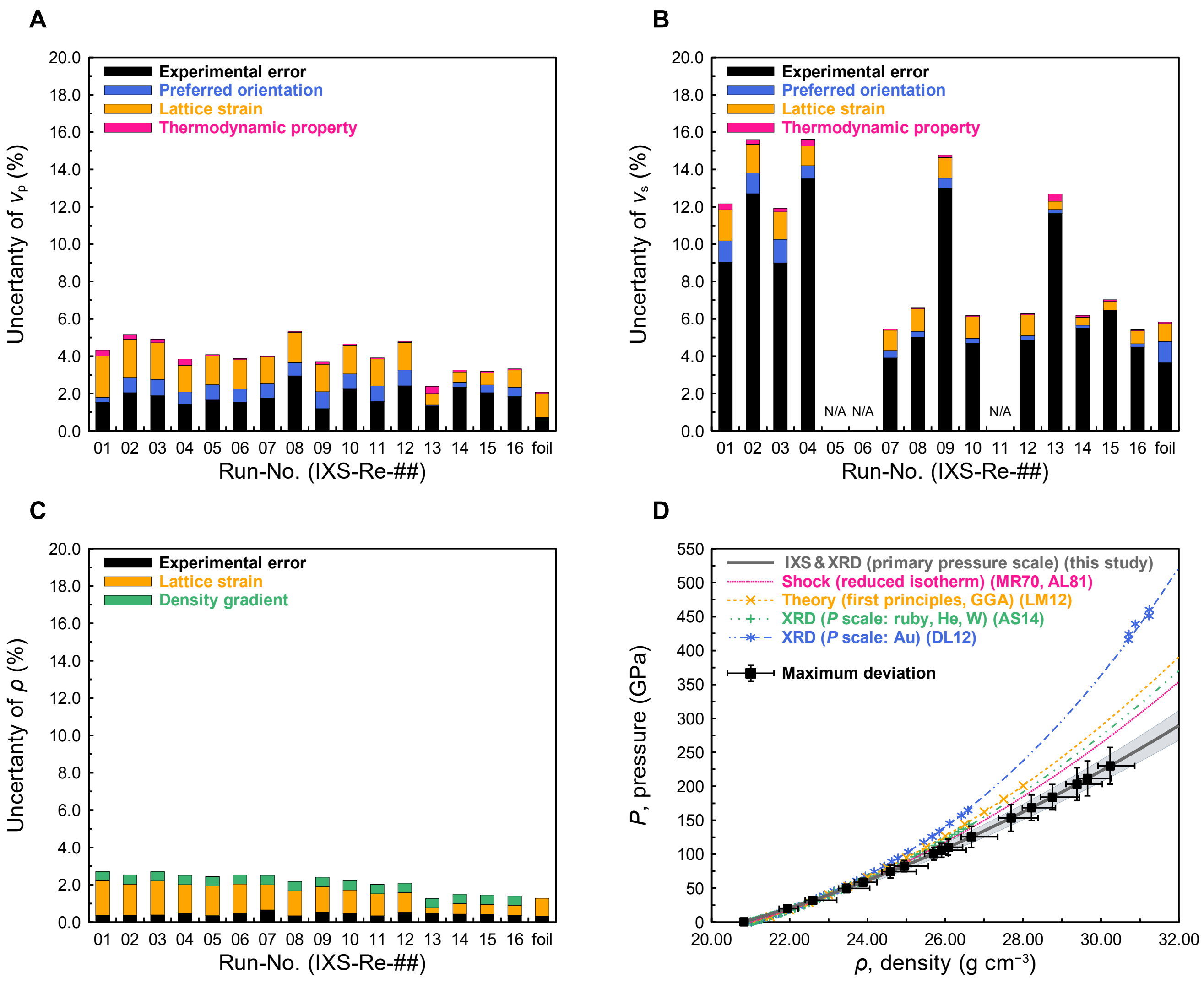

Fig. S14. Error budget contribution of the uncertainties to the rhenium pressure scale. (**A**) Upper bound on the uncertainty of v_p and (**B**) that of v_s from indicated sources. (**C**) Upper bound on the uncertainty of ρ from indicated sources. (**D**) The resulting uncertainty of calibrated pressure (same as Fig. 3A in the main text).

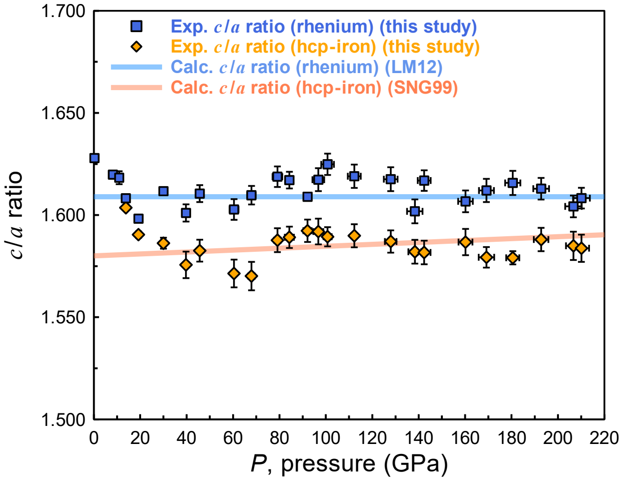

Fig. S15. *c*/*a* ratios of rhenium and hcp-iron under the present simultaneous density measurement as a function of pressure. The color symbols represent the experimental *c*/*a* ratio, blue squares: rhenium and orange diamonds: hcp-iron with 1σ error bars. The blue and orange lines represent the calculated model *c*/*a* ratios of rhenium (*31*) and iron (*36*), respectively.

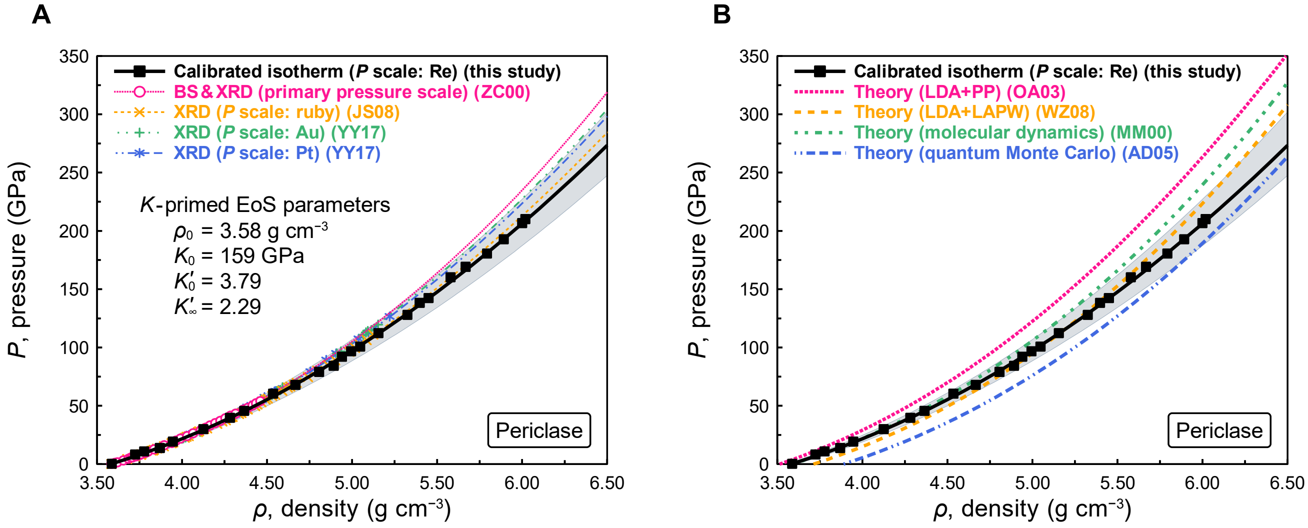

Fig. S16. Re-evaluated density–pressure relation for MgO. The black curve with black squares is the compression curve of MgO re-evaluated by the present simultaneous compression experiment based on our rhenium scale (Tables S2 and S4). The shaded areas around the black curve represent the 1σ uncertainty of our compression curve. (**A**) Comparison with previous experimental compression curves of MgO. Colored curves and symbols are the compression curves of MgO based on pressure scales with experimental data from previous studies [ZC00 (*14*), JS08 (*40*), YY17 (*41*)]. (**B**) Comparison with previous theoretical compression curves of MgO. Colored curves are the compression curves of MgO based on theoretical studies (LDA: local density approximation, PP: pseudopotential, LAPW: linearized augmented plane wave method) [OA03 (*75*), WZ08 (*77*), MM00 (*74*), AD05 (*76*)].

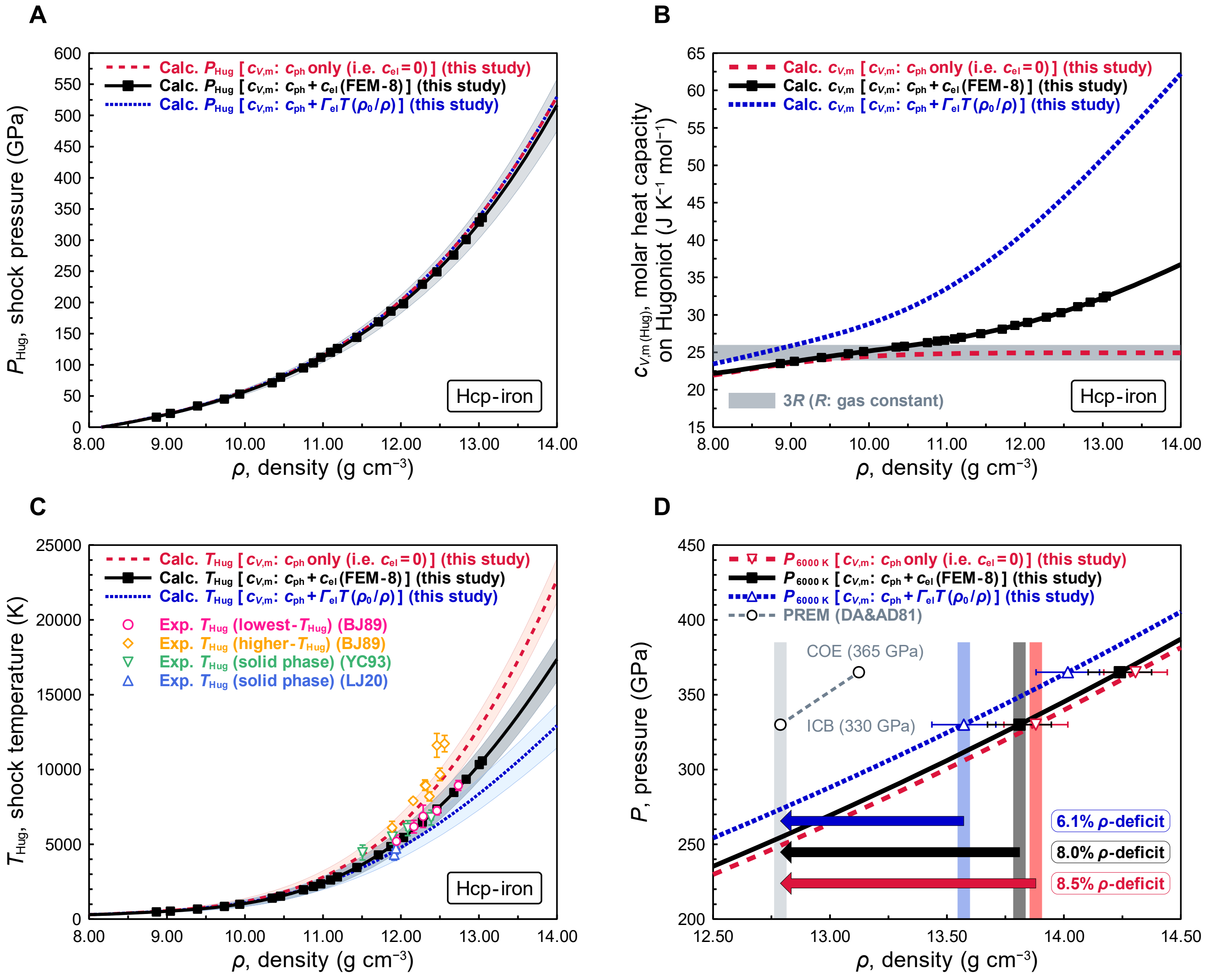

Fig. S17. Summary of thermodynamic properties of hcp-iron on shock Hugoniots and the density deficit of the inner core at 330 GPa and 6000 K with three different c_{el} models. Details of the c_{el} models are given in the "Electronic contribution to heat capacity" section in Methods. The parameters used for calculation are given in Table S2. (**A**) Comparison of calculated shock Hugoniots of hcp-iron based on three different $c_{V,m}$ models. The shaded areas around the black curve represent the 1σ uncertainty of calculated shock pressure, P_{Hug} based on $c_{V,m,DM\text{-}FEM8}$ model. There are no large differences on P_{Hug} between the three models. (**B**) Comparison of calculated $c_{V,m}$ of iron on the Hugoniot curves based on $c_{V,m,DM\text{-}zero}$ (red dashed), $c_{V,m,DM\text{-}LTD}$ (LTD: linear temperature dependence model) (blue dotted), and $c_{V,m,DM\text{-}FEM8}$ (FEM-8: free electron model with eight valence electrons) (black solid) models corresponding to Eqs. 23 to 25 in the "Electronic contribution to heat capacity" section in Methods, respectively. The gray bold line represents $3R$ (where R is the gas constant), the converged value of the contribution of phonons to heat capacity derived from the Debye model. (**C**) Comparison of calculated shock temperature, T_{Hug} of hcp-iron based on three different $c_{V,m}$ models, compared with the experimental T_{Hug} from previous studies [BJ89 (*43*), YC93 (*44*), LJ20 (*45*)]. The symbols and lines are the same as those in Fig. 3B. (**D**) Comparison of density–pressure relations of hcp-iron based on three different $c_{V,m}$ models at 6000 K and the preliminary reference Earth model (PREM). The gray dashed curve with open circles represents the density–pressure relation of the PREM (DA&AD81) (*9*). Other colored lines represent the same as those in (A). The red ($c_{V,m,DM\text{-}zero}$ model), blue ($c_{V,m,DM\text{-}LTD}$ model), and black ($c_{V,m,DM\text{-}FEM8}$ model) arrows indicate the density deficits between hcp-iron and PREM inner core for the compression curves of 6000 K. This analysis of c_{el} models gives the density deficit from hcp-iron via our pressure scale is 8(±2)% in the range 330–365 GPa and 6000 K (ICB: inner core boundary, COE: center of the Earth/core) of the typical estimated Earth's inner core conditions as discussed in the main text.

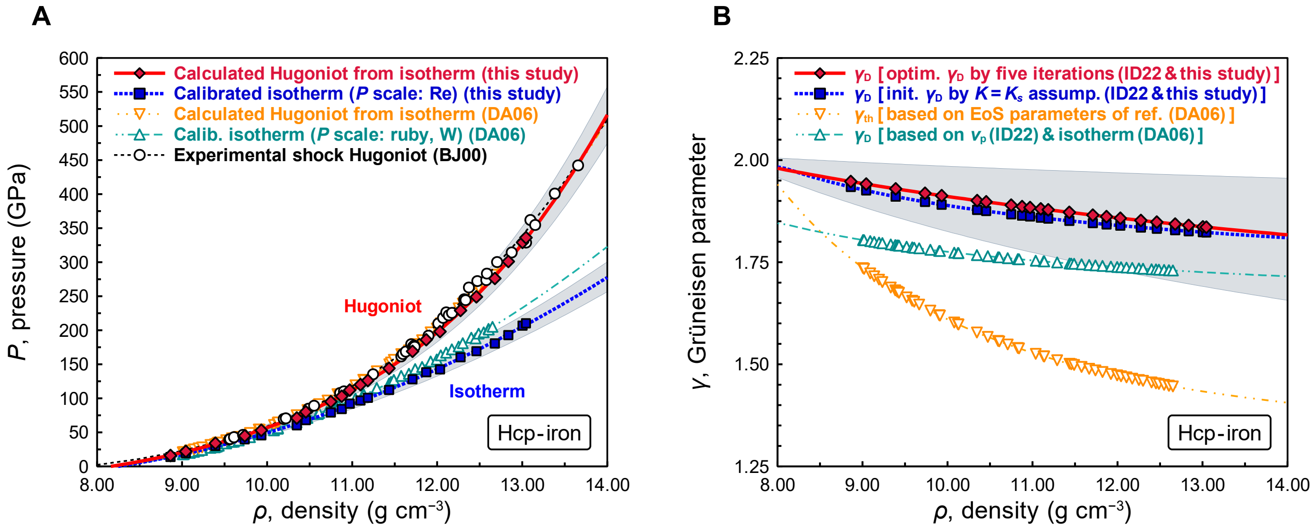

Fig. S18. The isotherm of hcp-iron based on our rhenium scale and calculated shock Hugoniot (**A**) and the Grüneisen parameter used for conversion (**B**). The blue dotted curve with squares in (A) represents the isothermal compression curve of hcp-iron based on our rhenium scale, whereas the green dashed-dotted curve with up-pointing triangles represents the isothermal compression curve based on the previous scale (DA06) (*5*). The black dashed curve with open circles represents the shock Hugoniot with experimental data (BJ00) (*42*). The red solid curve with diamonds represents the calculated shock Hugoniot from the isothermal compression curve of hcp-iron based on our rhenium scale (Tables S5 and S6), whereas the orange dashed-dotted curve with down-pointing triangles represents the calculated shock Hugoniot from the isotherm of the previous scale (DA06) (*5*). Each colored symbol represents density and pressure calculated on each scale, corresponding to the experimental shock compression data (BJ00) (*42*). Our calculated shock Hugoniot based on the Grüneisen parameter derived from the experimental v_p of hcp-iron (ID22) (*8*) can explain the experimental shock Hugoniot (BJ00) (*42*), as well as calculated shock Hugoniot by the previous scale (DA06) (*5*) which is parameterized to account for the shock Hugoniot. Grüneisen parameter of hcp-iron determined from our EoS and experimental v_p of hcp-iron (ID22) (*8*) is shown in (B). We performed iteration for optimization to derive the Grüneisen parameter (details are given in the "High pressure and high temperature EoSs for hcp-iron and MgO by the MGD model" section in Methods). Blue squares show the initial Debye–Grüneisen parameter, γ_D, with $K = K_S$ assumption and red diamonds show the final Debye–Grüneisen parameter (which is equal to the thermodynamic Grüneisen parameter, γ_{th}, in the Debye approximation) after five times iterations for optimization. The difference between both Grüneisen parameters is small within uncertainty and has small effect on the conversion of the isotherm to the shock Hugoniot. On the other hand, a large discrepancy exists between the γ_{th} (yellow down-pointing triangles) proposed by previous EoS (DA06) (*5*) and γ_D (green up-pointing triangles) derived from the EoS (DA06) (*5*) and v_p (ID22) (*8*) of hcp-iron. This means that our EoS of hcp-iron is consistent with both experimental shock Hugoniot and v_p, whereas the previous EoS is inconsistent with v_p determined experimentally.

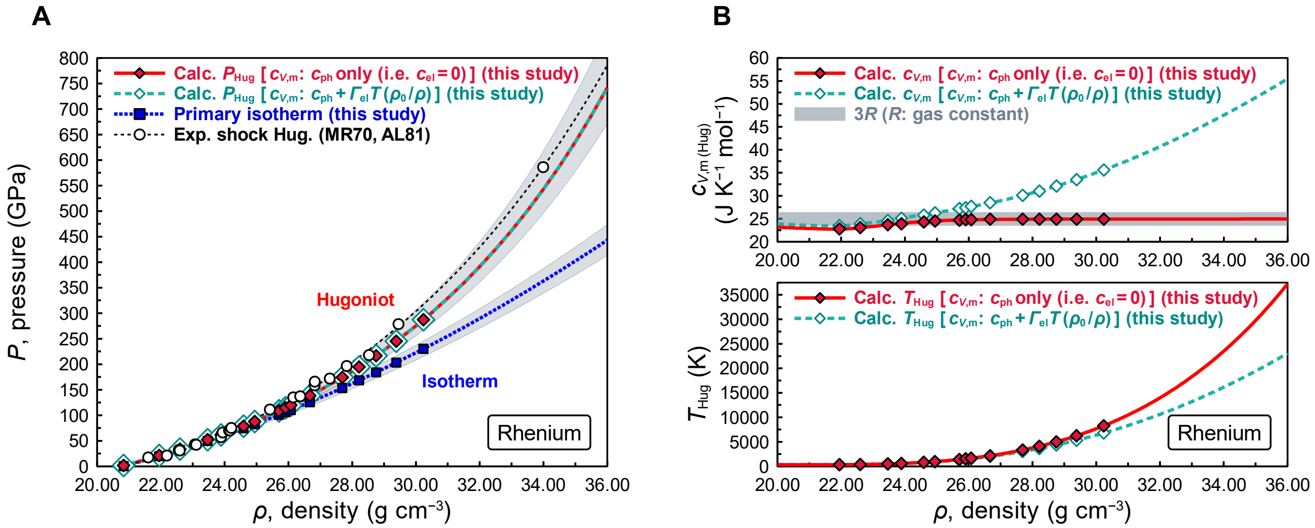

Fig. S19. (**A**) The calibrated isotherm (blue dotted curve with squares) and calculated shock Hugoniot (red solid curve with diamonds and green dashed curve) for rhenium. The shaded area represents the 1σ uncertainty of each curve. The red ($c_{V,\mathrm{m,DM\text{-}zero}}$ model) and green ($c_{V,\mathrm{m,DM\text{-}LTD}}$ model) curves are derived from two different c_{el} models. The open circle symbols and black dotted line represent the experimental shock compressional data and shock Hugoniot (*29, 30*). (**B**) Comparison of calculated $c_{V,\mathrm{m}}$ and T_{Hug} of rhenium on the Hugoniot curves based on $c_{V,\mathrm{m,DM\text{-}zero}}$ (red diamond) and $c_{V,\mathrm{m,DM\text{-}LTD}}$ (green diamond) models. The gray bold line represents $3R$ (where R is the gas constant), converged value of the contribution of phonons to heat capacity derived from the Debye model. The parameters used for calculation are given in Table S2.

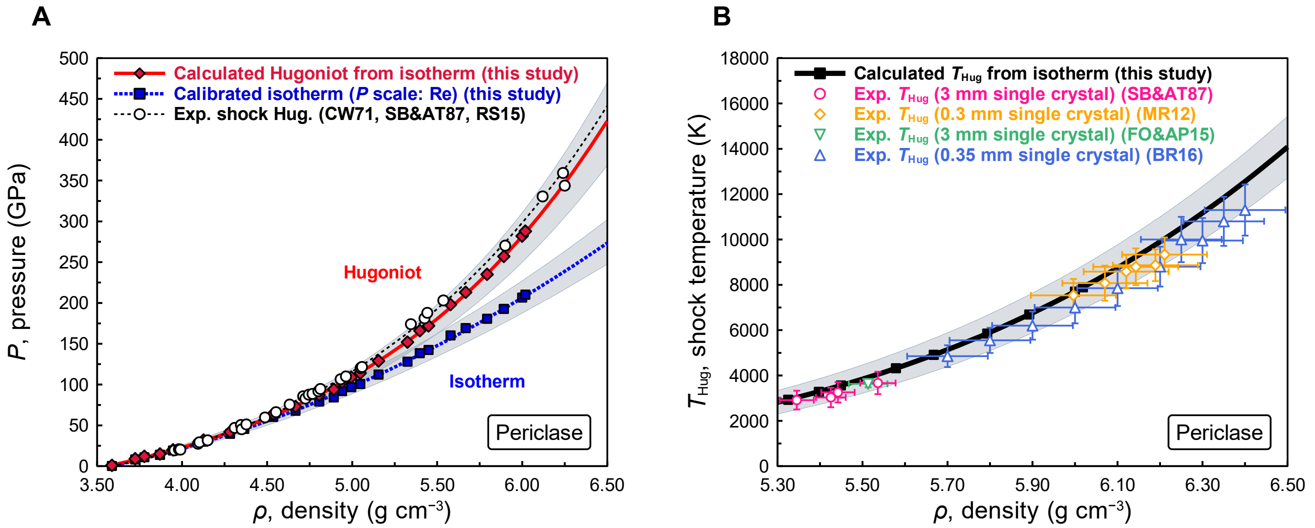

Fig. S20. (**A**) The calibrated isotherm (blue dotted curve with squares) of MgO based on our rhenium scale and calculated shock Hugoniot (red solid curve with diamonds). The shaded area represents the 1σ uncertainty of each curve. The black dotted curve and open circle symbols represent the shock Hugoniot and experimental shock compression data (*47, 79, 80*). (**B**) Calculated T_{Hug} of MgO. The T_{Hug} was derived assuming $c_{\mathrm{el}} = 0$ for the molar heat capacity, $c_{V,\mathrm{m}}$. The detailed parameters used for calculation are given in Table S2. Black squares are the calculated T_{Hug} corresponding to the red diamonds in (A). Other colored symbols are the experimentally measured T_{Hug} of MgO (B1 structure) from previous studies [SB&AT89 (*47*), MR12 (*48*), FO&AP15 (*49*), BR16 (*50*)]. The shaded area around the curve represents the 1σ uncertainty.

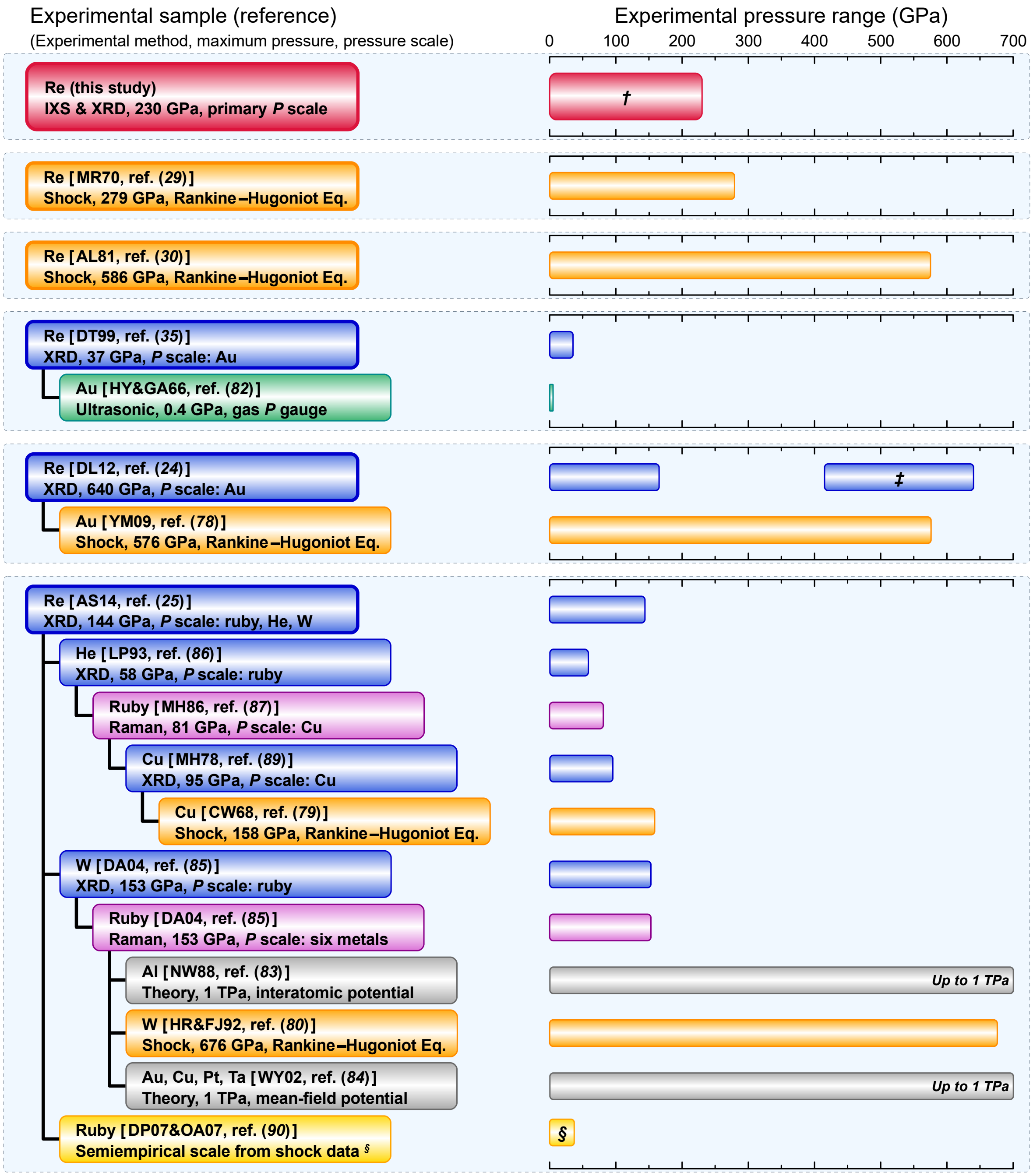

Fig. S21. Experimental methods for the compression of rhenium in this and previous studies (*24, 25, 29, 30, 35*) with pressure scales used in those studies (*78–80, 82–87, 89, 90*). Colors indicate the experimental method; red: IXS and XRD with DAC, yellow: shock compression measurement, light yellow: semiempirical scale from shock compression data (e.g., *29, 30, 80*), blue: XRD with DAC, green: US with gas compression, magenta: Raman spectroscopy with DAC, gray: theoretical work. Experimental conditions are also given within the boxes. Each system diagram represents the flow of the secondary and/or primary pressure scales based on the experiments. *Notes*; †; The highest experimental pressures by using previous pressure scales for rhenium are 274 GPa (*29, 30*), 285 GPa (*25*), 300 GPa (*31*), and 380 GPa (*24*). ‡; the experimental pressure achieved by the work was discussed in refs. (*26*). §: this ruby scale re-evaluated from numerous shock compression data (e.g., *29, 30, 80*) has been used to calibrate only for low pressure data up to 37 GPa in ref. (*25*).

Supplementary Tables

Table S1. Results of acoustic velocity measurement and calibrated pressures for rhenium.

Run-No.	ρ, density ($g\ cm^{-3}$)	v_p, compressional wave velocity ($km\ s^{-1}$)	v_s, shear wave velocity ($km\ s^{-1}$)	$Q_{max}(v_p)$ (nm^{-1})	$Q_{max}(v_s)$ (nm^{-1})	P, calibrated pressure (GPa) §
IXS-Re-01 *	22.60 (±0.08)	5.75 (±0.09)	3.72 (±0.34)	10.5 (±0.4)	11.2 (±1.2)	32 (±3)
IXS-Re-02 *	23.46 (±0.09)	6.07 (±0.12)	3.68 (±0.47)	10.2 (±0.4)	10.7 (±1.4)	50 (±4)
IXS-Re-03 *	24.59 (±0.10)	6.54 (±0.12)	4.14 (±0.37)	10.6 (±0.4)	11.8 (±0.6)	74 (±6)
IXS-Re-04 *	24.94 (±0.12)	6.61 (±0.09)	4.28 (±0.58)	11.6 (±0.4)	11.7 (±1.8)	83 (±6)
IXS-Re-05 *	25.91 (±0.09)	6.88 (±0.12)	-	11.2 (±0.4)	-	106 (±8)
IXS-Re-06 *	26.67 (±0.13)	7.01 (±0.11)	-	12.4 (±0.6)	-	126 (±9)
IXS-Re-07 *	27.69 (±0.18)	7.45 (±0.13)	4.43 (±0.17)	12.4 (±0.4)	12.7 (±1.1)	153 (±11)
IXS-Re-08 *	28.22 (±0.10)	7.74 (±0.23)	4.72 (±0.24)	12.3 (±0.4)	12.8 (±1.8)	168 (±12)
IXS-Re-09 *	28.75 (±0.16)	7.79 (±0.09)	4.83 (±0.63)	12.4 (±0.4)	11.8 (±3.5)	184 (±13)
IXS-Re-10 *	29.38 (±0.13)	8.02 (±0.18)	5.12 (±0.24)	12.8 (±0.4)	11.9 (±0.6)	203 (±15)
IXS-Re-11 *	29.65 (±0.10)	8.09 (±0.13)	-	12.8 (±0.2)	-	212 (±15)
IXS-Re-12 *	30.24 (±0.16)	8.38 (±0.20)	5.35 (±0.26)	12.6 (±0.4)	11.6 (±1.1)	230 (±17)
IXS-Re-13 †	21.95 (±0.10)	5.56 (±0.07)	3.52 (±0.41)	10.9 (±0.4)	11.1 (±1.2)	20 (±3)
IXS-Re-14 †	23.89 (±0.10)	6.22 (±0.15)	3.64 (±0.20)	11.5 (±0.2)	11.3 (±0.5)	59 (±5)
IXS-Re-15 †	25.70 (±0.11)	6.81 (±0.14)	3.86 (±0.25)	11.7 (±0.3)	11.3 (±0.7)	101 (±7)
IXS-Re-16 †	26.08 (±0.09)	7.08 (±0.13)	4.13 (±0.19)	11.4 (±0.2)	10.7 (±0.3)	110 (±8)
IXS-Re-foil ‡	20.83 (±0.07)	5.34 (±0.04)	2.96 (±0.09)	10.1 (±0.3)	12.7 (±1.1)	0.5 (±0.5)

Note: * Direct compression without pressure medium. † Quasi-hydrostatic compression with periclase (MgO) pressure medium. ‡ Pre-compressed foil in air. § Pressures were derived from v_p, v_s, and ρ with the K-primed Mie–Grüneisen–Debye model (details are in the Methods section, and the parameters of the equation of state for rhenium are in Table S2).

Table S2. Parameters of *K*-primed MGD EoS of rhenium, iron, and MgO.

	Rhenium (Re) (hcp-phase)	Iron (Fe) (hcp-phase)	Periclase (MgO) (B1-phase)
	Equation of state (EoS), *K*-primed Mie–Grüneisen–Debye (MGD) model (*refs. 1, 37–39*)		
ρ_0 ($g\ cm^{-3}$)	20.8 (±0.1)	8.25 (±0.05)	3.58 (±0.03)
K_0 (GPa)	340 (±9)	162 (±5)	159 (±6)
K'_0 ($= \partial K_0/\partial P_0$)	3.25 (±0.12)	5.12 (±0.08)	3.79 (±0.08)
K'_∞ ($= \partial K_\infty/\partial P_\infty$)	2.15 (±0.11)	2.55 (±0.09)	2.29 (±0.12)
	Grüneisen parameter, Al'tshuler fixed γ_∞ model (*ref. 56*) : $\gamma=\gamma_\infty+(\gamma_0-\gamma_\infty)(\rho_0/\rho)^q$		
Θ_0 (K)	369 (±5)	515 (±21)	760 (±135)
γ_0	1.94 (±0.31)	1.97 (±0.16)	1.53 (±0.26)
γ_∞	$(3K'_\infty-1)/6$ (fixed) *	$(3K'_\infty-1)/6$ (fixed) *	$(3K'_\infty-1)/6$ (fixed) *
q	0.53 (±0.30)	0.37 (±0.24)	0.44 (±0.68)
	Molar heat capacity, sum of phonon and electron contributions model (*ref. 57*) : $c_{V,m}=c_{ph}+c_{el}$		
$c_{V,m}$ ($J\ K^{-1}\ mol^{-1}$)	c_{ph} (DM) only (i.e. $c_{el}=0$)	c_{ph} (DM) + c_{el} (FEM-8)	c_{ph} (DM) only (i.e. $c_{el}=0$)
	Isothermal compressional wave velocity, Birch's law (*ref. 34*) : $v_p=v_{p,0}+(\partial v_p/\partial\rho)(\rho-\rho_0)$		
$v_{p,0}$ ($km\ s^{-1}$)	5.30 (±0.04)	6.14 (fixed) †	9.85 (fixed) §
$\partial v_p/\partial\rho$ ($m^4\ kg^{-1}\ s^{-1}$)	0.313 (±0.002)	1.16 (fixed) †	2.27 (fixed) §

Note: * Theoretical constraint of the Grüneisen parameter in the *K*-primed MGD model (*ref. 39*). † Fixed to the reference data of ρ–v_p (*ref. 8*). § Calculated from the reference data of ρ–v_s (*ref. 58*) with the EoS parameters (this study). *Abbreviations*: hcp: hexagonal close-packed, B1: rock salt type cubic structure, DM: Debye model, FEM-8: free electron model with eight valence electrons.

Table S3. Results of uncertainty analysis.

Run-No.	σ (ρ), density uncertainty			σ (v_p), compressional wave velocity uncertainty				σ (v_s), shear wave velocity uncertainty				Pressure medium
	EX	LS	DG	EX	PO	LS	TP	EX	PO	LS	TP	
IXS-Re-01	0.4%	1.9%	0.5%	1.5%	0.3%	2.2%	0.3%	9.0%	1.1%	1.7%	0.3%	-
IXS-Re-02	0.4%	1.7%	0.5%	2.0%	0.8%	2.0%	0.3%	12.7%	1.1%	1.5%	0.3%	-
IXS-Re-03	0.4%	1.8%	0.5%	1.9%	0.9%	2.0%	0.2%	9.0%	1.3%	1.5%	0.2%	-
IXS-Re-04	0.5%	1.5%	0.5%	1.4%	0.7%	1.4%	0.3%	13.5%	0.7%	1.1%	0.3%	-
IXS-Re-05	0.4%	1.6%	0.5%	1.7%	0.8%	1.5%	0.1%		-			-
IXS-Re-06	0.5%	1.6%	0.5%	1.5%	0.7%	1.6%	0.1%		-			-
IXS-Re-07	0.7%	1.3%	0.5%	1.8%	0.8%	1.4%	0.1%	3.9%	0.4%	1.1%	0.1%	-
IXS-Re-08	0.3%	1.3%	0.5%	2.9%	0.7%	1.6%	0.1%	5.0%	0.3%	1.2%	0.1%	-
IXS-Re-09	0.5%	1.4%	0.5%	1.2%	0.9%	1.5%	0.1%	13.0%	0.5%	1.1%	0.1%	-
IXS-Re-10	0.5%	1.3%	0.5%	2.3%	0.8%	1.5%	0.1%	4.7%	0.3%	1.2%	0.1%	-
IXS-Re-11	0.3%	1.2%	0.5%	1.6%	0.8%	1.4%	0.1%		-			-
IXS-Re-12	0.5%	1.1%	0.5%	2.4%	0.9%	1.5%	0.1%	4.9%	0.2%	1.1%	0.1%	-
IXS-Re-13	0.5%	0.3%	0.5%	1.3%	0.1%	0.6%	0.4%	11.7%	0.2%	0.4%	0.4%	MgO
IXS-Re-14	0.4%	0.6%	0.5%	2.3%	0.3%	0.6%	0.1%	5.5%	0.2%	0.4%	0.1%	MgO
IXS-Re-15	0.4%	0.5%	0.5%	2.0%	0.4%	0.6%	0.1%	6.4%	0.1%	0.5%	0.1%	MgO
IXS-Re-16	0.4%	0.6%	0.5%	1.8%	0.5%	0.9%	0.1%	4.5%	0.2%	0.7%	0.1%	MgO
IXS-Re-foil	0.3%	1.0%	0.0%	0.7%	0.0%	1.3%	0.1%	3.7%	1.1%	1.0%	0.1%	in air

Abbreviations: EX: experimental error, LS: lattice strain, DG: density gradient in the sample region irradiated by x-ray beam with tails, PO: preferred orientation, TP: thermodynamic property, MgO: periclase (magnesium oxide).

Table S4. Results of simultaneous compression experiment for rhenium, iron, and MgO.

Run-No.	P, calibrated pressure (GPa) *	Rhenium (Re) (hcp-phase) Lattice param. a (nm)	c (nm)	ρ, density (g cm^{-3})	Iron (Fe) (hcp-phase) Lattice param. a (nm)	c (nm)	ρ, density (g cm^{-3})	Periclase (MgO) (B1-ph.) Latt. param. a (nm)	ρ, density (g cm^{-3})
XRD-Re-01	0.3 (±0.4)	0.2762	0.4496	20.82 (±0.03)	0.2862 †		7.91 (±0.01) †	0.4210	3.59 (±0.02)
XRD-Re-02	8 (±2)	0.2746	0.4448	21.29 (±0.04)	0.2822 †		8.25 (±0.01) †	0.4158	3.72 (±0.02)
XRD-Re-03	11 (±2)	0.2740	0.4434	21.45 (±0.06)	0.2809 †		8.37 (±0.01) †	0.4138	3.78 (±0.02)
XRD-Re-04	14 (±2)	0.2739	0.4405	21.61 (±0.03)	0.2470	0.3961	8.86 (±0.01)	0.4105	3.87 (±0.02)
XRD-Re-05	19 (±3)	0.2732	0.4366	21.91 (±0.04)	0.2460	0.3913	9.04 (±0.02)	0.4078	3.95 (±0.02)
XRD-Re-06	30 (±3)	0.2701	0.4353	22.48 (±0.04)	0.2432	0.3857	9.39 (±0.03)	0.4018	4.13 (±0.02)
XRD-Re-07	40 (±4)	0.2688	0.4303	22.98 (±0.07)	0.2408	0.3794	9.74 (±0.05)	0.3969	4.28 (±0.03)
XRD-Re-08	46 (±4)	0.2671	0.4302	23.27 (±0.07)	0.2389	0.3780	9.93 (±0.04)	0.3943	4.37 (±0.03)
XRD-Re-09	60 (±5)	0.2649	0.4246	23.96 (±0.09)	0.2362	0.3711	10.35 (±0.05)	0.3894	4.54 (±0.02)
XRD-Re-10	68 (±5)	0.2633	0.4238	24.30 (±0.08)	0.2354	0.3696	10.45 (±0.05)	0.3857	4.67 (±0.02)
XRD-Re-11	79 (±6)	0.2611	0.4226	24.79 (±0.09)	0.2324	0.3689	10.75 (±0.05)	0.3819	4.81 (±0.02)
XRD-Re-12	84 (±6)	0.2604	0.4211	25.01 (±0.07)	0.2314	0.3677	10.88 (±0.04)	0.3797	4.89 (±0.03)
XRD-Re-13	92 (±7)	0.2597	0.4178	25.34 (±0.05)	0.2306	0.3671	10.97 (±0.04)	0.3784	4.94 (±0.02)
XRD-Re-14	97 (±7)	0.2586	0.4182	25.53 (±0.10)	0.2297	0.3657	11.10 (±0.05)	0.3770	4.99 (±0.02)
XRD-Re-15	101 (±7)	0.2577	0.4186	25.69 (±0.11)	0.2292	0.3644	11.18 (±0.04)	0.3757	5.05 (±0.02)
XRD-Re-16	112 (±8)	0.2564	0.4152	26.15 (±0.11)	0.2275	0.3618	11.43 (±0.05)	0.3731	5.16 (±0.02)
XRD-Re-17	128 (±9)	0.2546	0.4118	26.76 (±0.11)	0.2259	0.3585	11.71 (±0.05)	0.3691	5.33 (±0.02)
XRD-Re-18	138 (±10)	0.2542	0.4072	27.14 (±0.12)	0.2251	0.3561	11.87 (±0.05)	0.3674	5.40 (±0.02)
XRD-Re-19	142 (±10)	0.2529	0.4090	27.29 (±0.10)	0.2241	0.3544	12.03 (±0.05)	0.3662	5.45 (±0.03)
XRD-Re-20	160 (±11)	0.2515	0.4041	27.93 (±0.11)	0.2224	0.3528	12.27 (±0.06)	0.3634	5.58 (±0.02)
XRD-Re-21	169 (±12)	0.2503	0.4035	28.24 (±0.11)	0.2216	0.3500	12.46 (±0.05)	0.3615	5.67 (±0.02)
XRD-Re-22	181 (±13)	0.2490	0.4023	28.63 (±0.11)	0.2203	0.3479	12.68 (±0.04)	0.3588	5.79 (±0.02)
XRD-Re-23	193 (±14)	0.2480	0.3999	29.04 (±0.11)	0.2190	0.3478	12.84 (±0.05)	0.3568	5.89 (±0.04)
XRD-Re-24	207 (±15)	0.2471	0.3964	29.49 (±0.12)	0.2182	0.3459	13.00 (±0.07)	0.3547	6.00 (±0.02)
XRD-Re-25	210 (±15)	0.2466	0.3966	29.60 (±0.12)	0.2180	0.3453	13.04 (±0.06)	0.3543	6.02 (±0.02)

Note: * Pressure was calibrated by the K-primed Mie–Grüneisen–Debye equation of state for rhenium (Table S2).
† Body-centered cubic (bcc) phase. *Abbreviations*: hcp: hexagonal close-packed, B1: rock salt type cubic structure.

Table S5. Calculated shock Hugoniot for hcp-iron.

Iron (Fe)			P_{Hug}, calculated shock pressure, and T_{Hug}, calculated shock temperature *					
			$c_{V,m}$: c_{ph} (DM) + c_{el} (FEM-8)		$c_{V,m}$: c_{ph} (DM) only (i.e. c_{el} = 0)		$c_{V,m}$: c_{ph} (DM) + $\Gamma_{el} T(\rho_0/\rho)$ †	
Run-No.	ρ (g cm^{-3})	$P_{300\ K}$ (GPa)	P_{Hug} (GPa)	T_{Hug} (K)	P_{Hug} (GPa)	T_{Hug} (K)	P_{Hug} (GPa)	T_{Hug} (K)
XRD-Re-01	7.91	0.3	2 (±1) §	280 (±1) §	2 (±1) §	280 (±1) §	2 (±1) §	290 (±1) §
XRD-Re-02	8.25	8	10 (±2) §	340 (±3) §	10 (±2) §	350 (±3) §	10 (±2) §	350 (±3) §
XRD-Re-03	8.37	11	13 (±2) §	370 (±10) §	13 (±2) §	380 (±10) §	13 (±2) §	370 (±10) §
XRD-Re-04	8.86	14	16 (±3)	480 (±50)	17 (±3)	510 (±50)	17 (±3)	500 (±50)
XRD-Re-05	9.04	19	22 (±3)	530 (±70)	22 (±3)	580 (±70)	23 (±3)	560 (±70)
XRD-Re-06	9.39	30	34 (±4)	670 (±110)	35 (±4)	730 (±110)	35 (±4)	700 (±110)
XRD-Re-07	9.74	40	45 (±5)	850 (±170)	46 (±5)	950 (±170)	46 (±5)	890 (±170)
XRD-Re-08	9.93	46	53 (±5)	990 (±200)	53 (±5)	1120 (±200)	54 (±5)	1040 (±200)
XRD-Re-09	10.35	60	71 (±7)	1400 (±270)	72 (±7)	1590 (±270)	73 (±7)	1440 (±270)
XRD-Re-10	10.45	68	80 (±7)	1530 (±300)	81 (±7)	1750 (±300)	82 (±7)	1560 (±300)
XRD-Re-11	10.75	79	95 (±8)	1960 (±360)	97 (±8)	2260 (±360)	98 (±8)	1970 (±360)
XRD-Re-12	10.88	84	103 (±8)	2180 (±390)	105 (±8)	2530 (±390)	105 (±8)	2180 (±390)
XRD-Re-13	10.97	92	112 (±9)	2360 (±410)	115 (±9)	2740 (±410)	115 (±9)	2340 (±410)
XRD-Re-14	11.10	97	120 (±9)	2620 (±440)	123 (±9)	3050 (±440)	123 (±9)	2570 (±440)
XRD-Re-15	11.18	101	126 (±9)	2820 (±460)	129 (±9)	3290 (±460)	129 (±9)	2740 (±460)
XRD-Re-16	11.43	112	144 (±10)	3450 (±530)	148 (±10)	4050 (±530)	148 (±10)	3290 (±530)
XRD-Re-17	11.71	128	169 (±12)	4290 (±610)	174 (±12)	5070 (±610)	174 (±12)	3970 (±610)
XRD-Re-18	11.87	138	186 (±13)	4850 (±650)	192 (±13)	5750 (±650)	192 (±13)	4420 (±650)
XRD-Re-19	12.03	142	198 (±15)	5460 (±700)	204 (±15)	6520 (±700)	204 (±15)	4890 (±700)
XRD-Re-20	12.27	160	229 (±17)	6480 (±780)	236 (±17)	7790 (±780)	236 (±17)	5660 (±780)
XRD-Re-21	12.46	169	249 (±19)	7360 (±850)	257 (±19)	8910 (±850)	257 (±19)	6300 (±850)
XRD-Re-22	12.68	181	276 (±21)	8470 (±920)	284 (±21)	10340 (±920)	285 (±21)	7100 (±920)
XRD-Re-23	12.84	193	301 (±23)	9350 (±980)	310 (±23)	11490 (±980)	310 (±23)	7710 (±980)
XRD-Re-24	13.00	207	329 (±25)	10330 (±1040)	339 (±25)	12800 (±1040)	339 (±25)	8380 (±1040)
XRD-Re-25	13.04	210	336 (±25)	10580 (±1060)	346 (±25)	13130 (±1060)	346 (±25)	8550 (±1060)

Note: * P_{Hug} and T_{Hug} were calculated based on the parameters of *K*-primed Mie–Grüneisen–Debye (MGD) equation of state (EoS) for hexagonal close-packed (hcp) iron (Table S2) and the experimental conditions of the simultaneous compression (Table S4) with the experimental shock compression data of iron (*ref. 42*). † Γ_{el} = 4.90 (mJ K^{-2} mol^{-1}), fixed to the reference data (*ref. 59*).
§ P_{Hug} and T_{Hug} for hcp-iron were estimated from the parameters of *K*-primed MGD-EoS for hcp-iron (Table S2), while the experimentally observed phase at ambient temperature was body-centered cubic iron (Table S4).
Abbreviations: DM: Debye model, FEM-8: free electron model with eight valence electrons.

Table S6. Pressure–density relations for hcp-iron and PREM.

Earth's model (PREM)	P, pressure (GPa)	ρ-PREM (g cm^{-3})	Hexagonal close-packed (hcp) iron (this study) ρ, density (g cm^{-3}) 300 K	6000 K	9000 K	hcp-iron (DA06) ρ, density (g cm^{-3}) 300 K	6000 K
Lower mantle	25	4.41	9.26 (±0.05)	-	-	9.28	-
Lower mantle	50	4.76	10.02 (±0.05)	8.40 (±0.06)	-	9.98	-
Lower mantle	75	5.04	10.64 (±0.06)	9.25 (±0.08)	8.27 (±0.08)	10.55	8.90
Lower mantle	100	5.29	11.19 (±0.07)	9.93 (±0.09)	9.09 (±0.10)	11.04	9.70
Mantle (CMB)	125	5.50	11.68 (±0.07)	10.52 (±0.11)	9.76 (±0.12)	11.48	10.31
Outer (CMB)	125	9.74	11.68 (±0.07)	10.52 (±0.11)	9.76 (±0.12)	11.48	10.31
Outer core	150	10.11	12.13 (±0.08)	11.04 (±0.12)	10.34 (±0.13)	11.87	10.82
Outer core	175	10.46	12.54 (±0.08)	11.51 (±0.12)	10.85 (±0.14)	12.24	11.28
Outer core	200	10.78	12.93 (±0.08)	11.94 (±0.13)	11.32 (±0.15)	12.58	11.68
Outer core	225	11.08	13.29 (±0.08)	12.34 (±0.14)	11.75 (±0.16)	12.90	12.06
Outer core	250	11.37	13.64 (±0.09)	12.72 (±0.14)	12.15 (±0.16)	13.20	12.40
Outer core	275	11.64	13.97 (±0.09)	13.08 (±0.14)	12.53 (±0.17)	13.49	12.72
Outer core	300	11.89	14.28 (±0.09)	13.42 (±0.14)	12.89 (±0.17)	13.76	13.03
Outer (ICB)	330	12.19	14.65 (±0.10)	13.81 (±0.14)	13.29 (±0.17)	14.07	13.37
Inner (ICB)	330	12.79	14.65 (±0.10)	13.81 (±0.14)	13.29 (±0.17)	14.07	13.37
Inner core	365	13.12	15.05 (±0.11)	14.24 (±0.14)	13.74 (±0.17)	14.42	13.75

Note: * Densities were derived from the *K*-primed Mie–Grüneisen–Debye model using the parameters for hcp-iron and the molar electronic heat capacity by the free electron model with eight valence electrons (FEM-8) (details are in the Methods section, and the parameters of the equation of state for hcp-iron are in Table S2).
Abbreviations: PREM: preliminary reference Earth model (*ref. 9*), CMB: core–mantle boundary, ICB: inner core boundary, DA06: (*ref. 5*).

Table S7. Isotherms by the *K*-primed MGD EoS for hcp-iron.

ρ, density (g cm^{-3})	*P*, pressure (GPa) *										
	300 K	1000 K	2000 K	3000 K	6000 K	9000 K	12000 K	15000 K	18000 K	21000 K	24000 K
8.25	0	5	13	21	46	74	105	138	174	212	253
8.50	5	10	18	26	52	81	112	146	183	221	263
8.75	11	16	24	33	59	89	120	155	192	231	273
9.00	17	23	31	39	67	97	129	164	202	242	284
9.25	25	30	38	47	75	105	138	174	212	253	296
9.50	32	38	46	55	84	115	148	184	223	265	308
9.75	40	46	55	64	93	124	159	195	235	277	321
10.00	49	55	64	73	103	135	170	207	247	290	335
10.25	59	65	74	83	113	146	181	219	260	303	349
10.50	69	75	84	94	124	158	194	232	273	317	364
10.75	80	86	95	105	136	170	206	246	287	332	379
11.00	91	97	107	117	148	183	220	260	302	347	395
11.25	103	109	119	129	161	196	234	274	317	363	411
11.50	116	122	132	142	175	210	249	290	333	379	428
11.75	129	135	145	156	189	225	264	305	350	396	446
12.00	143	149	159	170	204	240	280	322	367	414	464
12.25	157	164	174	185	219	256	296	339	384	433	483
12.50	173	179	189	200	235	273	314	357	403	451	503
12.75	188	195	205	217	252	290	331	375	422	471	523
13.00	205	211	222	233	269	308	350	394	441	491	544
13.25	222	228	239	251	287	327	369	414	462	512	565
13.50	240	246	257	269	306	346	389	434	483	534	587
13.75	258	265	276	288	325	366	409	455	504	556	610
14.00	277	284	295	307	345	386	430	477	526	578	633

Note: * Pressures were derived from the *K*-primed Mie–Grüneisen–Debye model using the parameters for hexagonal close-packed (hcp) iron (details are in the Methods section, and the parameters of the equation of state for hcp-iron are in Tables S2, S8, and S9).

Table S8. Molar phonon heat capacity for hcp-iron by the Debye model.

ρ, density (g cm^{-3})	c_{ph} (DM), molar phonon heat capacity by the Debye model (J K^{-1} mol^{-1}) *										
	300 K	1000 K	2000 K	3000 K	6000 K	9000 K	12000 K	15000 K	18000 K	21000 K	24000 K
8.25	21.622	24.616	24.861	24.907	24.934	24.939	24.941	24.942	24.942	24.943	24.943
8.50	21.254	24.575	24.851	24.902	24.933	24.939	24.941	24.942	24.942	24.943	24.943
8.75	20.864	24.532	24.840	24.897	24.932	24.938	24.940	24.942	24.942	24.942	24.943
9.00	20.454	24.485	24.828	24.892	24.930	24.938	24.940	24.941	24.942	24.942	24.943
9.25	20.025	24.434	24.815	24.886	24.929	24.937	24.940	24.941	24.942	24.942	24.942
9.50	19.577	24.380	24.801	24.880	24.927	24.936	24.939	24.941	24.942	24.942	24.942
9.75	19.112	24.321	24.786	24.873	24.926	24.936	24.939	24.941	24.941	24.942	24.942
10.00	18.632	24.259	24.770	24.866	24.924	24.935	24.939	24.940	24.941	24.942	24.942
10.25	18.137	24.193	24.753	24.858	24.922	24.934	24.938	24.940	24.941	24.942	24.942
10.50	17.629	24.123	24.735	24.850	24.920	24.933	24.938	24.940	24.941	24.941	24.942
10.75	17.111	24.049	24.715	24.842	24.918	24.932	24.937	24.939	24.941	24.941	24.942
11.00	16.584	23.970	24.695	24.832	24.916	24.931	24.936	24.939	24.940	24.941	24.942
11.25	16.050	23.887	24.673	24.823	24.913	24.930	24.936	24.939	24.940	24.941	24.941
11.50	15.510	23.799	24.650	24.812	24.911	24.929	24.935	24.938	24.940	24.941	24.941
11.75	14.967	23.706	24.626	24.801	24.908	24.928	24.934	24.938	24.939	24.940	24.941
12.00	14.423	23.610	24.600	24.790	24.905	24.926	24.934	24.937	24.939	24.940	24.941
12.25	13.879	23.508	24.573	24.778	24.902	24.925	24.933	24.937	24.939	24.940	24.941
12.50	13.338	23.401	24.545	24.765	24.899	24.923	24.932	24.936	24.938	24.940	24.941
12.75	12.800	23.290	24.515	24.752	24.895	24.922	24.931	24.936	24.938	24.939	24.940
13.00	12.268	23.174	24.483	24.737	24.892	24.920	24.930	24.935	24.938	24.939	24.940
13.25	11.744	23.053	24.451	24.723	24.888	24.919	24.930	24.935	24.937	24.939	24.940
13.50	11.228	22.927	24.416	24.707	24.884	24.917	24.929	24.934	24.937	24.939	24.940
13.75	10.722	22.796	24.380	24.691	24.880	24.915	24.927	24.933	24.936	24.938	24.939
14.00	10.227	22.660	24.343	24.674	24.876	24.913	24.926	24.933	24.936	24.938	24.939

Note: * c_{ph} (DM) were derived from the Debye model (DM) using the parameters for hexagonal close-packed (hcp) iron (details are in the Methods section, and the parameters of the equation of state for hcp-iron are in Table S2).

Table S9. Molar electronic heat capacity for hcp-iron by the FEM-8.

ρ, density (g cm^{-3})	c_{el} (FEM-8), molar electronic heat capacity by the free electron model with eight valence electrons (J K^{-1} mol^{-1}) *										
	300 K	1000 K	2000 K	3000 K	6000 K	9000 K	12000 K	15000 K	18000 K	21000 K	24000 K
8.25	0.243	0.924	1.897	2.870	5.785	8.691	11.585	14.462	17.317	20.145	22.941
8.50	0.238	0.906	1.860	2.813	5.671	8.520	11.358	14.180	16.982	19.758	22.504
8.75	0.234	0.889	1.824	2.760	5.562	8.358	11.142	13.912	16.662	19.389	22.086
9.00	0.230	0.872	1.790	2.708	5.459	8.203	10.937	13.656	16.357	19.036	21.688
9.25	0.225	0.857	1.758	2.659	5.360	8.055	10.740	13.411	16.065	18.699	21.306
9.50	0.221	0.841	1.727	2.612	5.266	7.914	10.552	13.177	15.786	18.376	20.941
9.75	0.218	0.827	1.697	2.568	5.176	7.778	10.372	12.953	15.519	18.067	20.591
10.00	0.214	0.813	1.669	2.525	5.089	7.649	10.199	12.738	15.263	17.770	20.255
10.25	0.210	0.800	1.642	2.483	5.006	7.524	10.034	12.532	15.017	17.485	19.933
10.50	0.207	0.787	1.616	2.444	4.927	7.405	9.875	12.334	14.781	17.211	19.623
10.75	0.204	0.775	1.590	2.406	4.850	7.290	9.722	12.144	14.554	16.948	19.324
11.00	0.201	0.763	1.566	2.369	4.776	7.179	9.575	11.961	14.335	16.695	19.037
11.25	0.198	0.752	1.543	2.334	4.706	7.073	9.433	11.784	14.124	16.450	18.760
11.50	0.195	0.741	1.521	2.300	4.637	6.970	9.296	11.614	13.921	16.215	18.492
11.75	0.192	0.730	1.499	2.267	4.571	6.871	9.165	11.450	13.725	15.987	18.235
12.00	0.190	0.720	1.478	2.236	4.508	6.776	9.038	11.292	13.536	15.768	17.985
12.25	0.187	0.710	1.458	2.205	4.446	6.683	8.915	11.139	13.353	15.556	17.744
12.50	0.184	0.701	1.438	2.176	4.387	6.594	8.796	10.991	13.176	15.350	17.511
12.75	0.182	0.692	1.419	2.147	4.329	6.508	8.681	10.848	13.005	15.152	17.286
13.00	0.180	0.683	1.401	2.120	4.274	6.424	8.570	10.709	12.839	14.960	17.067
13.25	0.177	0.674	1.384	2.093	4.220	6.343	8.462	10.575	12.679	14.773	16.856
13.50	0.175	0.666	1.366	2.067	4.168	6.265	8.358	10.445	12.523	14.592	16.650
13.75	0.173	0.658	1.350	2.042	4.117	6.189	8.257	10.318	12.372	14.417	16.451
14.00	0.171	0.650	1.334	2.018	4.068	6.115	8.158	10.196	12.226	14.247	16.258

Note: * c_{el} (FEM-8) were derived from the free electron model with eight valence electrons (FEM-8) using the parameters for hexagonal close-packed (hcp) iron (details are in the Methods section, and the parameters of the equation of state for hcp-iron are in Table S2).

Table S10. Molar electronic heat capacity for hcp-iron by the LTD.

ρ, density (g cm^{-3})	$\Gamma_{el}T(\rho_0/\rho)$, molar electronic heat capacity by the linear temperature dependence model (J K^{-1} mol^{-1}) *										
	300 K	1000 K	2000 K	3000 K	6000 K	9000 K	12000 K	15000 K	18000 K	21000 K	24000 K
8.25	1.470	4.900	9.800	14.700	29.400	44.100	58.800	73.500	88.200	102.900	117.600
8.50	1.427	4.756	9.512	14.268	28.535	42.803	57.071	71.338	85.606	99.874	114.141
8.75	1.386	4.620	9.240	13.860	27.720	41.580	55.440	69.300	83.160	97.020	110.880
9.00	1.348	4.492	8.983	13.475	26.950	40.425	53.900	67.375	80.850	94.325	107.800
9.25	1.311	4.370	8.741	13.111	26.222	39.332	52.443	65.554	78.665	91.776	104.886
9.50	1.277	4.255	8.511	12.766	25.532	38.297	51.063	63.829	76.595	89.361	102.126
9.75	1.244	4.146	8.292	12.438	24.877	37.315	49.754	62.192	74.631	87.069	99.508
10.00	1.213	4.043	8.085	12.128	24.255	36.383	48.510	60.638	72.765	84.893	97.020
10.25	1.183	3.944	7.888	11.832	23.663	35.495	47.327	59.159	70.990	82.822	94.654
10.50	1.155	3.850	7.700	11.550	23.100	34.650	46.200	57.750	69.300	80.850	92.400
10.75	1.128	3.760	7.521	11.281	22.563	33.844	45.126	56.407	67.688	78.970	90.251
11.00	1.103	3.675	7.350	11.025	22.050	33.075	44.100	55.125	66.150	77.175	88.200
11.25	1.078	3.593	7.187	10.780	21.560	32.340	43.120	53.900	64.680	75.460	86.240
11.50	1.055	3.515	7.030	10.546	21.091	31.637	42.183	52.728	63.274	73.820	84.365
11.75	1.032	3.440	6.881	10.321	20.643	30.964	41.285	51.606	61.928	72.249	82.570
12.00	1.011	3.369	6.738	10.106	20.213	30.319	40.425	50.531	60.638	70.744	80.850
12.25	0.990	3.300	6.600	9.900	19.800	29.700	39.600	49.500	59.400	69.300	79.200
12.50	0.970	3.234	6.468	9.702	19.404	29.106	38.808	48.510	58.212	67.914	77.616
12.75	0.951	3.171	6.341	9.512	19.024	28.535	38.047	47.559	57.071	66.582	76.094
13.00	0.933	3.110	6.219	9.329	18.658	27.987	37.315	46.644	55.973	65.302	74.631
13.25	0.915	3.051	6.102	9.153	18.306	27.458	36.611	45.764	54.917	64.070	73.223
13.50	0.898	2.994	5.989	8.983	17.967	26.950	35.933	44.917	53.900	62.883	71.867
13.75	0.882	2.940	5.880	8.820	17.640	26.460	35.280	44.100	52.920	61.740	70.560
14.00	0.866	2.888	5.775	8.663	17.325	25.988	34.650	43.313	51.975	60.638	69.300

Note: * $\Gamma_{el}T(\rho_0/\rho)$ were derived from the linear temperature dependence with Γ_{el} = 4.90 (mJ K^{-2} mol^{-1}) (*ref. 59*) using the parameters for hexagonal close-packed (hcp) iron (details are in the Methods section, and the parameters of the equation of state for hcp-iron are in Table S2).

REFERENCES AND NOTES

1. J. P. Poirier, *Introduction to the Physics of the Earth's Interior* (Cambridge Univ. Press, ed. 2, 2012).

2. B. J. Wood, M. J. Walter, J. Wade, Accretion of the Earth and segregation of its core. *Nature* **441**, 825–833 (2006).

3. W. F. McDonough, Compositional model for the Earth's core, in *Treatise on Geochemistry*, vol. 3, *The Mantle and Core*, H. D. Holland, K. K. Turekian, Eds. (Elsevier, ed. 2, 2014), pp. 559–577.

4. T. Lay, J. Hernlund, B. A. Buffett, Core-mantle boundary heat flow. *Nat. Geosci.* **1**, 25–32 (2008).

5. A. Dewaele, P. Loubeyre, F. Occelli, M. Mezouar, P. I. Dorogokupets, M. Torrent, Quasihydrostatic equation of state of iron above 2 Mbar. *Phys. Rev. Lett.* **97**, 215504 (2006).

6. T. Sakai, S. Takahashi, N. Nishitani, I. Mashino, E. Ohtani, N. Hirao, Equation of state of pure iron and $Fe_{0.9}Ni_{0.1}$ alloy up to 3 Mbar, *Phys. Earth Planet. Inter.* **228**, 114–126 (2014).

7. N. Hirao, Y. Akahama, Y. Ohishi, Equations of state of iron and nickel to the pressure at the center of the Earth. *Matter. Radiat. Extrem.* **7**, 038403 (2022).

8. D. Ikuta, E. Ohtani, H. Fukui, T. Sakai, D. Ishikawa, A. Q. R. Baron, Sound velocity of hexagonal close-packed iron to the Earth's inner core pressure. *Nat. Commun.* **13**, 7211 (2022).

9. A. M. Dziewonski, D. L. Anderson, Preliminary reference Earth model. *Phys. Earth Planet. Inter*. **25**, 297–356 (1981).

10. L. Stixrude, E. Wasserman, R. E. Cohen, Composition and temperature of Earth's inner core. *J. Geophys. Res.* **102**, 24729–24739 (1997).

11. D. Alfe, M. J. Gillan, G. D. Price, Temperature and composition of the Earth's core. *Contemp. Phys.* **48**, 63–80 (2007).

12. S. Anzellini, A. Dewaele, M. Mezouar, P. Loubeyre, G. Morard, Melting of iron at Earth's inner core boundary based on fast x-ray diffraction. *Science* **340**, 464–466 (2013).

13. S. V. Sinogeikin, J. D. Bass, Single-crystal elasticity of pyrope and MgO to 20 GPa by Brillouin scattering in the diamond cell. *Phys. Earth Planet. Inter.* **120**, 43–62 (2000).

14. C. S. Zha, H. K. Mao, R. J. Hemley, Elasticity of MgO and a primary pressure scale to 55 GPa. *Proc. Natl. Acad. Sci. U.S.A.* **97**, 13494–13499 (2000).

15. B. Li, J. Kung, T. Uchida, Y. Wang, Pressure calibration to 20 GPa by simultaneous use of ultrasonic and x-ray techniques. *J. Appl. Phys.* **98**, 013521 (2005).

16. Y. Kono, T. Irifunea, Y. Higo, T. Inoue, A. Barnhoorn, *P V T* relation of MgO derived by simultaneous elastic wave velocity and in situ X-ray measurements: A new pressure scale for the mantle transition region. *Phys. Earth Planet. Inter.* **183**, 196–211 (2010).

17. S. Kamada, H. Fukui, A. Yoneda, H. Gomi, F. Maeda, S. Tsutsui, H. Uchiyama, N. Hirao, D. Ishikawa, A. Q. R. Baron, Elastic constants of single-crystal Pt measured up to 20 GPa based on inelastic X-ray scattering: Implication for the establishment of an equation of state. *C. R. Geosci.* **351**, 236–242 (2019).

18. H. Fukui, A. Yoneda, S. Kamada, H. Uchiyama, S. Tsutsui, N. Hirao, A. Q. R. Baron, Elasticity of single-crystal NaCl under high-pressure: Simultaneous measurement of x-ray inelastic scattering and diffraction. *High Press. Res.* **40**, 465–477 (2020).

19. X. Qi, N. Cai, S. Wang, B. Li, Thermoelastic properties of tungsten at simultaneous high pressure and temperature. *J. Appl. Phys.* **128**, 105105 (2020).

20. M. Murakami, N. Takata, Absolute primary pressure scale to 120 GPa: Toward a pressure benchmark for Earth's lower mantle. *J. Geophys. Res.* **124**, 6581–6588 (2019).

21. G. Shen, Y. Wang, A. Dewaele, C. Wu, D. E. Fratanduono, J. Eggert, S. Klotz, K. F. Dziubek, P. Loubeyre, O. V. Fat'yanov, P. D. Asimow, T. Mashimo, R. M. M. Wentzcovitch; IPPS Task group, Toward an international practical pressure scale: A proposal for an IPPS ruby gauge (IPPS-Ruby2020). *High Press. Res.* **40**, 299–314 (2020).

22. T. S. Duffy, N. Madhusudhan, K. K. M. Lee, Mineralogy of super-Earth planets, in *Treatise on Geophysics*, vol. 2, *Mineral Physics*, G. Schubert, Ed. (Elsevier, ed. 2, 2015), pp. 149–178.

23. R. F. Smith, D. E. Fratanduono, D. G. Braun, T. S. Duffy, J. K. Wicks, P. M. Celliers, S. J. Ali, A. Fernandez-Panella, R. G. Kraus, D. C. Swift, G. W. Collins, J. H. Eggert, Equation of state of iron under core conditions of large rocky exoplanets. *Nat. Astron.* **2**, 452–458 (2018).

24. L. Dubrovinsky, N. A. Dubrovinskaia, V. B. Prakapenka, A. M. Abakumov, Implementation of micro-ball nanodiamond anvils for high-pressure studies above 6 Mbar. *Nat. Commun.* **3**, 1163 (2012).

25. S. Anzellini, A. Dewaele, F. Occelli, P. Loubeyre, M. Mezouar, Equation of state of rhenium and application for ultra high pressure calibration. *J. Appl. Phys.* **115**, 043511 (2014).

26. T. Sakai, T. Yagi, T. Irifune, H. Kadobayashi, N. Hirao, T. Kunimoto, H. Ohfuji, S. Kawaguchi-Imada, Y. Ohishi, S. Tateno, K. Hirose, High pressure generation using double-stage diamond anvil technique: Problems and equations of state of rhenium. *High Press. Res.* **38**, 107–119 (2018).

27. A. Q. R. Baron, High-resolution inelastic x-ray scattering I & II, in *Synchrotron Light Sources and Free-Electron Lasers*, E. J. Jaeschke, S. Khan, J. R. Schneider, J. B. Hastings, Eds. (Springer, 2016), pp. 1643–1757.

28. A. Q. R. Baron, D. Ishikawa, H. Fukui, Y. Nakajima, Auxiliary optics for meV-resolved inelastic x-ray scattering at SPring-8: Microfocus, analyzer masks, soller slit, soller screen, and beam position monitor. *AIP Conf. Proc.* **2054**, 020002 (2019).

29. R. G. McQueen, S. P. Marsh, J. W. Taylor, J. N. Fritz, W. J. Carter, The equation of state of solids from shock wave studies, in *High-Velocity Impact Phenomena*, R. Kinslow, Ed. (Academic Press, 1970), pp. 293–417 and 515–568.

30. L. V. Al’tshuler, A. A. Bakanova, I. P. Dudoladov, E. A. Dynin, R. F. Trunin, B. S. Chekin, Shock adiabatic curves of metals. *J. Appl. Mech. Tech. Phys.* **22**, 145–169 (1981).

31. M.-B. Lv, Y. Cheng, Y.-Y. Qi, G.-F. Ji, C.-G. Piao, Elastic properties and phonon dispersions of rhenium in hexagonal-close-packed structure under pressure from first principles. *Physica B Condens. Matter* **407**, 778–783 (2012).

32. M. H. Manghnani, K. Katahara, E. S. Fisher, Ultrasonic equation of state of rhenium. *Phys. Rev. B* **9**, 1421–1431 (1974).

33. T. Sakamaki, E. Ohtani, H. Fukui, S. Kamada, S. Takahashi, T. Sakairi, A. Takahata, T. Sakai, S. Tsutsui, D. Ishikawa, R. Shiraishi, Y. Seto, T. Tsuchiya, A. Q. R. Baron, Constraints on Earth's inner core composition inferred from measurements of the sound velocity of hcp-iron in extreme conditions. *Sci. Adv.* **2**, e1500802 (2016).

34. F. Birch, Density and composition of mantle and core. *J. Geophys. Res.* **69**, 4377–4388 (1964).

35. T. S. Duffy, G. Shen, D. L. Heinz, J. Shu, Y. Ma, H. K. Mao, R. J. Hemley, A. K. Singh, Lattice strains in gold and rhenium under nonhydrostatic compression to 37 GPa. *Phys. Rev. B* **60**, 15063–15073 (1999).

36. G. Steinle-Neumann, L. Stixrude, R. E. Cohen, First-principles elastic constants for the hcp transition metals Fe, Co, and Re at high pressure. *Phys. Rev. B* **60**, 791–799 (1999).

37. A. Keane, An investigation of finite strain in an isotropic material subjected to hydrostatic pressure and its seismological applications. *Aust. J. Phys.* **7**, 322–333 (1954).

38. F. D. Stacey, The *K*-primed approach to high-pressure equations of state. *Geophys. J. Int.* **143**, 621–628 (2000).

39. F. D. Stacey, P. M. Davis, High pressure equations of state with applications to the lower mantle and core. *Phys. Earth. Planet. Inter.* **142**, 137–184 (2004).

40. S. D. Jacobsen, C. M. Holl, K. A. Adams, R. A. Fischer, E. S. Martin, C. R. Bina, J. F. Lin, V. B. Prakapenka, A. Kubo, P. Dera, Compression of single-crystal magnesium oxide to 118 GPa and a ruby pressure gauge for helium pressure media. *Am. Mineral.* **93**, 1823–1828 (2008).

41. Y. Ye, V. Prakapenka, Y. Meng, S. H. Shim, Intercomparison of the gold, platinum, and MgO pressure scales up to 140 GPa and 2500 K. *J. Geophys. Res.* **122**, 3450–3464 (2017).

42. J. M. Brown, J. N. Fritz, R. S. Hixson, Hugoniot data for iron. *J. Appl. Phys.* **88**, 5496–5498 (2000).

43. J. D. Bass, B. Svendsen, T. J. Ahrens, The temperature of shock compressed iron, in *High Pressure Research in Mineral Physics*, M. H. Manghnani, Y. Syono, Eds. (Terra Scientific Publishing Company, 1987), pp. 393–402.

44. C. S. Yoo, N. C. Holmes, M. Ross, D. J. Webb, C. Pike, Shock temperatures and melting of iron at Earth core conditions. *Phys. Rev. Lett.* **70**, 3931–3934 (1993).

45. J. Li, Q. Wu, J. Li, T. Xue, Y. Tan, X. Zhou, Y. Zhang, Z. Xiong, Z. Gao, T. Sekine, Shock melting curve of iron: A consensus on the temperature at the Earth's inner core boundary. *Geophys. Res. Lett.* **47**, e2020GL087758 (2020).

46. S.-N. Luo, T. J. Ahrens, Shock-induced superheating and melting curves of geophysically important minerals. *Phys. Earth. Planet. Inter.* **143-144**, 369–386 (2004).

47. B. Svendsen, T. J. Ahrens, Shock-induced temperatures of MgO. *Geophys. J. Int.* **91**, 667–691 (1987).

48. R. S. McWilliams, D. K. Spaulding, J. H. Eggert, P. M. Celliers, D. G. Hicks, R. F. Smith, G. W. Collins, R. Jeanloz, Phase transformations and metallization of magnesium oxide at high pressure and temperature. *Science* **338**, 1330–1333 (2012).

49. O. V. Fat'yanov, P. D. Asimow, Contributed Review: Absolute spectral radiance calibration of fiber-optic shock-temperature pyrometers using a coiled-coil irradiance standard lamp. *Rev. Sci. Instrum.* **86**, 101502 (2015).

50. R. M. Bolis, G. Morard, T. Vinci, A. Ravasio, E. Bambrink, M. Guarguaglini, M. Koenig, R. Musella, F. Remus, J. Bouchet, N. Ozaki, K. Miyanishi, T. Sekine, Y. Sakawa, T. Sano, R. Kodama, F. Guyot, A. Benuzzi-Mounaix, Decaying shock studies of phase transitions in MgO-SiO_2 systems: Implications for the super-Earths' interiors. *Geophys. Res. Lett.* **43**, 9475–9483 (2016).

51. H. Fukui, T. Sakai, T. Sakamaki, S. Kamada, S. Takahashi, E. Ohtani, A. Q. R. Baron, A compact system for generating extreme pressures and temperatures: An application of laser-heated diamond anvil cell to inelastic x-ray scattering. *Rev. Sci. Instrum.* **84**, 113902 (2013).

52. G. Fiquet, J. Badro, F. Guyot, H. Requardt, M. Krisch, Sound velocities in iron to 110 gigapascals. *Science* **291**, 468–471 (2001).

53. D. Antonangeli, F. Occelli, H. Requardt, J. Badro, G. Fiquet, M. Krisch, Elastic anisotropy in textured hcp-iron to 112 GPa from sound wave propagation measurements. *Earth Planet. Sci. Lett.* **225**, 243–251 (2004).

54. Y. Seto, D. Nishio-Hamane, T. Nagai, N. Sata, Development of a software suite on x-ray diffraction experiments. *Rev. High Press. Sci. Technol.* **20**, 269–276 (2010).

55. Y. Seto, Whole pattern fitting for two-dimensional diffraction patterns from polycrystalline materials. *Rev. High Press. Sci. Technol.* **22**, 144–152 (2012).

56. L. V. Al'tshuler, S. E. Brusnikin, E. A. Kuz'menkov, Isotherms and Grüneisen functions for 25 metals. *J. Appl. Mech. Tech. Phys.* **28**, 129–141 (1987).

57. V. N. Zharkov, V. A. Kalinin, *Equations of State for Solids at High Pressures and Temperatures* (Springer, 1971).

58. M. Murakami, Y. Ohishi, N. Hirao, K. Hirose, Elasticity of MgO to 130 GPa: Implications for lower mantle mineralogy. *Earth Planet. Sci. Lett.* **277**, 123–129 (2009).

59. G. R. Stewart, Measurement of low-temperature specific heat. *Rev. Sci. Instrum.* **54**, 1–11 (1983).

60. H. Gomi, K. Ohta, K. Hirose, S. Labrosse, R. Caracas, M. J. Verstraete, J. W. Hernlund, The high conductivity of iron and thermal evolution of the Earth's core. *Phys. Earth Planet. Inter.* **224**, 88–103 (2013).

61. K. Ohta, Y. Kuwayama, K. Hirose, K. Shimizu, Y. Ohishi, Experimental determination of the electrical resistivity of iron at Earth's core conditions, *Nature* **534**, 95–98 (2016).

62. F. Wagle, G. Steinle-Neumann, N. de Koker, Resistivity saturation in liquid iron-light-element alloys at conditions of planetary cores from first principles computations. *C. R. Geosci.* **351**, 154–162 (2019).

63. L. W. Shacklette, Specific heat and resistivity of iron near its Curie point. *Phys. Rev. B* **9**, 3789–3792 (1974).

64. J. M. Brown, R. G. McQueen, Phase transitions, Grüneisen parameter, and elasticity for shocked iron between 77 GPa and 400 GPa. *J. Geophys. Res.* **91**, 7485–7494 (1986).

65. J. M. Walsh, M. H. Rice, R. G. McQueen, F. L. Yarger, Shock-wave compressions of twenty-seven metals. Equations of state of metals. *Phys. Rev.* **108**, 196–216 (1957).

66. W. L. Mao, V. V. Struzhkin, A. Q. R. Baron, S. Tsutsui, C. E. Tommaseo, H.-R. Wenk, M. Y. Hu, P. Chow, W. Sturhahn, J. Shu, R. J. Hemley, D. L. Heinz, H.-K. Mao, Experimental determination of the elasticity of iron at high pressure. *J. Geophys. Res.* **113**, 10.1029/2007JB005229 (2008).

67. G. Mavko, T. Mukerji, J. Dvorkin, *The Rock Physics Handbook: Tools for Seismic Analysis of Porous Media* (Cambridge Univ. Press, ed. 2, 2010).

68. W. Voigt, Ueber die beziehung zwischen den beiden elasticitätsconstanten isotroper körper. *Ann. Phys.* **274**, 573–587 (1889).

69. A. Reuss, Berechnung der fließgrenze von mischkristallen auf grund der plastizitätsbedingung für einkristalle. *Ztschr. Angew. Math. Mech.* **9**, 49–58 (1929).

70. R. Hill, The elastic behaviour of a crystalline aggregate. *Proc. Phys. Soc. A* **65**, 349–354 (1952).

71. A. K. Singh, C. Balasingh, The lattice strains in a specimen (hexagonal system) compressed nonhydrostatically in an opposed anvil high pressure setup. *J. Appl. Phys.* **75**, 4956–4962 (1994).

72. A. K. Singh, C. Balasingh, H. K. Mao, R. J. Hemley, J. Shu, Analysis of lattice strains measured under nonhydrostatic pressure. *J. Appl. Phys.* **83**, 7567–7575 (1998).

73. B. Li, C. Ji, W. Yang, J. Wang, K. Yang, R. Xu, W. Liu, Z. Cai, J. Chen, H. K. Mao, Diamond anvil cell behavior up to 4 Mbar. *Proc. Natl. Acad. Sci. U.S.A.* **115**, 1713–1717 (2018).

74. M. Matsui, S. C. Parker, M. Leslie, The MD simulation of the equation of state of MgO: Application as a pressure calibration standard at high temperature and high pressure. *Am. Mineral.* **85**, 312–316 (2000).

75. A. R. Oganov, M. J. Gillan, G. D. Price, *Ab initio* lattice dynamics and structural stability of MgO. *J. Chem. Phys.* **118**, 10174–10182 (2003).

76. D. Alfe, M. Alfredsson, J. Brodholt, M. J. Gillan, M. D. Towler, R. J. Needs, Quantum Monte Carlo calculations of the structural properties and the B1-B2 phase transition of MgO. *Phys. Rev. B* **72**, 014114 (2005).

77. Z. Wu, R. M. Wentzcovitch, K. Umemoto, B. Li, K. Hirose, J.-C. Zheng, Pressure-volume-temperature relations in MgO: An ultrahigh pressure-temperature scale for planetary sciences applications. *J. Geophys. Res.* **113**, 10.1029/2007JB005275 (2008).

78. M. Yokoo, N. Kawai, K. G. Nakamura, K. Kondo, Y. Tange, T. Tsuchiya, Ultrahigh-pressure scales for gold and platinum at pressures up to 550 GPa. *Phys. Rev. B* **80**, 104114 (2009).

79. W. J. Carter, S. P. Marsh, J. N. Fritz, R. G. McQueen, The equation of state of selected materials for high-pressure references (1968), in *Accurate Characterization of the High-pressure Environment*, E. C. Lloyd, Ed. (U.S. National Bureau of Standards, 1971), pp. 147–158.

80. R. S. Hixson, J. N. Fritz, Shock compression of tungsten and molybdenum. *J. Appl. Phys.* **71**, 1721–1728 (1992).

81. S. Root, L. Shulenburger, R. W. Lemke, D. H. Dolan, T. R. Mattsson, M. P. Desjarlais, Shock response and phase transitions of MgO at planetary impact conditions. *Phys. Rev. Lett.* **115**, 198501 (2015).

82. Y. Hiki, A. V. Granato, Anharmonicity in noble metals; higher order elastic constants. *Phys. Rev.* **144**, 411–419 (1966).

83. W. J. Nellis, J. A. Moriarty, A. C. Mitchell, M. Ross, R. G. Dandrea, N. W. Ashcroft, N. C. Holmes, G. R. Gathers, Metals physics at ultrahigh pressure: Aluminum, copper, and lead as prototypes. *Phys. Rev. Lett.* **60**, 1414–1417 (1988).

84. Y. Wang, R. Ahuja, B. Johansson, Reduction of shock-wave data with mean-field potential approach. *J. Appl. Phys.* **92**, 6616–6620 (2002).

85. A. Dewaele, P. Loubeyre, M. Mezouar, Equations of state of six metals above 94GPa. *Phys. Rev. B* **70**, 094112 (2004).

86. P. Loubeyre, R. LeToullec, J. P. Pinceaux, H. K. Mao, J. Hu, R. J. Hemley, Equation of state and phase diagram of solid ^{4}He from single-crystal x-ray diffraction over a large *P-T* domain. *Phys. Rev. Lett.* **71**, 2272–2275 (1993).

87. H. K. Mao, J. Xu, P. M. Bell, Calibration of the ruby pressure gauge to 800 kbar under quasi-hydrostatic conditions. *J. Geophys. Res.* **91**, 4673–4676 (1986).

88. W. Setyawan, S. Curtarolo, High-throughput electronic band structure calculations: Challenges and tools. *Comput. Mater. Sci.* **49**, 299–312 (2010).

89. H. K. Mao, P. M. Bell, J. W. Shaner, D. J. Steinberg, Specific volume measurements of Cu, Mo, Pd, and Ag and calibration of the ruby R1 fluorescence pressure gauge from 0.06 to 1 Mbar. *J. Appl. Phys.* **49**, 3276–3283 (1978).

90. P. I. Dorogokupets, A. R. Oganov, Ruby, metals, and MgO as alternative pressure scales: A semiempirical description of shock-wave, ultrasonic, x-ray, and thermochemical data at high temperatures and pressures. *Phys. Rev. B* **75**, 024115 (2007).
